# Die gaschromatographische Dampfraumanalyse im Human-Biomonitoring (Headspace-Gaschromatographie): Biomonitoring-Methoden, Konzeptionelle Themen

**DOI:** 10.34865/bihsgcdgt10_3or

**Published:** 2025-09-29

**Authors:** Michael Bader, Bernd Roßbach, Thomas Göen, Elisabeth Eckert, Anja Schäferhenrich, Stefanie Nübler, Wolfgang Gries, Gabriele Leng, Jan Van Pul, Wolfgang Will, Andrea Hartwig, MAK Commission

**Affiliations:** 1 BASF SE. Corporate Health Management Carl-Bosch-Straße 38 67056 Ludwigshafen Deutschland; 2 Institut für Arbeits-, Sozial- und Umweltmedizin, Universitätsmedizin der Johannes Gutenberg-Universität Mainz Obere Zahlbacher Straße 67 55131 Mainz Deutschland; 3 Friedrich-Alexander-Universität Erlangen-Nürnberg. Institut und Poliklinik für Arbeits-, Sozial- und Umweltmedizin Henkestraße 9–11 91054 Erlangen Deutschland; 4 Currenta GmbH & Co. OHG. CUR-SIT-SER-GS-BLM Institut für Biomonitoring Chempark, Geb. Q18 51368 Leverkusen Deutschland; 5 BASF Antwerpen N.V. Haven 725, Scheldelaan 600 2040 Antwerpen Belgien; 6 Institut für Angewandte Biowissenschaften. Abteilung Lebensmittelchemie und Toxikologie. Karlsruher Institut für Technologie (KIT) Adenauerring 20a, Geb. 50.41 76131 Karlsruhe Deutschland; 7 Ständige Senatskommission zur Prüfung gesundheitsschädlicher Arbeitsstoffe. Deutsche Forschungsgemeinschaft, Kennedyallee 40, 53175 Bonn, Deutschland. Weitere Informationen: Ständige Senatskommission zur Prüfung gesundheitsschädlicher Arbeitsstoffe | DFG

**Keywords:** Headspace-Gaschromatographie, HS-GC, Biomonitoring, Urin, Blut, Serum, Plasma, Headspace, Headspace-Technik, Halbwertszeit

## Abstract

The working group “Analyses in Biological Materials” of the German Senate Commission for the Investigation of Health Hazards of Chemical Compounds in the Work Area (MAK Commission) describes the current status of headspace-gas chromatography with respect to its potential applications in human biomonitoring. Particular focus is given to the review and discussion of newly developed methods for headspace sample collection as well as analyte enrichment. The article gives an overview on internationally published headspace methods for the matrices urine, blood, serum and plasma, existing assessment values for headspace parameters, background exposure levels in the non-occupationally exposed general population as well as half-lives of the most prominent hazardous substances measurable by headspace analysis. In addition, critical requirements for and possible pitfalls of the preanalytical phase and of the calibration of headspace methods are also discussed. The review shows that headspace methods have been continuously improved in recent decades and thus continue to make an important contribution to human biomonitoring of occupational and environmental exposure to volatile hazardous compounds.

## 1 Einleitung

Nach einer allgemeinen Definition versteht man unter Human-Biomonitoring (HBM, s. a. Abkürzungsverzeichnis) die Untersuchung von human-biologischem Material zur Bestimmung von Gefahrstoffen oder deren Metaboliten bzw. von Effektparametern, um eine Belastung bzw. mögliche Gesundheitsgefährdung zu erfassen und zu bewerten. Am Arbeitsplatz können die Ergebnisse des HBM darüber hinaus wichtige Informationen liefern, um die Wirksamkeit von Maßnahmen des Arbeits- und Gesundheitsschutzes angemessen zu beurteilen (AfAMed 2015). Im Rahmen bevölkerungsbezogener HBM-Programme wird die Gefahrstoffbelastung durch umwelt- oder lebensstilbedingte Expositionen untersucht und es können zeitliche sowie geographische Trends identifiziert werden (z. B. Schwedler et al. 2019). Dazu bedarf es geeigneter Verfahren der chemischen Analytik, um die zumeist in nur geringen Konzentrationen vorliegenden Zielsubstanzen aus der biologischen Matrix zu extrahieren und anschließend spezifisch und empfindlich zu bestimmen.

Ein besonders geeignetes Verfahren zur effizienten Abtrennung flüchtiger Zielverbindungen von der biologischen Matrix und zur anschließenden sensitiven Bestimmung stellt die gaschromatographische Dampfraumanalyse, im Folgenden vereinfachend „Headspace-Analytik“ (oder Headspace-Gaschromatographie, Headspace‑GC, Headspace-Technik) genannt, dar. Die Headspace-Analytik ermöglicht die simultane Messung eines breiten Parameterspektrums innerhalb verschiedener Substanzgruppen, in der Regel ohne vorherige aufwändige Probenaufarbeitung oder Derivatisierung (Ikeda 1999).

Für die Headspace-Analytik wird das Probenmaterial in einem gasdicht verschlossenen Probengläschen je nach Anwendung auf eine Temperatur im Bereich von 40–80 °C erwärmt. Leichtflüchtige Verbindungen reichern sich dabei im Dampfraum („headspace“) über der flüssigen Probe an und werden so von der biologischen Matrix abgetrennt. Nach der Einstellung des Dampf-Flüssigkeits-Gleichgewichtes wird ein Aliquot der Gasphase entnommen und gaschromatographisch analysiert. Auf diese Weise können verschiedene organische Lösungsmittel, darunter aliphatische und aromatische Kohlenwasserstoffe, Halogenkohlenwasserstoffe, Alkohole, Ketone, Ether und Ester zumeist störungsfrei bestimmt werden. Im Gegensatz zur Injektion von flüssigen Probenextrakten erfolgt bei der Headspace-Analytik nur eine geringfügige Überführung von Matrixbestandteilen in das Chromatographiesystem und den Detektor. Grundsätzlich ermöglicht das hierdurch erzielte verringerte Hintergrundrauschen niedrige Bestimmungsgrenzen, so dass Analyten bis in den Hintergrundbereich der beruflich nicht belasteten Allgemeinbevölkerung detektierbar werden. Darüber hinaus ist die Belastung des gaschromatographischen Systems mit Matrixbestandteilen niedriger, so dass sich die Nutzungsdauer vor einer Reinigung oder Wartung erhöht.

Das Verfahren der Headspace-Analytik wurde Ende der 1950er bis Anfang der 1960er Jahre in den USA zur Analyse von Geschmacks-, Geruchs- und Aromastoffen entwickelt (Bassette et al. 1962; Buttery und Teranishi 1961; Mackay et al. 1961; Teranishi et al. 1962). Wenige Jahre später wurde es erstmals erfolgreich zur Bestimmung des Alkoholgehaltes in Blut angewandt (Machata 1964, 1967). Über die Quantifizierung von Ethanol hinaus wurde die Headspace-Analytik zunächst zur Bestimmung der Löslichkeit von Narkosemitteln (Butler et al. 1967; Fink und Morikawa 1970; Purchase 1963; Yamamura et al. 1966) sowie zur Bestimmung von Gasen (Curry et al. 1962; Dominguez et al. 1959; Galla und Ottenstein 1962; Hamilton 1962; Ramsey 1959), weiteren Alkoholen (Machata 1964) und Lösungsmittel (Goldbaum et al. 1964) eingesetzt. Seither hat sich die Headspace-Analytik mit verschiedenen Modifikationen in unterschiedlichen Forschungs- und Anwendungsbereichen etabliert und ist ein Standardverfahren in der forensischen Chemie, der klinischen Chemie, der Umweltchemie, der Lebensmittelchemie sowie der Polymerforschung geworden (Wang et al. 2008).

Entsprechend der vielfältigen Anwendungsbereiche gibt es eine umfangreiche Literatur, die sich mit den Grundlagen, der Methodenentwicklung und der Anwendung der Headspace-Analytik befasst. So sind Theorie und Praxis der „statischen“ Headspace-Analytik bei Hachenberg und Schmidt (1977), Ioffe und Vitenberg (1984) sowie bei Kolb und Ettre (2006) ausführlich dargestellt. Auch finden sich in den Lehrbüchern zur Gaschromatographie häufig Unterkapitel zu verschiedenen Headspace-Techniken (Grob und Barry 2004; McNair et al. 2019; Poole 2012). Darüber hinaus sind mehrere Übersichtsartikel zur Headspace-Analytik publiziert (siehe Literaturverweise in Kolb und Ettre 2006), wobei sich insbesondere die Arbeiten von Seto (1994) und von Mills und Walker (2000) mit der Bestimmung flüchtiger Substanzen in biologischen Proben befassen.

Explizit für das HBM im arbeitsmedizinischen Bereich wurden Headspace-Methoden in Deutschland seit 1977 auch von der Arbeitsgruppe „Analysen in biologischem Material“ der Ständigen Senatskommission zur Prüfung gesundheitsschädlicher Arbeitsstoffe (MAK‑Kommission) erarbeitet, geprüft und publiziert. Diese Methoden decken eine große Bandbreite industriell bedeutsamer Lösungsmittel ab. Neben insgesamt 24 in einer Sammelmethode zusammengefassten Parametern (Machata und Angerer 1983) wurden noch weitere Headspace-Methoden für spezifische Stoffgruppen publiziert, beispielsweise zur Bestimmung von Alkoholen und Ketonen (Angerer et al. 1996) und zur Bestimmung halogenierter Aliphaten (Angerer et al. 1991), halogenierter Aromaten (Lewalter et al. 1991) sowie der BTEX-Aromaten (Benzol, Toluol, Ethylbenzol und die isomeren Xylole) (Angerer et al. 1994).

Neuerungen in der instrumentellen Analytik ließen es sinnvoll erscheinen, die von der Kommission publizierten Analysenverfahren zu überarbeiten und zu aktualisieren. So wurden seit 2006 – und verstärkt seit 2017 – Verfahren zur Bestimmung flüchtiger Gefahrstoffe in der „MAK Collection online“ publiziert, bei denen die Headspace-GC in Verbindung mit einer massenspektrometrischen (MS) Detektion der Zielanalyten als besonders sensitives und spezifisches Verfahren genutzt wird. So folgten auf die Methode zur Bestimmung von Methylquecksilber in Blut (Hoppe und Heinrich-Ramm 2006) die Verfahren für Tetrahydrofuran (THF) in Urin (Blaszkewicz und Angerer 2012), Trichloressigsäure in Urin (Will et al. 2017), Methyl‐*tert*‐butylether (MTBE) in Blut und Urin (Hoppe et al. 2018), Aromaten im Blut (Göen et al. 2018), Aromaten in Urin (Van Pul et al. 2018), 1‑Brompropan und 2‑Brompropan in Urin (Roßbach et al. 2019), Alkohole, Ketone und Ether in Urin (Göen et al. 2020) sowie für chlorierte Kohlenwasserstoffe in Blut (Göen et al. 2021).

## 2 Grundlegende Prinzipien der Headspace-Technik

Im Folgenden sind die grundlegenden Prinzipien der Headspace-Technik kurz dargestellt. Zur weiteren Vertiefung der Thematik sei auf Kremser et al. (2016) verwiesen. Dort wurde auch ein systematischer Vergleich von statischen und dynamischen Headspace-Techniken durchgeführt und der Einfluss der jeweiligen Technik auf die Präzision und Nachweisgrenze bei der Bestimmung verschiedener Analyten untersucht.

### 2.1 Statische Headspace-Technik

In der statischen Headspace-Analytik wird die Gasphase einer (meist wässrigen) Probe untersucht, sobald sich das Phasengleichgewicht eingestellt hat. Hierfür wird die Probe in ein gasdicht verschlossenes Gefäß transferiert und für eine bestimmte Dauer auf eine vorgegebene Temperatur erwärmt. Die flüchtigen Komponenten der Probe verteilen sich dabei zwischen der Flüssigkeit und der Gasphase, bis sich ein Gleichgewicht zwischen beiden Phasen eingestellt hat (Penton 2010). Dann wird ein Volumenaliquot der Gasphase in einen Gaschromatographen injiziert. Alle Headspace-Techniken basieren auf diesem grundlegenden Prinzip.

**Abb. 1:**
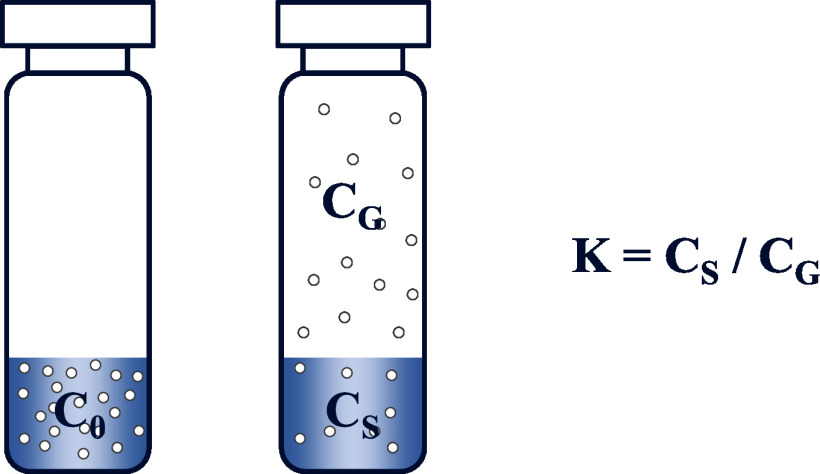
Verteilung einer flüchtigen Komponente in einem Headspace-Probengefäß (c_0_ = ursprüngliche Konzentration des Analyten in der Probe, c_s_ = Analytkonzentration in der Gasphase nach Gleichgewichtseinstellung, c_g_ =Analytkonzentration in der Gasphase nach Gleichgewichtseinstellung,  K = Verteilungskoeffizient)

Die abgeschlossene Gleichgewichtseinstellung zwischen den beiden Phasen ist eine zwingende Voraussetzung für zuverlässige und reproduzierbare Messungen (Sithersingh und Snow 2012). Daher erfolgt in der Regel eine Thermostatisierung der Proben für mindestens 30 min bei 40 °C (Blut) oder 60–80 °C (Plasma, Urin). Nach erfolgter Gleichgewichtseinstellung ist das Verhältnis der Analytkonzentration in der Probe und in der Gasphase konstant. Diese Konstante wird als Verteilungskoeffizient K bezeichnet (siehe Abbildung 1).

Es hängt stark von der chemischen Struktur der zu analysierenden Substanz ab, ob sie einer Headspace-Analytik zugänglich ist, da der Verteilungskoeffizient K eine substanzspezifische Größe ist. Ein niedriger Verteilungskoeffizient bedeutet eine hohe Analytkonzentration in der Gasphase im Vergleich zur wässrigen Phase (biologischen Matrix) und zeigt damit an, dass der jeweilige Analyt für eine Quantifizierung mittels Headspace-Analytik gut geeignet ist.

Der Verteilungskoeffizient K ist unter anderem abhängig von der Löslichkeit des Analyten in der biologischen Matrix. Eine geringe Löslichkeit führt zu einer höheren Analytkonzentration in der Gasphase und somit zu einem kleineren Verteilungskoeffizienten. Zur Beeinflussung der Löslichkeit können verschiedene Methoden, wie das Aussalzen oder eine pH‑Wert-Anpassung, angewandt werden (Penton 2010; Sithersingh und Snow 2012).

Da der Verteilungskoeffizient K außerdem mit steigender Temperatur abnimmt, ist eine möglichst hohe und konstante Thermostatisierungstemperatur für die Headspace-Analytik anzustreben. Im Fall von Blutproben ist die praktisch nutzbare Thermostatisierungstemperatur deutlich begrenzt, da die oberhalb von 40 °C einsetzende Koagulation die Einstellung eines Gleichgewichtes erschwert und zu höheren Verteilungskoeffizienten führt.

Grundsätzlich kann die Konzentration flüchtiger Stoffe im Dampfraum des Headspace-Probengefäßes über die Formel (Gleichung 1)


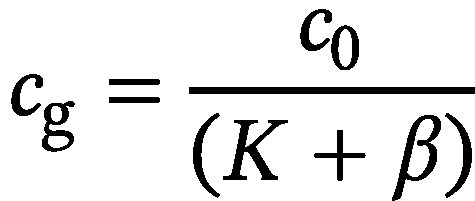
(1)

berechnet werden, wobei c_g_ die Konzentration des flüchtigen Analyten in der Gasphase darstellt und c_0_ die ursprüngliche Konzentration des Analyten in der Probe. Der Verteilungskoeffizient K gibt die Gleichgewichtsverteilung des Analyten zwischen flüssiger Probenphase und Gasphase an und das Phasenverhältnis β das Volumenverhältnis von Gasphase zu flüssiger Probenphase.

Je kleiner die Summe aus K und β wird, desto höher ist die Konzentration des Analyten in der Gasphase und damit auch die Sensitivität des Verfahrens. Eine Vergrößerung des Probenvolumens kann über ein geändertes Phasenverhältnis β zur Sensitivitätssteigerung beitragen, allerdings kommt dieser Effekt nur zum Tragen, wenn K sehr viel kleiner als β ist. Der Verteilungskoeffizient K wird mit steigender Temperatur generell kleiner (und damit die Konzentration im Dampfraum höher), wobei dieser Effekt umso größer ist, je besser sich der jeweilige Analyt im wässrigen Medium löst (Kolb und Ettre 2006).

Im Gegensatz zur statischen Headspace-Technik kann mit dynamischen Headspace-Verfahren, die auf einer mehrfachen Entnahme von Probenaliquoten aus der Gasphase beruhen, eine signifikant höhere Empfindlichkeit erreicht werden, so dass auch Analyten detektierbar werden, die nur in sehr geringen Konzentrationen vorliegen (vgl. Abschnitt 2.3).

### 2.2 Statische Headspace-Technik mit Anreicherung

Viele statische Headspace-Methoden verwenden anstelle der direkten Injektion aus dem Dampfraum ein Adsorptionsmittel oder eine Kühlfalle („cryogenic trap“), um die Analyten vor der Überführung in den Gaschromatographen aus der Gasphase anzureichern. Bei der Headspace-Solid Phase Micro Extraction (HS‑SPME) wird das Adsorptionsmittel direkt in das Probengefäß eingebracht (Mills und Walker 2000; Pragst 2007). Andere Anreicherungsmethoden sind die Stir-Bar Sorptive Extraction (SBSE) (David und Sandra 2007; Nazyropoulou und Samanidou 2015; Prieto et al. 2010) sowie die Single-Drop Micro Extraction (SDME) (Jeannot et al. 2010; Palit et al. 2005), die beide auf einem der SPME vergleichbaren Prinzip beruhen. Die HS‑SPME ist dabei die am weitesten verbreitete Technik (Demeestere et al. 2007; Jochmann et al. 2006; Laaks et al. 2012; Nerín et al. 2009).

#### 2.2.1 Solid Phase Micro Extraction (SPME)

**Abb. 2:**
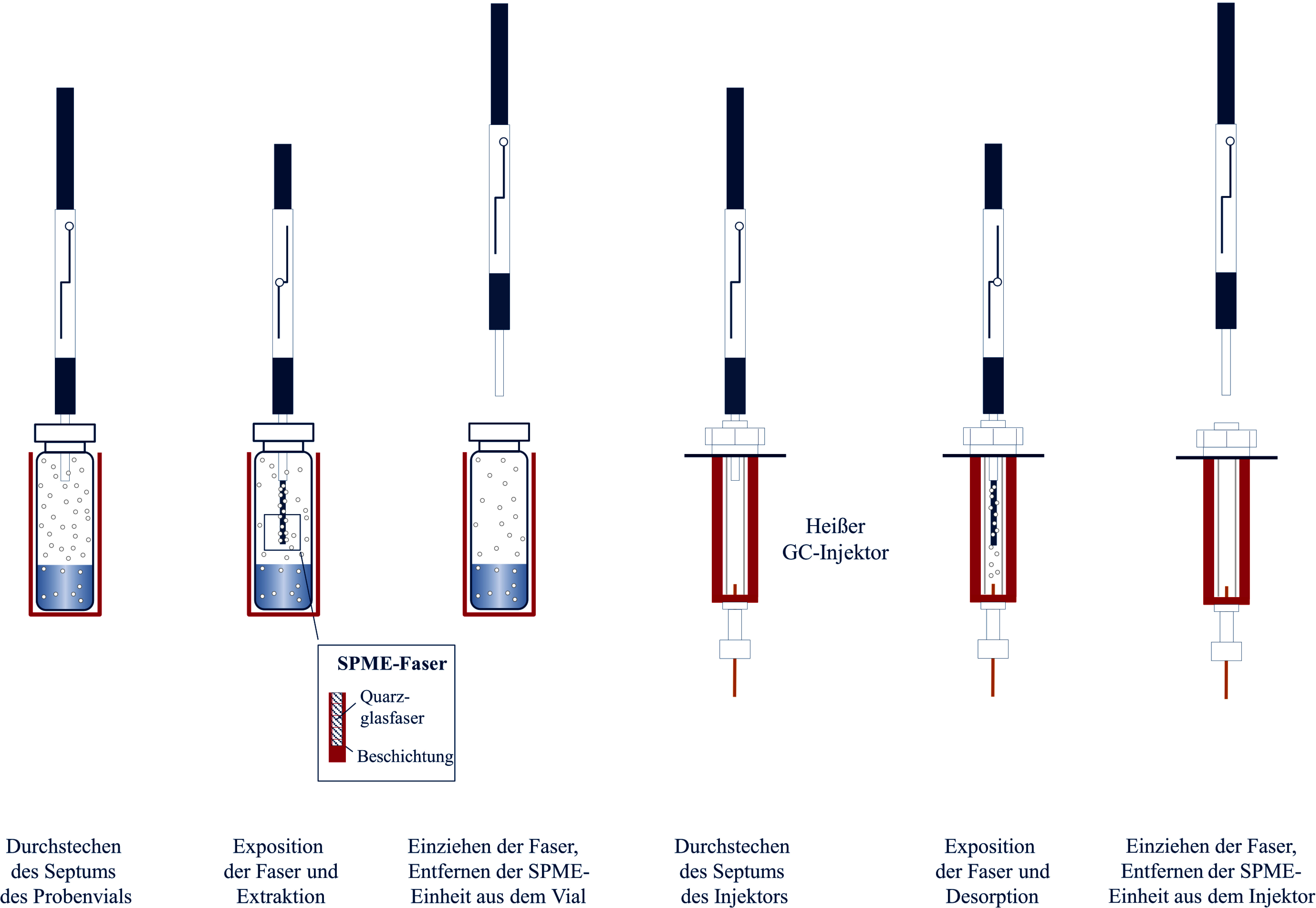
Grundlegende Arbeitsschritte der Headspace-SPME-Technik

Bei der SPME handelt es sich um eine lösungsmittelfreie Extraktionstechnik, bei der eine Kanüle mit den Dimensionen einer üblichen GC-Injektionsspritze mit innen geführter Kunststofffaser durch das Septum in die Gasphase eines Probengefäßes eingebracht wird. Die SPME-Faser wird danach aus der Kanüle in den Gasraum des Probengefäßes geschoben, verbleibt dort für eine vorgewählte Zeit und wird anschließend wieder in die Kanüle zurückgeführt. Die SPME-Faser ist mit einer an die Zielanalyten angepassten stationären Phase (z. B. Tenax^®^, Silicagel, Aktivkohle) beschichtet, an der die Sorption der Zielanalyten während der vorgewählten Zeit stattfindet (Baltussen et al. 2002; Mills und Walker 2000). Im Gesamtsystem stellt sich daher ein zweites Gleichgewicht zwischen der Gasphase und dem Sorbens der SPME-Faser ein. Durch gezielte Beeinflussung der Verteilungskoeffizienten beider Gleichgewichte kann im Vergleich zur normalen statischen Headspace-Technik eine deutlich bessere Empfindlichkeit erreicht werden (Sithersingh und Snow 2012). Nach erfolgter Einstellung des Sorptionsgleichgewichtes oder nach Ablauf einer definierten Zeitspanne wird die Kanüle mit der wieder in die Kanüle eingezogenen SPME-Faser in den heißen Injektionsport des Gaschromatographen eingebracht. Dort wird die Faser erneut herausgeschoben und die Analyten werden durch Thermodesorption von der Sorptionsphase freigesetzt und anschließend analysiert. Abbildung 2 zeigt die grundlegenden Arbeitsschritte der Headspace-SPME-Technik.

Die benötigte Extraktionszeit ist dabei unabhängig von der Konzentration der Analyten in der Probe (Vas und Vékey 2004). Eine schnellere Äquilibrierung kann beispielsweise durch Rühren oder Schütteln der Probe erzielt werden. Typische SPME-Fasern können für etwa 100 Analysen verwendet werden (Pragst 2007). Die besonderen Vorteile der SPME-Headspace-Analytik liegen in ihrer relativ einfachen Durchführbarkeit sowie in den vergleichsweise geringen Analysenkosten. Mit der SPME-Analytik werden sehr saubere und konzentrierte Probenextrakte erhalten, die sehr gut für eine sich anschließende hochempfindliche und selektive Analyse, z. B. mittels Massenspektrometrie, geeignet sind (Nerín et al. 2009; Vas und Vékey 2004).

Da es sich bei der Sorption um einen kompetitiven und matrixabhängigen Prozess handelt, kann die Verwendung von internen Standards (ISTDs) für quantitative Messungen mittels Headspace-SPME erforderlich sein. Dabei sind ISTDs zu bevorzugen, die den Zielanalyten strukturell und chemisch möglichst ähnlich sind (Pragst 2007). Allerdings können sich auch bei der Verwendung von isotopenmarkierten ISTDs nichtlineare Kalibrierkurven ergeben (Pragst 2007) (siehe Abschnitt 4.4).

Ebenso wie bei der statischen Headspace-Technik ist es auch für eine zuverlässige SPME-Analytik zwingend erforderlich, dass die Analysebedingungen (Probenzusammensetzung, Temperatur, Probenmenge und Headspace-Volumen) während der Probenäquilibrierung konstant gehalten werden. Eine höhere Anreichung der Analyten an der SPME-Faser kann durch zusätzliche Kühlung der SPME-Faser erreicht werden (Ghiasvand et al. 2016; Pragst 2007).

Die Nachteile der SPME-Technik sind vor allem die mechanische Empfindlichkeit der Fasern sowie die limitierte Auswahl an stationären Phasen. Daneben ist auch die begrenzte Anreicherungskapazität aufgrund des vergleichsweise geringen Volumens der Sorptionsphase sowie die relativ kurze Lebensdauer der Fasern nachteilig (Jochmann et al. 2008; Laaks et al. 2010, 2012; Nerín et al. 2009). Weiterentwickelte SPME-Fasersysteme wurden dementsprechend optimiert. Während konventionelle SPME-Fasern ein Sorbensvolumen von nur ca. 0,6 μl aufweisen, stehen bei SPME-Fasern mit größeren Oberflächen bis zu 15 μl zur Anreicherung zur Verfügung. Gleichzeitig sorgen Veränderungen am Design (Extraktionsphase mit Edelstahlkern, spitze Frontpartie zur besseren Septumpenetration) für eine erhöhte mechanische Stabilität der Extraktionseinheit (Kremser et al. 2016).

#### 2.2.2 Stir-Bar Sorptive Extraction (SBSE) / Headspace Sorptive Extraction (HSSE)

Die Einführung der Stir-Bar Sorptive Extraction (SBSE)-Technik im Jahr 1999 zielte darauf ab, Nachteile der bisher vorhandenen Anreicherungstechniken zu vermeiden. Hierzu zählte unter anderem die geringe Anreicherungskapazität der SPME-Verfahren aufgrund kleiner Sorbensvolumina (Baltussen et al. 1999). Die SBSE-Technik wurde ursprünglich für die Aufkonzentrierung flüchtiger und semiflüchtiger Verbindungen aus wässrigen Proben entwickelt. Kurze Zeit später wurden unter dem Namen Headspace Sorptive Extraction (HSSE) schon Headspace-Anwendungen dieser Technik publiziert (Bicchi et al. 2000; Tienpont et al. 2000). Bei der SBSE und HSSE erfolgt die Anreicherung der Analyten in einer vergleichsweise dicken Schicht eines Sorbens, die auf ein glasummanteltes magnetisches Rührstäbchen aufgebracht ist. Abhängig von der Länge des Rührstäbchens liegen die Sorbensvolumina zwischen 25 und 250 μl. Diese Volumina sind damit um zwei bis drei Größenordnungen größer als bei der SPME zur Verfügung stehende Volumina. Bei der HSSE erfolgt eine statische Headspace-Anreicherung, indem das Rührstäbchen für eine festgelegte Zeit in den Dampfraum der thermostatisierten Probe eingeführt wird. Anschließend wird das Stäbchen in einem Glasröhrchen in ein Thermodesorptionssystem überführt. An die thermische Freisetzung der Analyten aus dem Sorbensmaterial schließt sich die Analyse z. B. mittels GC-MS an. Aufgrund des höheren Sorbensvolumens können sich im Vergleich zur SPME verlängerte Desorptionszeiten von bis zu 15 min ergeben. Mit Hilfe einer Kryofokussierung vor der chromatographischen Trennung wird auch unter diesen Bedingungen eine quantitative und fokussierte Überführung der Probenbestandteile in das Chromatographiesystem sichergestellt (Prieto et al. 2010). Als Vorteile der SBSE- bzw. HSSE-Technik werden deren Automatisierbarkeit und Flexibilität (mit der Möglichkeit der Anreicherung sowohl aus der flüssigen als auch aus der Gasphase) gesehen. Das hohe Sorbensvolumen ermöglicht, insbesondere bei Nutzung im Dampfraum und damit unter Umgehung einer möglichen Sorption schwer flüchtiger Probenbestandteile, eine sensitive und gleichzeitig robuste Analytik mit guter Reproduzierbarkeit (Cordero et al. 2009). Die Auswahl der verfügbaren Sorptionsphasen beschränkte sich lange Zeit auf das unpolare Polydimethylsiloxan (PDMS). SBSE- bzw. HSSE-Verfahren kamen daher überwiegend bei mittel- bis hochflüchtigen Verbindungen zur Anwendung, die zugleich ausreichend thermisch stabil sein mussten. Zwischenzeitlich ist neben reinem PDMS auch ein PDMS/Ethylenglykol–Copolymer als Anreicherungsphase kommerziell verfügbar (GERSTEL GmbH & Co. KG 2025). Daneben finden sich in der wissenschaftlichen Literatur zahlreiche weitere Ansätze zur Entwicklung alternativer Anreicherungsphasen für die SBSE/HSSE (Nazyropoulou und Samanidou 2015). Zusammen mit den vergleichsweise hohen Kosten für die erforderliche Ausrüstung sorgte die beschränkte Auswahl an Sorptionsphasen in der Vergangenheit insgesamt jedoch für eine geringere Verbreitung dieser Technik, etwa im Vergleich zur SPME (Paiva et al. 2021).

#### 2.2.3 Single-Drop Micro Extraction (SDME)

Die Single-Drop Micro Extraktion (SDME) stellt seit etwa Mitte der 1990er Jahre eine vergleichsweise einfache und leicht zu implementierende Mikromethode zur Extraktion von Zielanalyten aus einer Matrix bzw. dem Dampfraum über einer Probe dar. Im Probengläschen wird hierbei – in der Regel unter Verwendung einer Chromatographiespritze – ein (an der Kanüle) hängender Tropfen eines Extraktionslösungsmittels erzeugt. Der Tropfen wird für eine vorgegebene Zeit in die zu untersuchende Lösung eingebracht oder verbleibt bei Headspace-Anwendungen im Dampfraum der Probe. Nach Sorption der Analyten in das Lösungsmittel wird der nur wenige Mikroliter umfassende Tropfen in die Kanüle der Spritze zurückgesaugt und anschließend in den GC überführt, wo die Probenkomponenten aufgetrennt und anschließend quantifiziert werden (Afshar Mogaddam et al. 2019; Jeannot et al. 2010).

In der Headspace-SDME (Przyjazny und Kokosa 2002; Tankeviciute et al. 2001; Theis et al. 2001) werden zur Extraktion üblicherweise hochsiedende Lösungsmittel wie 1‑Octanol oder langkettige *n*‑Alkane (z. B. *n*‑Hexadecan) genutzt. Generell ist jedoch eine vergleichsweise große Palette an Sorptionsmitteln unterschiedlichster Polarität denkbar (z. B. *N*‑Methylpyrrolidon, Ethylenglykole oder Diethylphthalat) (Jeannot et al. 2010; Wood et al. 2004). Als limitierend stellt sich oftmals die Stabilität des Tropfens heraus, die stark vom verwendeten Lösungsmittel abhängt. Ungünstig wirken sich hierbei neben einer hohen Flüchtigkeit auch eine geringe Viskosität und Oberflächenspannung aus (Kissoudi und Samanidou 2018). Neben klassischen organischen Lösungsmitteln können insbesondere für polare Analyten auch ionische Flüssigkeiten oder Wasser bzw. wässrige Lösungen als Extraktionsphasen eingesetzt werden (Afshar Mogaddam et al. 2019; Jeannot et al. 2010; Kissoudi und Samanidou 2018). Der Ablauf einer HS‑SDME-Analyse ist dem einer HS‑SPME-Analyse vergleichbar, ohne dass hierfür spezielle zusätzliche Ausrüstung benötigt wird. Entsprechende Analysen können daher sowohl manuell aber auch sehr gut automatisiert durchgeführt werden (Wood et al. 2004). Die Trennung und Quantifizierung der Analyten erfolgt dabei überwiegend mittels Gaschromatographie und seltener mittels Flüssigkeitschromatographie (Jeannot et al. 2010).

### 2.3 Dynamische Headspace-Techniken

#### 2.3.1 Purge & Trap

**Abb. 3:**
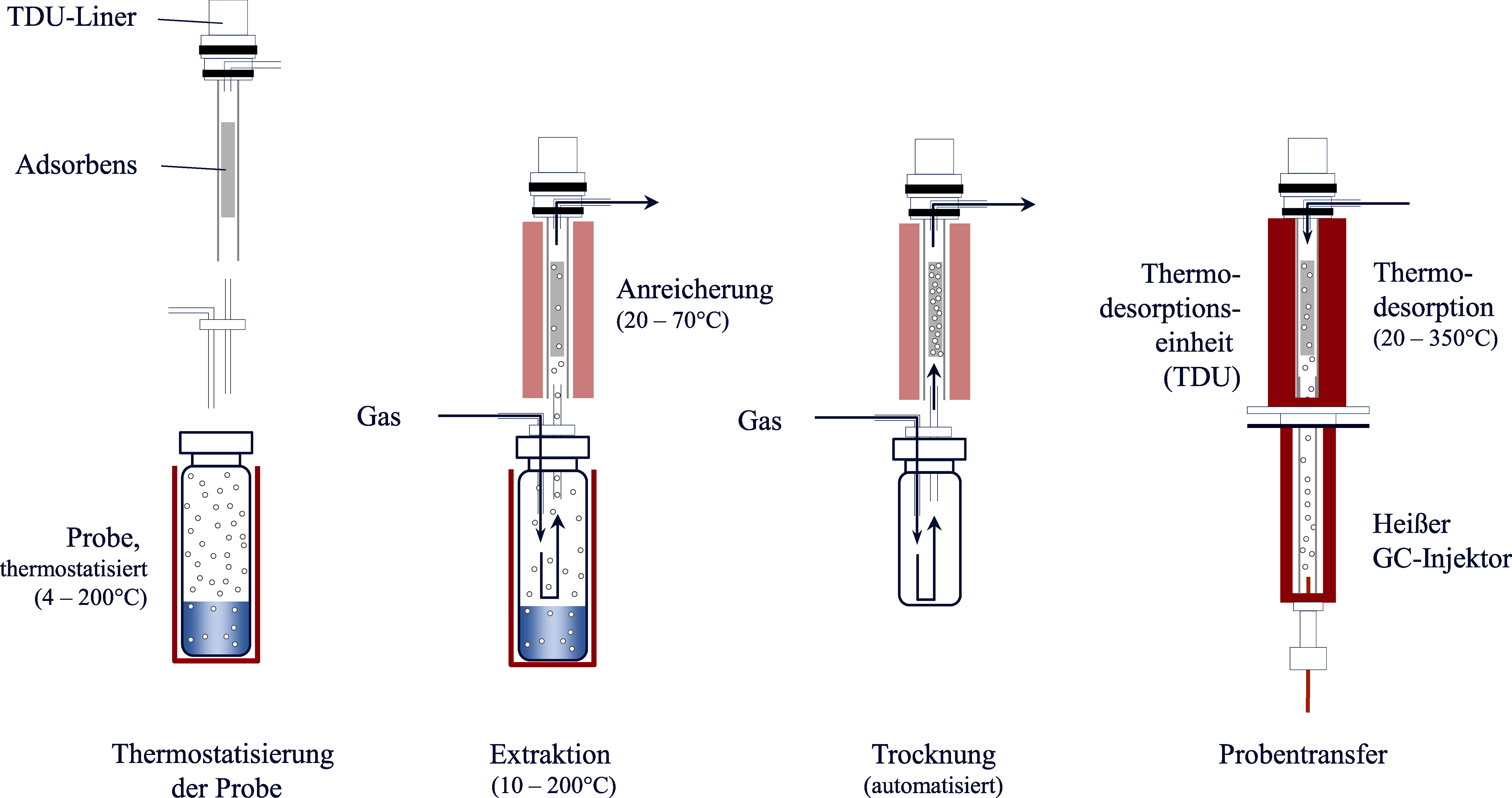
Grundlegende Arbeitsschritte bei der Purge & Trap-Technik

Die Purge & Trap-Technik zählt zu den dynamischen Headspace-Methoden. Hierbei wird ein Inertgas durch die wässrige Probe geleitet und befördert die flüchtigen Analyten in die Gasphase. Im Gegensatz zu statischen Headspace-Methoden wird hier keine Gleichgewichtseinstellung erreicht, da der Gasstrom kontinuierlich Analyten aus der wässrigen Probe austreibt. Durch die Ausleitung des Gasstromes aus dem Probengefäß und das kontinuierliche Einleiten von Inertgas in die Probe werden die Analyten weitgehend vollständig in die Gasphase überführt (Sithersingh und Snow 2012). Zur Anreicherung der Analyten wird der Gasstrom in eine Kühlfalle („cryogenic trap“) geleitet, in der die Zielanalyten bei niedriger Temperatur kondensieren und/oder durch Sorption (Adsorption an einer Oberfläche, Absorption in einer flüssigen Phase) lokal angereichert werden. Nach beendetem Extraktionsschritt erfolgt die Desorption der Analyten analog zur SPME‑Technik durch Thermodesorption im GC‑Injektor (Abbildung 3).

Durch die kontinuierliche Extraktion der flüchtigen Analyten aus der Matrix können mit dieser Technik im Vergleich zur statischen Headspace-Analyse deutlich niedrigere Nachweisgrenzen erreicht werden. Sofern eine Sorbensfalle genutzt wird, stellt die große Auswahl an Sorptionsmaterialien einen weiteren Vorteil dar. Für Multimethoden können beispielsweise auch mehrschichtige Sorbentien eingesetzt werden (z. B. aus Tenax^®^, Silicagel, Aktivkohle), die eine große Bandbreite an Analyten binden (Sithersingh und Snow 2012).

Ein Nachteil dieser Technik liegt in der Kontaminationsgefahr. Da das Inertgas die wässrige Probe durchströmt, enthält der ausgeleitete Gasstrom auch geringe Mengen an Wasser, das die anschließende Analytik stören kann. Dem wird zum Teil mit nachgeschalteten Trocknungsschritten begegnet (Abbildung 3). Da die Analyten zudem relativ lange Wege bis zum Injektor zurückzulegen haben, ist die Gefahr einer Kontamination, einer Adsorption oder einer Kondensation an kühleren Oberflächen sowie von Peakverbreiterungen in der sich anschließenden Chromatographie generell groß. Aufgrund möglicher Schaumbildung durch den Inertgasstrom ist diese Technik für biologische Materialien, insbesondere für Blut, nur bedingt anwendbar. Alternativ kann der Gasstrom auch an der Probenoberfläche entlanggeführt werden (Demeestere et al. 2007), was zwar die Anreicherungsrate verringert, aber für wasserdampfarme Analytenextrakte sorgt. Der Zeitaufwand für die Purge & Trap-Technik ist gegenüber anderen Methoden relativ hoch (Demeestere et al. 2007).

#### 2.3.2 In-tube Extraction (ITEX)

Die In-tube Extraktionstechnik (ITEX) ist eine neuere, lösungsmittelfreie Anreicherungsmethode. Hier erfolgt die Anreicherung direkt in der Headspace-Spritze, wobei das als Feststoff vorliegende Adsorptionsmaterial (i. d. R. Tenax TA) im oberen Teil der Kanüle fixiert ist. Um die Kanüle herum befindet sich ein Heizmantel, der eine optimale Thermodesorption der Analyten zur Überführung in den GC-Injektor gewährleistet.

**Abb. 4:**
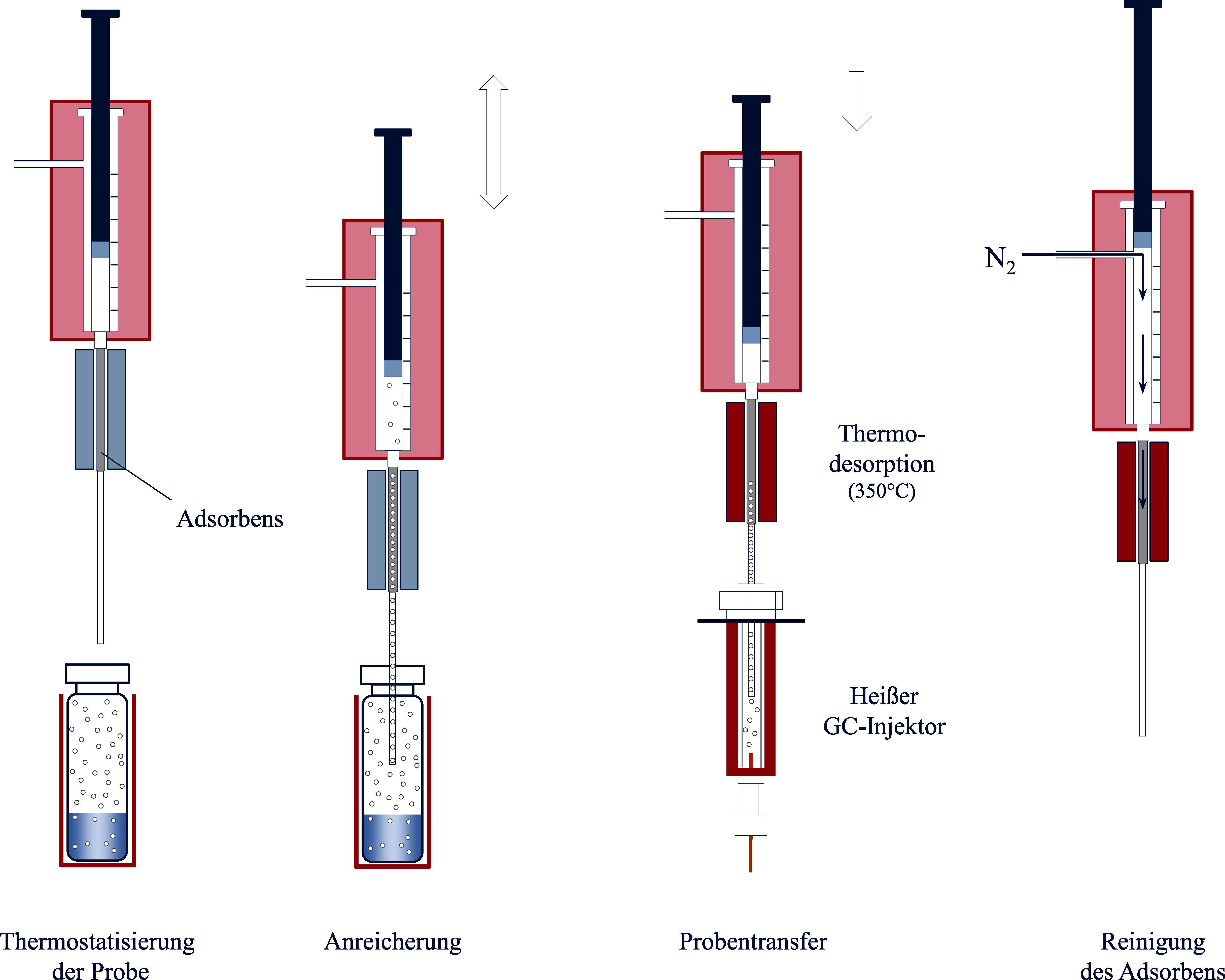
Grundlegende Arbeitsschritte der ITEX-Technik

Ebenso wie bei anderen Headspace-Techniken wird die zu untersuchende Probe zunächst unter definierten Bedingungen thermostatisiert und eventuell gerührt oder geschüttelt. Anschließend durchsticht die Kanüle das Septum des Probengefäßes und die Gasphase wird mehrfach in die Kanüle aufgezogen, wodurch der Analyt über das Adsorptionsmaterial geleitet und dort festgehalten wird. Die Kanüle wird dann in den GC-Injektor eingeführt und der Analyt nach thermaler Desorption direkt analysiert. Nach der Desorption wird das Adsorptionsmaterial gereinigt, indem die heiße Kanüle mit einem Inertgas gespült wird. Abbildung 4 zeigt die grundlegenden Arbeitsschritte der ITEX-Technik.

Die ITEX-Technik hat den Vorteil, dass Probenaufbereitung und Anreicherung in einem Schritt erfolgen und die Durchführung vollständig automatisiert ablaufen kann. Zudem ist die Kontaminationsgefahr deutlich verringert (Laaks et al. 2010).

Die Hauptvorteile der ITEX-Technik im Vergleich zur SPME sind die deutlich höhere Adsorptionskapazität, die höhere mechanische Stabilität und die schnellere Anreicherung der Analyten durch das aktive Aufziehen der Gasphase (Jochmann et al. 2008; Laaks et al. 2010; Nerín et al. 2009). Zudem weist die ITEX-Spritze eine längere Lebensdauer auf und kann für bis zu 1000 Extraktionen verwendet werden. Durch den externen Heizmantel der Kanüle ist die Thermodesorption unabhängig von der Temperatur des GC‑Injektors (Jochmann et al. 2008; Rasanen et al. 2010). So können mit dieser Technik in der Regel deutlich niedrigere Nachweisgrenzen erreicht werden und eine Vielzahl von Analyten auch unterhalb arbeitsmedizinisch relevanter Konzentrationen erfasst werden (Laaks et al. 2015; Rasanen et al. 2010). Ein besonderer Vorteil gegenüber der SPME-Technik (s.o.) und auch der SPDE‑Technik (s. u.) ist die Vielseitigkeit der ITEX-Technik: das Adsorptionsmaterial liegt gepackt vor und kann daher aus einer größeren Anzahl von Materialien gewählt werden (Laaks et al. 2012).

Neben den bereits von der statischen Headspace-Analytik bekannten Einflussgrößen wird die Anreicherung mit dieser dynamischen Technik maßgeblich durch die Wahl des Adsorbens sowie durch die Hubzahl (und damit der Zahl der Extraktionszyklen) beeinflusst (Laaks et al. 2010, 2015). Analog zur SPME-Technik kann durch eine Kühlung der Kanüle eine verbesserte Anreicherung der Analyten erreicht werden (Laaks et al. 2015).

#### 2.3.3 Solid Phase Dynamic Extraction (SPDE)

Das Prinzip der Solid Phase Dynamic Extraction (SPDE)-Technik ist weitestgehend analog zur ITEX-Anreicherung und wurde als Verbesserung der SPME-Technik entwickelt (Lipinski 2000, 2001).

**Abb. 5:**
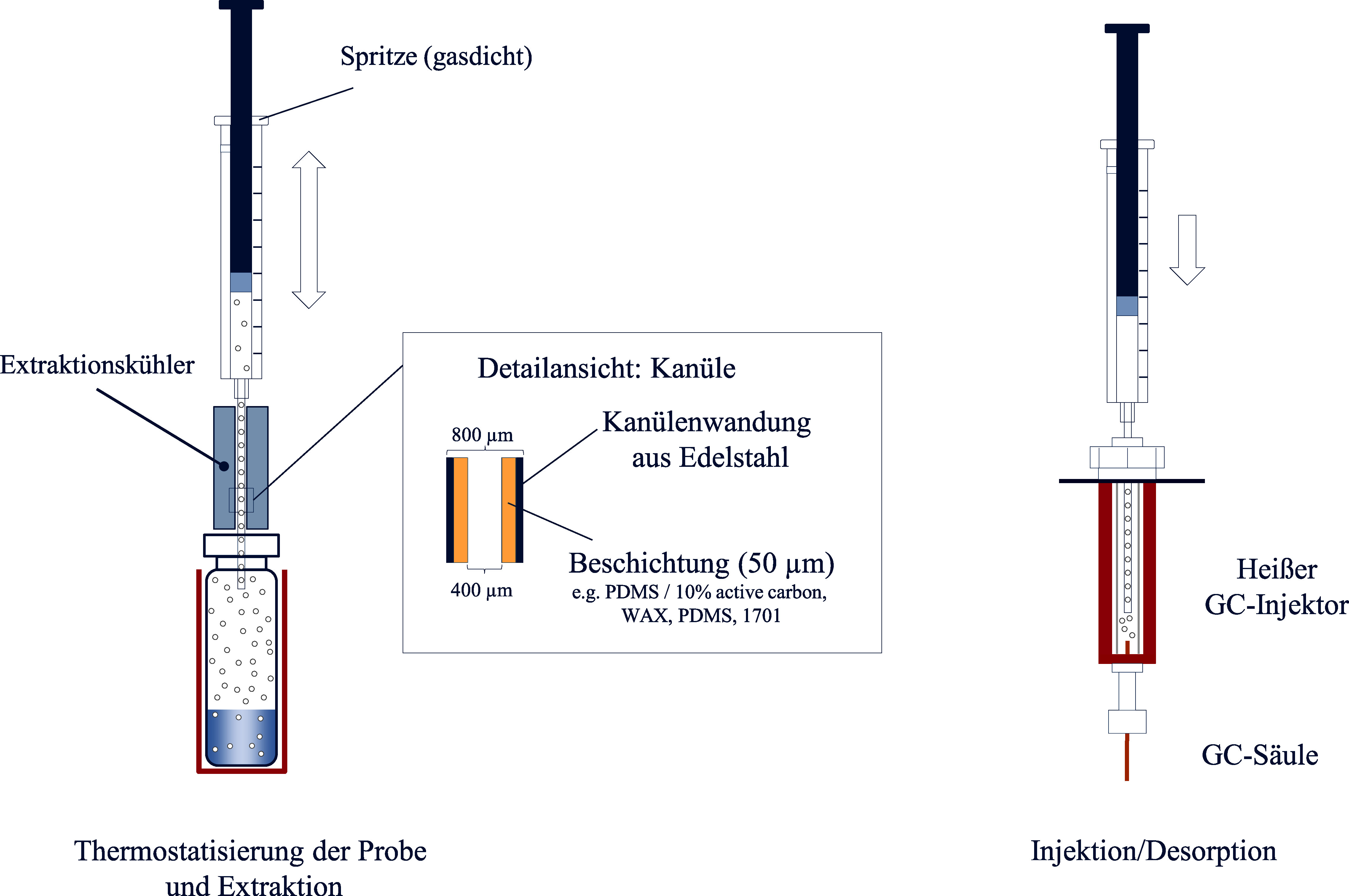
Arbeitsprinzip der SPDE-Technik

Abweichend von der ITEX-Methode befindet sich das Sorptionsmaterial jedoch nicht gepackt in der Injektionsspritze, sondern als Beschichtung auf der inneren Kanülenwand. Auch hier wird die Kanüle durch das Septum in das Probengefäß eingebracht und die Extraktion erfolgt dynamisch durch mehrmaliges Aufziehen der Spritze. Der Analyt wird dann im GC‑Injektor durch Thermodesorption wieder freigesetzt und analysiert (Nerín et al. 2009) (Abbildung 5). Auch bei der SPDE‑Technik kann die Anreicherung der Analyten durch Kühlung der Kanüle verbessert werden (Jochmann et al. 2006).

Die Vorteile dieser Technik entsprechen denen der ITEX-Technik (vgl. Abschnitt 2.3.2) und liegen vorrangig in der verbesserten Nachweisempfindlichkeit, wodurch sich diese Anreicherungstechnik auch zum Nachweis von polaren flüchtigen Substanzen im Spurenbereich eignet (Jochmann et al. 2006). Vorteilhaft ist zudem die Anpassung der Extraktionseffizienz durch Modifikation der Hubzahl (Nerín et al. 2009) sowie die generell gute Automatisierbarkeit (Laaks et al. 2012). Nachteilig ist die bislang relativ geringe Auswahl an stationären Phasen zur Analytenanreicherung (Laaks et al. 2012).

## 3 Headspace-Analytik im Human-Biomonitoring

### 3.1 Biologisches Material

Eine wichtige Voraussetzung für das Human-Biomonitoring ist die fachgerechte Gewinnung und Aufarbeitung eines geeigneten biologischen Untersuchungsmaterials, in dem –im Falle eines Belastungsmonitorings– die Konzentration des Gefahrstoffs oder des jeweiligen Metaboliten die Gesamtbelastung des Organismus repräsentiert. Aktuell werden zur quantitativen Bestimmung einer Gefahrstoffbelastung im arbeitsmedizinischen Kontext vorzugsweise Blut, Plasma, Serum, Erythrozyten sowie Urin herangezogen, da für diese Matrices zumeist eine enge Korrelation zwischen der Exposition am Arbeitsplatz und den jeweiligen Biomarker-Konzentrationen vorliegt. Ein weiterer Vorteil von Blut und Urin als Untersuchungsmaterialien besteht darin, dass diese standardisiert gewonnen werden können und bei arbeits- oder umweltmedizinischen Fragestellungen auch unter Routinebedingungen leicht zugänglich sind: ihre Gewinnung ist für die Betroffenen zumutbar und das Material in hinreichender Menge verfügbar (Alves et al. 2014; Angerer et al. 2007).

Dementsprechend sind die bislang für das Human-Biomonitoring von der Kommission publizierten Headspace-Verfahren für die Matrices Blut und Urin ausgelegt und validiert (siehe Abschnitt 5.1). Ob die jeweiligen Parameter in Blut oder Urin bestimmt werden, hängt von der Aufnahme- und Eliminationskinetik sowie dem Metabolismus der jeweiligen Arbeitsstoffe ab. Zudem muss die Kontaminationsproblematik berücksichtigt werden, die vor allem immer dann besteht, wenn nicht-metabolisierte Gefahrstoffe quantifiziert werden (siehe Abschnitt 4.1). Auch die arbeitsmedizinischen Beurteilungswerte für das Human-Biomonitoring (z. B. Biologischer Arbeitsstoff‑Toleranzwert (BAT), Biologischer Leitwert (BLW), Biological Limit Value (BLV) und Biological Exposure Index (BEI^®^)), sind fast ausschließlich für Blut und Urin abgeleitet (ACGIH 2025; DFG 2025; RAC 2025).

In der wissenschaftlichen Literatur sind weitere biologische Matrices beschrieben, die verwendet werden können, um flüchtige Substanzen mittels Headspace‑GC zu quantifizieren. Allerdings richtet sich das Interesse dann zumeist nicht auf arbeitsmedizinische, sondern auf umweltmedizinische, rechtsmedizinische oder toxikokinetische Fragestellungen. In diesen Arbeitsfeldern werden neben Blut und Urin auch Muttermilch, Faeces, Speichel, Liquor, Gewebehomogenate und weitere biologische Matrices untersucht (Mills und Walker 2000; Seto 1994).

### 3.2 Analyten und Stoffgruppen

Im Human-Biomonitoring wurde die Headspace-Analytik ursprünglich zur Bestimmung sehr leichtflüchtiger Verbindungen (VOCs = „volatile organic compounds“), die in relativ hohen Konzentrationen vorlagen, eingesetzt. Betrachtet man die physikochemischen Eigenschaften dieser Verbindungen, so lassen sich die VOCs entsprechend der Richtlinie 1999/13/EG des Rates der Europäischen Union (Europäischer Rat 1999) als Substanzen definieren, die bei 20 °C einen Dampfdruck von mindestens 10 Pa besitzen. Unter diese Definition, die auch von der International Union of Pure and Applied Chemistry (IUPAC) übernommen wurde (Duffus et al. 2007), fällt ein großes Substanzspektrum, das aliphatische und aromatische Kohlenwasserstoffe sowie sauerstoff-, stickstoff-, schwefel- und halogenhaltige Verbindungen einschließt (Hunter und Oyama 2000).

Neben diesen aufgrund ihrer stoffimmanenten physikochemischen Eigenschaften flüchtigen Substanzen sind der Headspace-Analytik grundsätzlich auch diejenigen Verbindungen zugänglich, die durch Derivatisierung, chemische oder thermische Umsetzung oder durch eine anderweitige Probenaufarbeitung in flüchtige Substanzen umgewandelt werden können. Beispiele hierfür sind die Derivatisierung der Trifluoressigsäure (Dallmeier und Müller 1982), die Proteinadduktspaltung von Aldehyden in Serum (Silva et al. 2018), die thermische Umsetzung von *N*‑Hydroxymethyl-*N*‑methylformamid (HNMF) zum *N*‑Methylformamid (Fernandes Knupp et al. 2005) sowie die thermische Zersetzung von Trichloressigsäure zu Chloroform (Angerer und Eben 1980) bzw. von Ameisensäure zu Kohlenmonoxid (Angerer und Schaller 1980).

Die heute zugängliche nachweisstarke Analysentechnik ermöglicht mittlerweile auch den Stoffnachweis im Ultraspurenbereich (Imbriani und Ghittori 2005), wobei sowohl weniger flüchtige Substanzen nachgewiesen werden können als auch solche, die nur in geringer Konzentration auftreten (Fantuzzi et al. 2001; Imbriani und Ghittori 2005; Takeuchi et al. 2002). Für den Bereich der Ultraspurenanalyse gibt es keine strenge Definition, er wird in der Literatur meist für Massenanteile von weniger als 10^–6 ^bis 10^–8^ g/g (1 ppm bis 10 ppb) verwendet (Brown und Milton 2005). So wurden auch von der Kommission in den letzten Jahren Headspace-Methoden für die Bestimmung unveränderter Aromaten (Van Pul et al. 2018) sowie von halogenierten Kohlenwasserstoffen (Roßbach et al. 2019) im Urin publiziert, die nur zu einem geringen Anteil mit dem Urin ausgeschieden werden.

Um Expositionen am Arbeitsplatz effektiv mittels Human-Biomonitoring erfassen und überwachen zu können, wurden Methoden für einzelne Analyten oder Sammelmethoden, in denen die flüchtigen Verbindungen aufgrund struktureller Gemeinsamkeiten zusammengefasst werden, entwickelt. Entsprechend wurden von der Kommission Methoden für die gemeinsame Erfassung der BTEX‑Aromaten in Blut (Angerer et al. 1994; Knecht und Angerer 1983) oder allgemeiner für die Erfassung von Aromaten in Blut oder Urin (Göen et al. 2018; Van Pul et al. 2018) erarbeitet und publiziert. Andere Methoden fassen die Bestimmung von Alkoholen, Ketonen und Ethern in Urin (Angerer et al. 1996; Göen et al. 2020) oder von halogenierten Kohlenwasserstoffen in Blut (Angerer et al. 1991; Göen et al. 2021) oder in Urin (Roßbach et al. 2019) zusammen.

Die Tatsache, dass anfänglich Analysenverfahren für unpolare Kohlenwasserstoffe in Blut bzw. in Blutkompartimenten und für polare Kohlenwasserstoffe in Urin entwickelt wurden, hat nicht nur dem Lösungsverhalten in den einzelnen biologischen Matrices Rechnung getragen, sondern auch den physiologischen Prozessen, da über den Urin vornehmlich polare Substanzen bzw. polare Metaboliten ausgeschieden werden. Folgerichtig sind die Beurteilungswerte (siehe Abschnitt 5.2) für diese Parameter zunächst auch nur für die entsprechenden Matrices abgeleitet worden.

Auch die Halbwertszeit der jeweiligen Substanzen in Blut und Urin beeinflusst die Auswahl der Matrix für die Bestimmung einzelner Biomonitoring-Parameter. Im Blut vorliegende leichtflüchtige Substanzen werden vornehmlich über die Lunge abgeatmet, was dazu führt, dass diese nach der Exposition sehr rasch eliminiert werden (siehe Tabelle 1). Für diese Parameter wurde von der Kommission der Probenahmezeitpunkt „unmittelbar nach Exposition“ in der MAK- und BAT‑Werte-Liste eingeführt (DFG 2025). Dieser Probenahmezeitpunkt gilt zurzeit für das arbeitsmedizinische Biomonitoring von 1,2‑Dichlorbenzol, Dichlormethan und Toluol in Blut. Ungeachtet dieses Hinweises zum Probenahmezeitpunkt stellt die zeitlich korrekte Probenahme für Gefahrstoffe mit kurzer Halbwertszeit eine große Herausforderung in der arbeitsmedizinischen Praxis dar. Aus diesem Grund wurden die Beurteilungswerte für Benzol, Toluol und die Xylol-Isomeren in Blut von der Kommission ausgesetzt und neue Beurteilungswerte in Urin abgeleitet (DFG 2025).

Stoffe, die mit dem Urin ausgeschieden werden, haben in der Regel längere Halbwertszeiten als leichtflüchtige Gefahrstoffe im Blut (siehe Tabelle 1). Dies gilt vor allem für die Metaboliten der Gefahrstoffe, zum Teil aber auch für die unveränderten Gefahrstoffe im Urin.

### 3.3 Detektoren

In Kombination mit der Headspace-Gaschromatographie werden verschiedene Detektoren genutzt (Angerer und Schaller 1976). In der Anfangszeit der Headspace-Gaschromatographie waren dies vor allem der Flammenionisationsdetektor (FID) und der Elektroneneinfangdetektor (ECD). Der FID ist ein sehr universeller Detektor, der kohlenstoffhaltige Verbindungen sensitiv erfasst und einen weiten linearen Arbeitsbereich über sechs Größenordnungen aufweist. Der ECD zählt zu den selektiven Detektoren, da er vor allem Verbindungen mit hoher Elektronenaffinität anzeigt. Insbesondere halogenierte und nitrierte Substanzen werden sensitiv gemessen, während andere stickstoff- und sauerstoffhaltige Verbindungen mit geringerer Empfindlichkeit erfasst werden. Hinsichtlich der Nachweisgrenzen übertrifft der ECD den FID für diese Analyten um mehrere Größenordnungen.

Während FID und ECD in der modernen Analytik durch massenspektrometrische Detektoren ersetzt werden, hat ihre Verwendung vor allem in der Headspace-Technik noch eine gewisse Berechtigung, da hier die Proben eine eher geringe Matrixbelastung aufweisen. Darüber hinaus sind beide Detektoren sehr schnell einsatzbereit und benötigen auch nach einem Säulenwechsel keine längeren Äquilibrierungszeiten.

In neuerer Zeit werden vor allem Headspace-Methoden mit massenspektrometrischer Detektion entwickelt, angewendet und publiziert. Dabei kann der MS‑Detektor nur bedingt seine Stärken ausspielen, da sich aus den mit Headspace-Technik erfassbaren, eher kleinen Molekülen oft unspezifische Fragmente bilden. Auch der Einsatz von Tandem‑MS‑Techniken, mit dem Ziel die Empfindlichkeit und/oder die Selektivität zu erhöhen, ist aus demselben Grund in der Regel nicht zielführend bzw. aufgrund des geringen Störuntergrundes bei Headspace-Messungen kaum erforderlich. Ein wichtiger Vorteil der massenspektrometrischen Detektion besteht darin, dass isotopenmarkierte ISTDs verwendet werden können. Ein weiteres Argument für den massenspektrometrischen Detektor ist, dass er vielseitiger eingesetzt werden kann, so kann er gleichermaßen für die Detektion reiner Kohlenwasserstoffe wie für kohlenstoffarme substituierte Verbindungen verwendet werden.

## 4 Praktische Aspekte und Störeinflüsse

Die Qualität von Headspace-Analysen hängt aufgrund der speziellen Probenahmebedingungen und der Untersuchung metabolisch unveränderter Biomarker in besonderer Weise von Einflussfaktoren und Störeinflüssen in der präanalytischen Phase (s. u.) ab. Dabei versteht man unter Einflussfaktoren Veränderungen der Analytkonzentration in vivo, d.h. vor der eigentlichen Probenahme (z. B. aufgrund des Probenahmezeitpunkts, durch Tabakrauchen, durch Alkoholkonsum, durch Medikamenteneinnahme oder durch Drogenkonsum). Dagegen bewirken Störeinflüsse während oder nach der Probenahme Veränderungen der Analytkonzentration, z. B. durch Kontamination und Veränderung der Probenmatrix während Transport und Lagerung (Bader et al. 2010). Insbesondere Störeinflüsse lassen sich gut identifizieren und durch entsprechende Vorgaben im Rahmen von Standardarbeitsanweisungen kontrollieren oder minimieren.

### 4.1 Präanalytische Phase

Die sogenannte „präanalytische Phase“ umfasst die Gewinnung der Proben sowie den Transport und die Lagerung des human-biologischen Materials vor der eigentlichen Analyse. Bei diesen Schritten muss auf kontaminations- und verlustfreie Arbeitsprozesse geachtet werden, um reproduzierbare und richtige Ergebnisse zu erhalten. Fehler in der präanalytischen Phase können zu signifikanten Kontaminationen oder zu Analytenverlusten führen, die sich analytisch oder rechnerisch nicht abschätzen und somit nicht ausgleichen lassen.

#### 4.1.1 Gefäße und Materialien

Bei Headspace-Verfahren ist in den Laboratorien auf Sauberkeit und Kontaminationsfreiheit aller verwendeten Gerätschaften und Chemikalien zu achten. Verwendete Glasgeräte zur Herstellung von Vergleichsstandards sowie die Headspace-Gläschen einschließlich Septen und Verschlusskappen sollten ausgeheizt (mehrere Tage bei etwa 200 °C, z. B. in einem Trockenschrank) und nach Möglichkeit sofort verwendet oder separat kontaminationsfrei nur für kurze Zeit aufbewahrt werden. Beim Ausheizen ist darauf zu achten, dass die Septen je nach Material nur bis zu einer bestimmten Temperatur (80–210 °C) stabil sind. Das Durchstechen des Septums mit einer aufgeheizten Nadel kann bei Mehrfachmessungen aus demselben Headspace-Gläschen ebenfalls zu temperaturabhängigen Undichtigkeiten führen (Kolb und Ettre 2006).

#### 4.1.2 Probenahmezeitpunkt

Generell ist die Probenahme zu einem Zeitpunkt durchzuführen, zu dem sich die Analytkonzentration im zu untersuchenden biologischen Material im Gleichgewichtszustand mit der äußeren Belastung befindet. Zur Bestimmung leichtflüchtiger organischer Verbindungen (z. B. aromatischer Kohlenwasserstoffe im Blut) wird das biologische Material am Ende der Exposition oder bei länger dauernder Tätigkeit am Ende der Schicht gewonnen. Die Halbwertszeiten unmetabolisierter Lösungsmittel im Blut variieren von 30 min bis zu einigen Stunden (siehe Tabelle 1). Sofern ein Arbeitsstoff in der MAK- und BAT‑Werte-Liste oder in vergleichbaren Leitlinien aufgeführt sind, sollte die Probenahme zu dem dort angegebenen Zeitpunkt erfolgen (DFG 2025).

#### 4.1.3 Probenahme

Die Probenahme in der Headspace-Analytik erfordert die Verwendung kontaminationsfreier und in einigen Fällen besonders vorbehandelter Utensilien (Probengefäße, Probenahmegeräte, Desinfektionsmittel). Die Empfehlungen zur Probenahme in den durch die Kommission bereits veröffentlichten Standardarbeitsanweisungen für Headspace-Methoden (siehe Tabellen 2, 3 und 4) lassen sich wie folgt zusammenfassen:

Sollen flüchtige Verbindungen in Blut oder Urin bestimmt werden, so ist es wichtig, die gewonnene Probe bis zur Analyse vor Analytenverlusten zu schützen. Dies kann z. B. erreicht werden, indem das Probenmaterial direkt nach der Probenahme in ausgeheizte (und damit kontaminationsfreie) und bereits gasdicht verschlossene sogenannte „Stechampullen“/Headspace-Gläschen überführt wird. Die Headspace-Gläschen dienen dabei sowohl als Lager- als auch Transportgefäß und werden in der Regel vom Labor zur Verfügung gestellt. Leere Headspace-Gläschen sollten nur kurz und, sofern notwendig, nur unter möglichst konstanten und kontaminationsfreien Lagerungsbedingungen aufbewahrt werden.

Für die Blutgewinnung werden Entnahmebestecke bestehend aus Einwegspritzen und ‑kanülen verwendet, wobei für die Headspace-Analytik Venenblutproben mit Antikoagulanszusatz (z. B. EDTA, Heparin) benötigt werden. Für die Desinfektion der Armbeuge sollte verdünnte Wasserstoffperoxidlösung (ca. 3 %) verwendet werden, da Inhaltsstoffe der handelsüblichen Desinfektionsmittel und weitere Fremdstoffe, die während der Lagerung von den Desinfektionsmitteln aufgenommen werden können, eine potenzielle Kontaminationsquelle darstellen. Die aus der Armvene entnommene Blutprobe wird unmittelbar nach der Abnahme gründlich durchmischt, um das Antikoagulans in der Probe zu verteilen. Anschließens wird ein definiertes Aliquot (in der Regel ein bis zwei Milliliter) in das Headspace-Gläschen überführt. Auch das Probenahmebesteck sollte möglichst nur kurz und kontaminationsfrei gelagert werden.

Für die Uringewinnung werden Einweg-Kunststoffgefäße (Urinbecher) verwendet. Diese sind im Fachhandel erhältlich und fassen normalerweise 100 Milliliter. Die Urinprobe wird zum vorgegebenen Probenahmezeitpunkt direkt im Gefäß gesammelt, wobei eine Kontamination vor allem durch Stäube, aber gegebenenfalls auch durch Gase oder Dämpfe am Arbeitsplatz zu vermeiden ist. Für die Bestimmung leichtflüchtiger organischer Substanzen im Urin wird ein definiertes Aliquot (in der Regel ein bis zwei Milliliter) der frischen Spontanurinprobe mit einer Einwegspritze in ein ausgeheiztes Headspace-Gläschen überführt.

#### 4.1.4 Transport, Lagerung und Stabilität der Proben

Blut- und Urinproben sollten möglichst unmittelbar nach der Probenahme in gasdichte Probengefäße überführt und in das Untersuchungslabor übersandt werden. Unter Umständen – in Abhängigkeit von den zu bestimmenden Parametern – können Blut- und Urinproben auch in möglichst voll gefüllten Probengläschen mit minimalem Gasraum versendet werden. Damit wird eine Vorverteilung begrenzt und einem Analytenverlust entgegengewirkt. Beim Transport der Proben ist auf Kontaminationsfreiheit zu achten. Humanproben, bei denen nur eine minimale Wahrscheinlichkeit besteht, dass sie Krankheitserreger enthalten, dürfen als „freigestellte medizinische Probe“ ohne Angabe einer UN-Nummer versendet werden („P 650 light“) (Bundesregierung Deutschland 2021). Dazu muss sich die Probe in einer Dreifachverpackung befinden, bestehend aus einem wasserdichten Primärgefäß, einer wasserdichten Sekundärverpackung und einer ausreichend festen Außenverpackung. Bei flüssigen Stoffen muss auf eine ausreichende Menge absorbierenden Materials zwischen Primärgefäß und Sekundärverpackung geachtet werden. Zudem ist der Paketaufdruck „freigestellte medizinische Probe“ und „exempt human specimen“ vorgeschrieben.

Ist ein Versand direkt nach Probenahme nicht möglich, können die Proben für die Headspace-Analytik für wenige Tage entsprechend den weiter unten angegebenen Bedingungen gelagert werden. Die zur Lagerung genutzten Kühl- und Gefriereinheiten sollten nach Möglichkeit nicht in Laboren stehen, in denen Lösungsmittel gehandhabt werden. Zudem sollten Materialien, die Lösungsmittel enthalten oder freisetzen können, nicht zusammen mit Headspaceproben untergebracht sein. Grundsätzlich ist für viele Analyten eine tiefgekühlte Lagerung von Blut- und Urinproben verlustfrei über mehrere Tage möglich (Ashley et al. 1996; Gill et al. 1988). Ogawa und Sasahara (2012) untersuchten die Lagerungsstabilität von Toluol in Blutproben und stellten fest, dass eine kurzzeitige (bis zu drei Tage) gekühlte Lagerung von Blutproben keine signifikanten Verluste zur Folge hatte. In einer anderen Studie, in der Dichlormethan in Urin untersucht wurde, konnten ebenso keine signifikanten Unterschiede zwischen der Lagerung bei Raumtemperatur und im Kühlschrank gefunden werden (Hoffer et al. 2005). Wichtig ist jedoch in jedem Fall die schnelle Überführung der gewonnenen Proben in gasdichte Probengefäße (Hoffer et al. 2005; Ogawa und Sasahara 2012).

Für einzelne Analyten kann es auch wichtig sein, die Proben dunkel zu lagern. So ergaben eigene Untersuchungen, dass die Lagerungsstabilität halogenierter Kohlenwasserstoffe, insbesondere von Tetrachlorkohlenstoff, höher war, wenn die Proben dunkel gelagert wurden (siehe Anhang).

### 4.2 Probenvorbereitung

Die Probenvorbereitung in der Headspace-Analytik hat das Ziel, die Analyten einer Bestimmung zugänglich zu machen, die Analytkonzentration im Dampfraum über der Probe zu erhöhen oder durch Zugabe eines ISTD die Methodenpräzision zu verbessern.

#### 4.2.1 Überführung von Analyten in flüchtige Verbindungen

Die Vorteile der Headspace-Analytik im Vergleich zu anderen Extraktions- und Analysenverfahren sind erheblich (einfache Probenaufbereitung, effiziente Abtrennung der Analyten von der biologischen Matrix, geringer Störuntergrund in der Chromatographie), so dass das Verfahren vorteilhaft auch bei Stoffen eingesetzt wird, die zwar selbst nicht flüchtig sind, sich aber durch geeignete Maßnahmen in flüchtige Verbindungen umwandeln lassen.

Dies gilt z. B. für die Bestimmung des Kohlenmonoxid‑Hämoglobin (Hb)‑Gehaltes im Blut, die auf der Kohlenmonoxid-Freisetzung und einer anschließenden katalytischen Umwandlung in Methan beruht (Angerer und Zorn 1985). Auch die selbst nicht flüchtige Trichloressigsäure (Stoffwechselprodukt von Trichlorethen, Tetrachlorethen, 1,1,1‑Trichlorethan und anderer aliphatischer Chlorkohlenwasserstoffe) lässt sich nach thermischer Decarboxylierung mit Hilfe der Headspace-Gaschromatographie bestimmen. Das dabei entstehende Chloroform kann sehr empfindlich und spezifisch erfasst werden (Christensen et al. 1988, Will et al. 2017). Trifluoressigsäure, der Metabolit des Halothans, kann nach direkter Veresterung mit Trichlorethanol im Headspace-Gläschen mittels Headspace-Technik quantifiziert werden (Dallmeier und Müller 1982). Schließlich können Analyten auch durch Säurezugabe wie beispielsweise bei der Überführung des Cyanids in Blausäure freigesetzt werden (Eben und Lewalter 1988).

Hinsichtlich der Überführung in flüchtige Verbindungen muss immer bedacht werden, dass jeder Arbeitsschritt und jede Zugabe von Chemikalien zu Analytenverlusten oder einer Probenkontamination führen kann.

#### 4.2.2 Erhöhung der Analytkonzentration im Dampfraum

Die Analytkonzentration im Dampfraum über der Probe hängt vor allem von der Konzentration des Stoffes im Untersuchungsmaterial, vom Verteilungskoeffizienten K sowie vom Phasenverhältnis im Headspace-Gläschen ab (siehe Abschnitt 2.1). Der Verteilungskoeffizient K lässt sich grundsätzlich durch einen Salzzusatz („Aussalzen“) oder eine pH‑Wert-Änderung beeinflussen. Zusätzlich kann eine Temperaturänderung die Analytanreicherung im Dampfraum erhöhen bzw. beschleunigen.

Durch das Aussalzen wird die Löslichkeit des Analyten in der wässrigen Phase herabgesetzt und dadruch eine Erhöhung der Analytkonzentration im Dampfraum erreicht (Grover und Ryall 2005). Hierfür werden meist Ammoniumchlorid, Ammoniumsulfat, Natriumchlorid, Natriumsulfat oder Kaliumcarbonat verwendet (Kolb und Ettre 2006). Ein Zusatz dieser Salze verringert am ehesten die Löslichkeit polarer VOCs in wässriger Probenmatrix, während unpolare Substanzen mit niedrigem K‑Wert kaum beeinflusst werden (Kolb und Ettre 2006). Für einen maximalen Aussalzeffekt ist es dabei wichtig, die Sättigungskonzentration zu erreichen, um Konzentrationsunterschiede und damit ein unterschiedliches Phasengleichgewicht in verschiedenen Proben zu vermeiden. Allerdings enthält Salz oft flüchtige Verunreinigungen und hohe Salzkonzentrationen führen zu einer erhöhten Viskosität der wässrigen Phase wodurch eine längere Thermostatisierungszeit erforderlich ist (Kolb und Ettre 2006). Da das Aussalzen nicht generell von Vorteil ist, muss dies für die jeweiligen Analyten individuell getestet werden.

Auch eine Änderung des pH‑Wertes der Proben kann dazu beitragen, die Analytkonzentration in der Gasphase zu maximieren, indem die Löslichkeit der Analyten in der wässrigen Probe herabgesetzt wird. So werden z. B. flüchtige Säuren durch eine Senkung des pH‑Wertes protoniert und damit weniger löslich, bei Aminen kann durch Erhöhung des pH-Wertes eine Deprotonierung und damit eine Verringerung der Löslichkeit erreicht werden. Bei der Matrix Blut ist eine Zugabe starker Säuren und Basen nicht zu empfehlen, weil dadurch die Koagulation der Blutprobe ausgelöst wird.

Die Zugabe von Säuren oder Laugen kann die Freisetzung von Analyten aus den biologischen Materialien deutlich verändern. Smith et al. (2008) konnten insbesondere durch Ansäuern von Urinproben eine deutliche Steigerung der Konzentration von bestimmten Analyten wie Acetaldehyd, Ethanol, Furan, Hexanal, 2‑Methylfuran, 3-Methylfuran, Octanal, Phenol, Propanal und Toluol in der Dampfphase erzielen. In welchem Ausmaß Zersetzungsreaktionen für die Steigerung der Analytenfreisetzungen verantwortlich waren, wurden dabei nicht untersucht (Smith et al. 2008).

Hinsichtlich der Zugabe von Chemikalien (Salzen, Säuren, etc.) muss jedoch berücksichtigt werden, dass jeder Arbeitsschritt nach der Probenahme und dem Transfer eines Probenaliquots in ein gasdichtes Headspace-Gläschen die Gefahr eines Analytenverlustes oder einer Probenkontamination erhöht.

### 4.3 Störeinflüsse

#### 4.3.1 Blindwerte, Kontaminationen und Analytenverluste

Bei Blindwerten handelt es sich um Verunreinigungen mit den jeweiligen Analyten, die aus den verwendeten Gerätschaften und Chemikalien herrühren. Ashley et al. (1996) konnten zeigen, dass eine Blutentnahme mit unbehandelten Vacutainern^®^ zu signifikant höheren Blutspiegeln von *n*‑Bromoform und *m*-/*p*-Xylol führte, während dies für 1,4‑Dichlorbenzol nicht beobachtet wurde. Eine Dekontamination der Blutentnahmeröhrchen durch entsprechende Vorbehandlung der Vacutainer^®^ war für die betroffenen VOCs demnach erforderlich (Ashley et al. 1992). Auch konnten bei der Untersuchung verschiedener Probenahmegefäße im Rahmen der BTEX-Analytik Ethylbenzol und Xylol-Werte von 11–14 μg/l bzw. 51–65 μg/l detektiert werden. Durch Ausheizen der Septen ließen sich diese Blindwerte deutlich reduzieren (Bader et al. 1994). Beim Vergleich unterschiedlicher Vacutainer^®^-Typen wurden in eigenen Untersuchungen Benzolleerwerte von bis zu 5 μg/l gefunden. Durch Verwendung speziell präparierter Vacutainer^®^-Stopfen ließ sich dieser Blindwert auf das niedrigere Niveau eines alternativen Abnahmesystems (Monovette^®^) reduzieren (siehe Anhang). Das Untersuchungsmaterial kann auch eine externe Kontamination mit den Zielanalyten aufweisen, die z. B. während der Probenahme bzw. Probenaufarbeitung in die Probe gelangen (Heinrich-Ramm et al. 2004).

Kolb und Ettre (2006) heben hervor, dass Blindwerte häufig aus den verwendeten Septen stammen, dass Kontaminationen in dem für die Blindwertmessung verwendeten Wasser vorkommen oder dass Kontamination über die Laborluft in die Probe eingetragen werden. Darüber hinaus weisen Kolb und Ettre (2006) darauf hin, dass es insbesondere bei der Anwendung der Purge & Trap-Technik zu Memory-Effekten kommen kann. Bei dieser Anreicherungstechnik ist es die mögliche Aerosolbildung aufgrund der Gasdurchleitung durch die Probe, die eine Verschleppung von Probenbestandteilen verursacht.

Zu Verlusten kann es durch Verflüchtigung der Analyten aus dem Untersuchungsmaterial, durch Adsorption der Analyten an Materialoberflächen oder durch chemische Reaktionen in der Probe kommen. Auch ein mikrobieller Abbau einzelner Substanzen ist möglich, wenn die Lagerungsbedingungen nicht entsprechend angepasst sind. Zu den bedeutenden, leicht vermeidbaren Ursachen von Analytenverlusten zählt auch die Verflüchtigung durch undichte bzw. mangelhaft verschlossene Headspace-Gläschen (Kolb und Ettre 2006): die Bördelkappen der Probengläser sollten sich nicht oder nur sehr schwer drehen lassen.

Eigene Untersuchungen haben gezeigt, dass sich die Aluminium-Bördelkappen der Headspace-Gefäße nach eintägiger Lagerung im gekühlten (4 °C) und insbesondere im tiefgekühlten Zustand (−20 °C) häufig frei drehen ließen (siehe Anhang). Insbesondere nach einer Probenahme bei Raumtemperatur und anschließender Lagerung der Probengläser bei tiefen Temperaturen können die unterschiedlichen Ausdehnungskoeffizienten der einzelnen Bestandteile der Headspace-Gläschen (Glas, Aluminium, Gummi/Silikon) zu Undichtigkeiten führen. Dieser Effekt kann sowohl zu externer Kontamination als auch zu Analytenverlusten führen und sollte zeitnah nach Erreichen der gewünschten Lagertemperatur überprüft und gegebenenfalls durch erneutes Verbördeln oder Festdrehen von lose sitzenden Crimp- oder Schraubkappen vermieden werden.

In Bezug auf den mikrobiellen Abbau weisen eigene Untersuchungen darauf hin, dass der Zusatz von Natriumchlorid (1 g/ml Probe) bei einer Lagerung bei Raumtemperatur eine Pilzbildung z. B. in Urinproben verhindern kann. So wurde in nicht mit Natriumchlorid stabilisierten Urinproben ein Verlust von Methanol ermittelt, der in den mit Natriumchlorid versetzten Proben nicht auftrat (siehe Anhang).

Spezielle Anwendungen wie z. B. die Verwendung von Probenröhrchen mit Unterdruck (z. B. Vacutainer^®^) zur Aliquotierung und Aufbewahrung von Urinproben können die Gefahr sowohl von Kontaminationen als auch von Analytenverlusten verringern (Kawai et al. 2011).

#### 4.3.2 Geänderte Verteilungsgleichgewichte

Eine Erhöhung der Inkubationstemperatur führt zu einem veränderten Phasengleichgewicht gemäß dem Henry‑Daltonschen Gesetz, denn es erhöhen sich sowohl der Partialdruck des Analyten (erwünschter Effekt) als auch der Partialdruck des Wassers der biologischen Matrix (unerwünschter Effekt). Auch wenn sich die Konzentration des Analyten in der Gasphase im optimalen Fall stärker erhöht als die Konzentration des Wassers, ist ein vermehrter Eintrag von Wasserdampf/Wasser auf die chromatographische Trennsäule bzw. in das Detektionssystem in der Regel ungünstig für die Stabilität/Reproduzierbarkeit der Analytik und die Standzeiten des Headspace‑GC-Systems.

Bei der Verwendung der Matrix Blut für Headspace-Verfahren ist es grundsätzlich wichtig, die Koagulation des Blutes zu vermeiden, die insbesondere bei hohen Temperaturen eintritt. Ist die Blutprobe mit Antikoagulationsmitteln (EDTA, Citrat, etc.) versehen, kann diese für die Headspace-Injektion bis auf 50 °C erwärmt werden. Ohne zugesetztes Antikoagulationsmittel setzt bereits oberhalb von 40 °C Koagulation ein, wodurch eine zuverlässige Einstellung des Verteilungsgleichgewichtes nicht mehr gewährleistet werden kann.

### 4.4 Kalibrierung und Kontrollmaterial

#### 4.4.1 Kalibrierung

Die Qualität der Headspace-Analytik in Bezug auf Präzision, Reproduzierbarkeit und Robustheit wird wesentlich durch die Einstellung und Einhaltung konstanter Rahmenbedingungen (Temperatur- und Drucksteuerung, Verhältnis von flüssiger zu gasförmiger Phase, Äquilibrierdauer, etc.) bestimmt. Diese Bedingungen beeinflussen unmittelbar die Probenäquilibrierung und daraus folgend die Menge an transferierbaren und damit quantitativ erfassbaren Zielanalyten. Die Kalibrierung aus einem Phasengleichgewicht heraus stellt gegenüber der einfachen Injektion von flüssigen Extrakten oder Gasvolumina besondere Anforderungen an die Stabilität des eingesetzten Analysensystems, aber auch an die Kalibrierstandards und deren Herstellung: zur Sicherung reproduzierbarer und richtiger Ergebnisse ist es erforderlich, für jede Analysenmethode ein Kalibrierverfahren zu etablieren, das die Konzentrations- und Verteilungsverhältnisse in der zu untersuchenden Probe möglichst gut widerspiegelt und damit unmittelbar zur Auswertung herangezogen werden kann oder zumindest die Festlegung eines Korrekturfaktors ermöglicht (Kolb und Ettre 2006). Im Regelfall wird für die Herstellung des Kalibriermaterials die jeweilige biologische Matrix (Blut, Plasma/Serum, Urin) verwendet, die dem Untersuchungsgut entspricht und daher auch Einflüsse der Lagerung und der Aufarbeitung sowie Verteilungseffekte zwischen Probenmatrix und Dampfraum berücksichtigt.

Während für die Kalibrierung in Urin gepoolte Individualurine beruflich nicht belasteter Personen verwendet werden können, stellt sich die Kalibrierung in Vollblut komplexer dar: neben der Einstellung des Gleichgewichts zwischen der flüssigen biologischen Matrix und der Gasphase finden auch Verteilungsprozesse zwischen den zellulären Bestandteilen der Probe (z. B. Lipidmembranen), freien Makromolekülen und Agglomeraten (z. B. Proteinen, Lipoproteinen) und dem Plasma statt. Daher ist in Betracht zu ziehen, dass die Gleichgewichtskonzentrationen zwischen den Matrixbestandteilen in einer in vivo gewonnenen Probe anders liegen als in einer in derselben Matrix frisch hergestellten Kalibrierprobe. Weitere Veränderungen und Unterschiede können sich ergeben, wenn Vollblutproben vor der Analyse eingefroren gelagert wurden, da sich die Zusammensetzung und die physikalisch-chemischen Eigenschaften der Matrix durch die Lyse der Erythrozyten verändern. In diesem Zusammenhang sind auch Speziesunterschiede zu beachten: aufgrund unterschiedlicher quantitativer und qualitativer Zusammensetzung des Blutes (z. B. Hämatokrit, Serum/Plasmaproteine, Lipide) ist die Eignung von Tierblut für die Kalibrierung von Gefahrstoffen im Humanblut stets im Einzelfall zu prüfen. Neben der Verfügbarkeit und den Kosten sind bei der Entscheidung für Tier- oder Humanblut als Kalibriermatrix auch mögliche Hintergrundkonzentrationen der Zielparameter zu beachten, die in Humanblut häufig höher liegen als im Blut anderer Spezies (Heinrich-Ramm et al. 2004). Darüber hinaus weichen manche Blut-Gas-Verteilungskoeffizienten wie z. B. für Desfluran, Sevofluran, Isofluran und Methoxyfluran im Blut von neun gängigen Tierspezies von jenen im menschlichen Blut ab, was auf artbedingte Unterschiede in der Triglyceridkonzentration und der Bindung an Hämoglobin, Plasmaproteine und Erythrozytenmembranen zurückzuführen sein könnte (Soares et al. 2012).

In einer Publikation haben Heinrich-Ramm et al. (2004) verschiedene etablierte Kalibrierverfahren für die Headspace-Analyse von aromatischen Verbindungen in Blut im Rahmen eines Ringversuchs verglichen. Dabei wurde eine ethanolische Ausgangslösung von Benzol, Toluol, Ethylbenzol, *m*‑Xylol und *o*‑Xylol (20 000 mg/l) zunächst mit Ethanol zu Stammlösungen mit Konzentrationen zwischen 100 mg/l und 800 mg/l verdünnt und anschließend bis in arbeitsmedizinisch relevante Konzentrationsbereiche (≈ 5–500 μg/l) weiter verdünnt. Die Verdünnungsschritte wurden dabei mit Vollblut (defibriniertes Pferdeblut, natives Menschenblut) oder physiologischer Kochsalzlösung durchgeführt. Anschließend erfolgte eine Headspace-GC-Analyse mit den jeweils in den Laboratorien verwendeten Analysengeräten. Durch den Austausch unterschiedlich vorbereiteter Kalibrierstandards wurden zudem Einflüsse der verwendeten Analysentechnik untersucht. Die Studie zeigte, dass die Herkunft des verwendeten Vollbluts (Pferd, Mensch) zu signifikanten Unterschieden in der Steigung der Kalibrierfunktionen führt und dass die aufwändigere Verdünnung in Messkolben ebenso wie die ausschließliche Verdünnung mit physiologischen Kochsalzlösungen gegenüber einer rein volumetrischen Verdünnung (Pipettieren berechneter Volumina statt Verwendung von graduierten Messkolben) mit Vollblut in Glasampullen zu flacheren Kalibrierkurven und damit zu einer Überbestimmung führt. Das wesentliche Ergebnis der umfangreichen Untersuchungen war die Empfehlung, eine kombinierte Verdünnung zunächst in physiologischer Kochsalzlösung, dann in Vollblut durchzuführen und das Pipettieren vorausberechneter Volumina der Verwendung von Messkolben vorzuziehen. Mit diesem Verfahren wurde eine gute Übereinstimmung mit den Sollwerten des 24. Ringversuchs des G‑EQUAS (German External Quality Assessment Scheme, https://app.g-equas.de) erreicht (Heinrich-Ramm et al. 2004).

Die Arbeit von Heinrich-Ramm et al. (2004) belegt, dass die Ergebnisse der Headspace-Analytik stark von Matrixeffekten abhängen, insbesondere in Bezug auf die Herstellung der Kalibrierstandards und die dafür verwendete Matrix. Es ist zu erwarten, dass diese Effekte bei weniger komplexen Matrices (Serum/Plasma, Urin) geringer ausfallen. Allerdings ist auch in diesen Fällen eine effiziente und zügige Herstellung der Kalibrierstandards wichtig, um Analytenverluste während dieses Prozesses zu minimieren.

Eine Möglichkeit, bei einer hinreichend hohen Analytkonzentration mit Matrixproblemen umzugehen, besteht in der einfachen Verdünnung der Messlösung. So existieren beispielsweise Vorschriften für die Bestimmung des Blutethanolgehaltes, die eine 1 ∶ 10‑Verdünnung der Blutproben mit wässrigem Medium vorsehen (Kolb und Ettre 2006). Die Möglichkeit, Matrixeffekte in Vollblutproben durch Verdünnung zu minimieren, wurde auch von Alonso et al. (2013) an zwölf VOCs mittels SPME‑HS‑GC‑MS untersucht. Die Autoren beschreiben, dass der Einfluss der Blutmatrix auf die Wiederfindung der Analyten von deren Siedepunkt abhängt. Eine 1 ∶ 5‑Verdünnung mit Wasser verbesserte die Wiederfindung und erlaubte die quantitative Extraktion der meisten Analyten. Im Fall von 1,2‑Dichlorbenzol mit einem Siedepunkt von 180,5 °C konnte der Matrixeffekt durch alleinige 1 ∶ 5‑Verdünnung mit Wasser jedoch nicht kompensiert werden (25%ige Wiederfindung).

Mit Blick auf die Herstellung der Kalibrierstandards weisen die Analytenverluste bei ausschließlicher Verwendung von physiologischer Kochsalzlösung auch darauf hin, dass ein möglichst einfaches und zügiges Vorgehen angestrebt werden sollte (Heinrich-Ramm et al. 2004). Kolb und Ettre (2006) empfehlen, Kalibrierstandards für die Headspace-Analytik stets frisch aus den Stammlösungen anzusetzen. Dabei ist es bei Multisubstanzstandards ratsam, die Analyten in der umgekehrten Reihenfolge ihrer Flüchtigkeit in die Matrix zu dotieren. Dies ist vor allem bei sehr flüchtigen Substanzen mit niedrigen Verteilungskoeffizienten wichtig. Zur Aufbewahrung werden die Stammlösungen in gut schließende Gewindegläschen gefüllt, die möglichst vollständig gefüllt sein sollten.

Beim Ansetzen der Stammlösungen wird, in Abhängigkeit von Analyten und Matrix, ein Lösungsmittel im jeweiligen Glasgefäß vorgelegt, danach werden die flüchtigen Analyten eingewogen. Alternativ zur Einwaage pipettierter Volumina kann zum Ansetzen und Verdünnen von Stamm-, Dotier- und Messlösungen auch mit Mikroliterspritzen gearbeitet werden, die ein möglichst geringes Totvolumen aufweisen sollten. Dabei müssen Gerätschaften und Lösungen Raumtemperatur angenommen haben, um Abweichungen in den pipettierten Volumina zu vermeiden, da sonst nicht-lineare Kalibriergeraden resultieren können (Kolb und Ettre 2006).

Ob eine Kalibrierung in Wasser, ähnlich wie bei anderen Analysenverfahren, möglich und sinnvoll ist, muss im Einzelfall geprüft werden. Aufgrund der hohen Volatilität der meisten Zielsubstanzen der Headspace-Analytik ist jedoch zu erwarten, dass eine Matrixkalibrierung insbesondere in Bezug auf Analytenverluste und Reproduzierbarkeit zu bevorzugen ist.

#### 4.4.2 Interne Standards (ISTDs)

Vorbedingung für die Anwendung eines ISTD ist dessen optimale chromatographische Abtrennung oder spektrometrische Differenzierung von dem zu untersuchenden Stoff. Die Konzentration des ISTD in der Gasphase soll nach Möglichkeit im gleichen Bereich wie die des Analyten liegen. Darüber hinaus sollten Analyt und ISTD in ihrem physikochemischen Verhalten, z. B. ihrem Dampfdruck, möglichst ähnlich sein.

Zweckmäßigerweise werden deshalb, z. B. für die Analyse von Alkoholen, auch Alkohole als ISTDs eingesetzt und für Aromaten entsprechend aromatische Kohlenwasserstoffe. Aufgrund ähnlicher Polarität unterliegen diese strukturanalogen Verbindungen denselben Matrixeffekten wie die eigentlichen Analyten und können so Matrixunterschiede zwischen den Proben kompensieren. ISTDs mit einem weiten Anwendungsbereich sind Substanzen wie beispielsweise* tert*-Butanol, Benzol, 2‑Butanon (Methylethylketon) und Aceton. Speziell für die massenspektrometrische Detektion eignen sich strukturidentische isotopenmarkierte Verbindungen, die sich von dem eigentlichen Zielanalyten durch eine Massendifferenz von mindestens 2 Dalton unterscheiden. Solche Standards sind allerdings nicht für alle Zielanalyten verfügbar.

Der zu analysierenden Probe wird der ISTD üblicherweise in wässriger oder alkoholischer Lösung zugesetzt. Bei Proben, die bereits in Headspace-Gläschen abgefüllt sind, kann der ISTD auch mit einer Injektionsspritze durch das Septum eingebracht werden, um ein erneutes Öffnen des Headspace-Gläschens zu vermeiden. Da ein Öffnen des Fläschchens zu Analytenverlust oder Probenkontamination führen kann, kann auf den Zusatz eines ISTD auch verzichtet werden, sofern dieser für die analytische Zuverlässigkeit nicht erforderlich ist.

#### 4.4.3 Kontrollmaterial

Zur Sicherung der Qualität der Analysenergebnisse sollte auch bei der Headspace-Analytik gemäß den Richtlinien der Bundesärztekammer und den Angaben in dem von der Kommission veröffentlichten allgemeinen Kapitel verfahren werden (Bader et al. 2010; Bundesärztekammer 2023).

Zur Präzisionskontrolle wird in jeder Analysenserie mindestens eine Qualitätskontrollprobe untersucht, die eine konstante Konzentration der zu untersuchenden Analyten aufweist. Da für die Headspace-Analytik keine käuflichen Kontrollmaterialien zur Verfügung stehen und dementsprechend auch keine zertifizierten Kontrollmaterialien, muss dieses selbst hergestellt werden. Dazu wird Poolurin oder Vollblut mit entsprechenden Mengen der Analyten dotiert, das Material in Headspace-Gläschen aliquotiert und bei ca. −20 °C tiefgefroren aufbewahrt. Die Stabilität der so hergestellten und gelagerten Materialien wird mittels Kontrollkarten überprüft.

Hinsichtlich der Stabilität von selbst hergestelltem Qualitätskontrollmaterial für die Bestimmung von Aromaten und anderen Lösungsmitteln in Blut gelangten beispielsweise Heinrich-Ramm et al. (2004) zu dem Schluss, dass diese Materialien nur wenige Monate stabil und somit für die langfristige Qualitätskontrolle nur bedingt geeignet sind.

Um die Richtigkeit eines Analysenverfahrens zu prüfen, sind neben der internen Qualitätssicherung auch Angebote der externen Qualitätssicherung zu nutzen. Das von der Deutschen Gesellschaft für Arbeitsmedizin und Umweltmedizin initiierte externe Qualitätssicherungsprogramm G-EQUAS ist weltweit der einzige Ringversuch mit einem breiten Spektrum an arbeitsmedizinisch relevanten Headspace-Parametern. Insgesamt werden im Rahmen dieses Ringversuchs zweimal jährlich vier Materialien angeboten: Benzol, Toluol, Xylol (*m*-, *o*-, *p-*) und Ethylbenzol in Blut sowie in Urin, darüber hinaus Dichlormethan, Trichlormethan, Tetrachlormethan, 1,2‑Dichlorethan, 1,1,1‑Trichlorethan, Trichlorethen und Tetrachlorethen in Blut sowie schließlich Methanol, *n*‑Butanol, Aceton, 2‑Butanon (Methylethylketon), Methyl‑*n*‑butylketon, Methylisobutylketon, Tetrahydrofuran und Methyl‑*tert*‑butylether in Urin.

## 5 Publizierte HBM-Methoden und Beurteilungswerte

### 5.1 Publizierte HBM-Methoden

#### 5.1.1 Publizierte Methoden der Kommission

Bis Mitte 2025 wurden von der Arbeitsgruppe „Analysen in biologischem Material“ insgesamt 36 Headspace-Methoden veröffentlicht, mit denen das Biomonitoring für 66 Arbeitsstoffe spezifisch und empfindlich durchgeführt werden kann. Die Tabellen 2, 3 und 4 geben einen Überblick über die von der Kommission publizierten Headspace-Methoden für ein Human-Biomonitoring in Urin, Blut und Ausatemluft.

Zwischen 1978 und 1983 wurden sechzehn HS-GC-Methoden zu einer Headspace-Technik-Sammelmethode zusammengefasst, die einen weiten Bereich der industriell verwendeten Lösungsmittel abdeckt (Machata und Angerer 1983). Mit Ausnahme des Acetons, das sowohl in Blut als auch in Urin bestimmt werden konnte, wurden die Bestimmung dieser Parameter ausschließlich für Blut- bzw. Serum beschrieben.

Weitere Headspace-Methoden für Einzelstoffe wurden zwischen 1980 und 1988 publiziert. Diese Methoden erlauben die Quantifizierung von Ameisensäure in Urin (Angerer und Schaller 1980), Trichloressigsäure (Angerer und Eben 1980) sowie Trifluoressigsäure (Dallmeier und Müller 1982) in Blut, Cyanid in Blut (Eben und Lewalter 1988) sowie die Bestimmung des CO‑Hb‑Wertes in Blut (Angerer und Zorn 1985). Diese Methoden wurden nicht in die Headspace-Technik-Sammelmethode aufgenommen, da sie eine thermische Zersetzung (Trichloressigsäure) (Angerer und Eben 1980), eine Veresterung (Trifluoressigsäure) (Dallmeier und Müller 1982), eine Freisetzung durch Säure (Cyanid) (Eben und Lewalter 1988) oder eine katalytische Umwandlung des Analyten (Ameisensäure; CO‑Hb) (Angerer und Schaller 1980; Angerer und Zorn 1985) erfordern und somit nicht dem allgemeinen Vorgehen der Sammelmethode entsprechen.

Die zu Beginn der 1990er-Jahre publizierten Methoden zur Bestimmung halogenierter Kohlenwasserstoffe (Angerer et al. 1991) und zur Bestimmung von Benzol und Alkylbenzolen (Angerer et al. 1994) lassen die Weiterentwicklung der Labortechnik erkennen mit Nachweisgrenzen, die um den Faktor 2 (halogenierte Kohlenwasserstoffe) oder 5 (Benzol und Alkylbenzole) niedriger liegen als in den zuvor publizierten Methoden.

Mit der 1996 veröffentlichten Methode „Alkohole und Ketone in Blut und Urin“ (Angerer et al. 1996) wurden zahlreiche alkoholische Arbeitsstoffe erstmals in die Methodensammlung aufgenommen und zudem ein breites Parameterspektrum auch in der Matrix Urin zugänglich gemacht. Mit dem Addendum „Tetrahydrofuran (THF) in Urin“ wurde die Methode im Jahr 2012 noch um einen weiteren Parameter ergänzt (Blaszkewicz und Angerer 2012), so dass nun in einem Analysengang zwölf Analyten simultan bestimmt werden können.

Die Verbreitung und stete Weiterentwicklung der Headspace‑GC‑MS-Kopplung als sensitives und zuverlässiges Verfahren zur Bestimmung von Arbeitsstoffen in biologischem Material hat eine Überarbeitung und Aktualisierung der von der Arbeitsgruppe „Analysen in biologischem Material“ publizierten Analysenmethoden erforderlich gemacht. Mit der Methode Methylquecksilber in Blut (Hoppe und Heinrich-Ramm 2006) wurde erstmals eine HS‑GC‑Methode mit massenspektrometrischer Detektion in der Methodensammlung publiziert. Mit den Methoden zur Bestimmung von Trichloressigsäure in Urin (Will et al. 2017), Methyl-*tert*‑butylether in Blut und Urin (Hoppe et al. 2018), Aromaten im Blut (Göen et al. 2018), Aromaten in Urin (Van Pul et al. 2018), Alkoholen, Ketonen und Ethern in Urin (Göen et al. 2020) sowie von halogenierten Kohlenwasserstoffen in Blut (Göen et al. 2021) folgten weitere Biomonitoring-Methoden, die nun alle dem Stand der Technik entsprechend die Massenspektrometrie als nachweisstarkes Detektionsverfahren nutzten. Zudem werden, wie bei Van Pul et al. (2018) mit der ITEX-Technik oder bei Roßbach et al. (2019) mit der SPDE-Anreicherung, zunehmend auch neue dynamische Headspace-Techniken eingesetzt, die deutlich empfindlichere Analysen ermöglichen.

In der Arbeitsgruppe „Analysen in biologischem Material“ der Kommission wurde aktuell auch eine Headspace-Methode zur Bestimmung von Furan in der Ausatemluft entwickelt und verabschiedet (Ziener et al. 2024), da sich für die Matrices Blut oder Urin keine Methoden entwickeln ließen, die die zuverlässige Erfassung und Beurteilung einer Furanexposition erlaubt hätten.

#### 5.1.2 International publizierte Biomonitoring-Methoden

Für die Erstellung eines Überblicks über international publizierte Headspace-Methoden zur Bestimmung von Biomonitoring-Parametern in Blut und Urin wurde eine Literaturrecherche durchgeführt. Die Recherche erfolgte in PubMed und Scopus mit den Suchbegriffen: (1) „Headspace“ AND „urine“ AND „occupational“ oder (2) „Headspace“ AND „blood“ AND „occupational“. Duplikate oder Publikationen, in denen keine Headspace-Methoden dargestellt waren, wurden durch manuelle Titel- und Abstractsuche ausgeschlossen. Aus den verbliebenen Studien wurden die relevanten Informationen zu den eingesetzten analytischen Verfahren mittels Volltextsuche extrahiert. Methodenpublikationen, in denen keine Informationen zu Nachweis- oder Bestimmungsgrenzen enthalten waren, wurden ausgeschlossen.

Headspace-Methoden wurden auch für andere Matrices wie Speichel, Ausatemluft, Atemkondensat oder Gewebeproben entwickelt und publiziert. Diese Arbeiten wurden hier größtenteils nicht berücksichtigt, da sich für quantitative Analysen im arbeitsmedizinischen Human-Biomonitoring aufgrund der zumeist bekannten Substanzkinetik (Aufnahme, Verteilung, Metabolisierung, Elimination) die Bestimmung in Urin sowie Blut, Serum und Plasma etabliert hat. Aus diesem Grund beziehen sich auch die meisten Beurteilungswerte in biologischem Material, z. B. BAT, BLW oder BAR auf die Matrices Blut und Urin.

Die Tabellen 5 und 6 geben einen Überblick über die im internationalen Schrifttum publizierten Headspace-Methoden für Biomonitoring-Parameter in Urin sowie Blut, Serum und Plasma. Dabei wurden die Analyten in die Gruppen „aromatische Kohlenwasserstoffe“, „aliphatische Kohlenwasserstoffe“, „halogenierte Kohlenwasserstoffe“, „Alkohole, Aldehyde, Ketone und Ether“, „Inhalationsnarkotika“ und „Sonstige“ eingeteilt. Es sind die verwendeten Analysenmethoden, die erreichten Nachweis- und Bestimmungsgrenzen sowie bei den Multimethoden die Anzahl der parallel bestimmbaren Analyten angegeben. Verständlicherweise finden sich in dieser Aufstellung vornehmlich Methoden für die Bestimmung von leichtflüchtigen Kohlenwasserstoffen, z. B. von BTEX‑Aromaten in Blut, chlorierten Kohlenwasserstoffen (CKWs) in Blut oder Alkoholen und Ketonen in Urin.

Bei einem genaueren Blick auf die publizierten Methoden fällt auf, dass sich die älteren Publikationen vor allem auf besonders leicht flüchtige Substanzen beziehen; in diesen Fällen traten zumindest in der Vergangenheit eher hohe Konzentrationen am Arbeitsplatz und im humanbiologischen Material auf. Für die Messung dieser Belastungen war die Leistungsfähigkeit der klassischen statischen Headspace-Technik ohne Anreicherung in der Regel ausreichend. Zunächst wurden vornehmlich unspezifische Detektionsverfahren wie die Flammenionisationsdetektion für aliphatische und aromatische Kohlenwasserstoffe (z. B. Kawai et al. 2003) sowie die Elektroneneinfangdetektion für halogenierte Kohlenwasserstoffe (z. B. da Silva et al. 1999) eingesetzt. In den letzten Jahren wurden zunehmend Anreicherungstechniken vor der Probenaufgabe sowie die massenspektrometrische Detektion (z. B. Rutkiewicz et al. 2011) verwendet, um zum einen niedrigere Nachweis- und Bestimmungsgrenzen und zum anderen zuverlässigere Analysenergebnisse zu erhalten.

Durch die Verbesserung der analytischen Sensitivität und Spezifität können mittlerweile auch Analyten, die nur zu einem sehr geringen Anteil mit dem Urin ausgeschieden werden, quantifiziert werden. Dieser Trend spiegelt sich in der Literaturübersicht beispielsweise für Stoffe wie Benzol, Toluol sowie *m*-, *o*- und *p*‑Xylol wider und ermöglicht aufgrund der längeren Halbwertszeit dieser Stoffe im Urin gegenüber Blut eine zuverlässigere Bestimmung der beruflichen Belastung. Darüber hinaus ist die Abgabe einer Urinprobe nicht invasiv und wird von den Beschäftigten besser akzeptiert als eine Blutentnahme.

Eine häufige und allgemein bekannte Anwendung der HS‑GC ist die Blutalkoholbestimmung, die in der Forensik meist im Zusammenhang mit Delikten im Straßenverkehr durchgeführt wird. Dabei muss die Alkoholkonzentration stets durch zwei unabhängige Verfahren bestimmt werden (Aderjan et al. 2011). Eines der beiden derzeit für forensische Zwecke zugelassenen Verfahren zur Blutalkoholbestimmung beruht auf der statischen HS‑GC‑FID‑Methode von Machata aus dem Jahr 1964 (Kolb und Ettre 2006; Machata 1967), die den Beginn der quantitativen HS‑GC darstellt. Mittlerweile werden neben Flammenionisationsdetektoren auch Massenspektrometer zur Detektion eingesetzt (Cordell et al. 2013). Ein internationaler Ringversuch zur Bestimmung von Ethanol in Blut und Serum wird von der GTFCh (Gesellschaft für Toxikologische und Forensische Chemie) angeboten (http://www.arvecon.de/).

In der wissenschaftlichen Literatur finden sich auch Methoden, deren praktische Anwendung mit Blick auf die physikochemischen Voraussetzungen und Begrenzungen der Headspace-Analytik kritisch erscheint. Bei diesen Methoden werden z. B. schwerflüchtige Verbindungen mit sehr hohen K‑Werten mittels Headspace-Technik quantifiziert: Chlorphenole (2‑CP, 2,4‑DCP, 2,4,6‑TCP, 2,3,4,6‑TeCP sowie PCP) in Humanurin (ohne Hydrolyse) mit Headspace-SPME‑GC‑MS (Lee et al. 1998), Organochlorpestizide (HCB, *β*‑HCH, Heptachlorepoxid, DDE sowie DDT) und PCBs in Humanserum mit Headspace-SPME‑GC‑ECD (López et al. 2007), Organochlorpestizide (HCB, Heptachlor, DDEs, DDTs, DDDs, Chlordan, Dieldrin, etc.) in Humanserum mit Headspace-SPME‑GC‑MS (Kim et al. 2013), Dinitroanilin-Herbizide in Blut und Urin mit Headspace-SPME‑GC‑ECD (Guan et al. 1998) oder persistente organische Schadstoffe (POP‑Pestizide sowie PCBs) in Humanserum mit Headspace-SPME‑GC‑MS (Flores-Ramírez et al. 2014). Diese Methoden wurden in die tabellarische Übersicht (Tabelle 5und 6) nicht mit aufgenommen.

Exemplarisch sei an dieser Stelle auch auf einige arbeits- und umweltmedizinische Headspace-Anwendungen unter Verwendung alternativer Matrices hingewiesen: zum einen sind Methoden zur Bestimmung von Benzol (Menezes et al. 2009), Styrol (Fields und Horstman 1979; Guillemin und Berode 1988) oder 1,1,2‑Trichlor-1,2,2‑trifluorethan (Woollen et al. 1990) in der Ausatemluft publiziert worden oder zur Bestimmung von Toluol in Atemkondensat (Maniscalco et al. 2006). Zum anderen finden sich Methoden für die Bestimmung von 2‑Butanon (Methylethylketon), Isopropylalkohol und *N,N*‑Dimethylformamid im Speichel von Arbeitern in der Lederindustrie (Wang und Lu 2009), von 2- bis 4‑Ring‑PAK im Speichel von Rauchern und Nichtrauchern (Martín Santos et al. 2020) und eine Methode für die Bestimmung von Toluol, Ethylbenzol, Xylol und Styrol in Speichel (Gherardi et al. 2010). Schließlich sind auch Headspace-Methoden und -Anwendungen publiziert, die Gewebeproben als Matrix nutzen. So finden sich beispielsweise Methoden zur Bestimmung von Nitromethan als Metabolit des Chlorpikrins in Schweineleberproben mit statischer HS-GC‑MS (Halme et al. 2015), zur Bestimmung von Ethylglucuronid in Plazentagewebe und Plazentaperfusat mit Headspace-SPME‑GC‑MS (Matlow et al. 2012) oder zur Bestimmung von 1,1‑Difluorethan in Blut, Urin und Gehirnproben mit statischer HS-GC‑FID (Avella et al. 2008).

### 5.2 Beurteilungswerte HBM

Die Kommission hat für zahlreiche Parameter, die mit Headspace-Verfahren bestimmt werden oder bestimmt werden können, Beurteilungswerte aufgestellt. Darüber hinaus existieren weitere Beurteilungswerte von anderen wissenschaftlichen Gremien, insbesondere vom Ausschuss für Risikobeurteilung (Committee for Risk Assessment, RAC) der Europäischen Chemikalienbehörde (European Chemicals Agency, ECHA) (RAC 2025) und der American Conference of Governmental Industrial Hygienists (ACGIH) (ACGIH 2025). Eine Übersicht über diese Werte findet sich in Tabelle 7. Die Beurteilungswerte wurden vornehmlich für Parameter aufgestellt, für die die Headspace-Technik schon lange etabliert ist, wie beispielsweise für die BTEX‑Aromaten und die kurzkettigen halogenierten Kohlenwasserstoffe in Blut sowie für Alkohole, Ketone und Ether in Urin. Bei den Beurteilungswerten handelt es sich in der Mehrzahl um toxikologisch begründete Grenzwerte (BAT, BEI^®^, BLV), die eine Beurteilung eines möglichen Gesundheitsrisikos erlauben. Darüber hinaus gibt es insbesondere für kanzerogene Gefahrstoffe Beurteilungswerte, die entweder die Abgrenzung der beruflichen Belastung von der allgemeinen Hintergrundbelastung (BAR) erlauben oder über eine Exposition-Risiko-Beziehung mit einem definierten zusätzlichen Lebenszeit-Krebsrisiko verknüpft werden können. Hierzu dienen die Expositionsäquivalente für krebserzeugende Arbeitsstoffe (EKA), die beispielsweise für die Biomonitoring-Parameter „Benzol in Urin“ und „Tetrachlorethen in Vollblut“ aufgestellt wurden (DFG 2025).

Neben den von den wissenschaftlichen Gremien publizierten Beurteilungswerten können auch Daten aus Bevölkerungsstudien herangezogen werden, um Informationen zur allgemeinen Hintergrundbelastung zu erhalten. Tabelle 8 fasst Daten zusammen, die in der internationalen Literatur publiziert wurden. Tabelle 9 zeigt die Hintergrundkonzentrationen verschiedener Parameter, die mit Headspace-Verfahren für die US‑amerikanische Allgemeinbevölkerung im Rahmen von NHANES (National Health and Nutrition Examination Survey) in den Centers for Disease Control and Prevention (CDC) erhobenen wurden. Grundsätzlich ist darauf hinzuweisen, dass Beurteilungswerte, die als Konsens von Expertenmeinungen erarbeitet wurden (Tabelle 7), deutlich belastbarer sind. Bei den Referenzwerten sind zudem die nur regionale Repräsentativität, Untergruppen- und Lebensstil-Effekte sowie die befristete Gültigkeit aufgrund sich ändernder Hintergrundbelastungen zu beachten (Göen et al. 2012).

Unabhängig von der Art des Beurteilungswertes ist die Beachtung des Probenahmezeitpunkts für die Bestimmung von leichtflüchtigen Verbindungen, die typischerweise mit den Headspace-Techniken erfasst werden, von essenzieller Bedeutung. Da die Elimination von beispielsweise leichtflüchtigen Kohlenwasserstoffen aus dem Blut sehr schnell erfolgt, muss die Probenahme unmittelbar nach Ende der Exposition erfolgen. Die Halbwertszeiten der wichtigsten der Headspace-Analytik zugänglichen Gefahrstoffe sind in Tabelle 1 aufgeführt.

## 6 Resümee

Die gaschromatographische Dampfraumanalyse nutzt gut bekannte und reproduzierbare physikochemische Verteilungsvorgänge zur Abtrennung von flüchtigen Verbindungen aus ihrer biologischen Matrix. Die Hauptvorteile dieser sogenannten „Headspace-Analytik“ liegen in der sehr effizienten Trennung der Analyten von der Matrix, in der meist nur sehr wenige Schritte umfassenden Probenvorbereitung sowie in der guten Automatisierbarkeit.

Die bedeutendsten Herausforderungen bei der Anwendung der Headspace-Analytik in der arbeits- und umweltmedizinischen Praxis liegen in der

- Festlegung der Rahmenbedingungen zur Probenahme (v. a. Probenahmezeitpunkt),
- Vermeidung von Kontaminationen und Analytenverlusten in der präanalytischen Phase,
- adäquaten Kalibrierung der Verfahren (v. a. hinsichtlich Matrixauswahl und Herstellung von Vergleichsstandards).

Die in der Übersicht zusammengestellten Headspace-Verfahren, sowohl die von der Kommission entwickelten und publizierten, als auch die weiteren in der wissenschaftlichen Literatur beschriebenen Verfahren, decken grundsätzlich die in der arbeits- und umweltmedizinischen Praxis benötigten Parameter gut ab. Dabei zeigen insbesondere die neueren Verfahren eine Nachweisempfindlichkeit, die eine Bestimmung der Parameter auch im Bereich der Hintergrundbelastung der Allgemeinbevölkerung ermöglicht. Dies lässt sich vor allem auf die in den letzten Jahren zunehmend eingeführten Anreicherungstechniken und den Einsatz der Massenspektrometrie als Standarddetektionsmethode zurückführen. Die Headspace-Analytik ist damit trotz ihrer langen Historie und des begrenzten Anwendungsbereichs auf flüchtige Verbindungen eine nach wie vor bedeutsame Methode des Human-Biomonitorings in der Arbeits- und Umweltmedizin.

**Tab.1:** Halbwertszeiten der wichtigsten der Headspace-Analytik zugänglichen Gefahrstoffe

**Substanz (Synonym)**	**Analyt**	**Matrix**	**Ausscheidungsmaximum**	**Eliminationskinetik**	**Halbwertszeit**	**Literatur**
Aceton	Aceton	Alveolarluft	–	–	4,3 ± 1,1 h	Wigaeus et al. 1981
		Blut	–	linear	3 h	DiVincenzo et al. 1973
			–	–	5,8 h	Wang et al. 1994
		Kapillarblut	–	monoexponentiell	4,3 ± 1,0 h	Ernstgård et al. 1999
		venöses Blut	–	–	6,1 ± 0,7 h	Wigaeus et al. 1981
		arterielles Blut	–	–	3,9 ± 0,7 h	Wigaeus et al. 1981
		Urin	3–3,5 h	–	–	Wigaeus et al. 1981
			2–4 h	–	8 h	Pezzagno et al. 1986
			2 h	biphasisch	8–9 h	Blaszkewicz et al. 1991
Benzol	Benzol	Ausatemluft	–	triphasisch	0,7–1,7 h; 3–4 h; 20 h	Sherwood 1972
			–	–	4 h; 4 d	Sato et al. 1975
		Blut	–	exponentiell	≈ 30 min	Angerer 1983
2-Butanon (Methylethylketon)	2-Butanon	Ausatemluft	–	–	40–60 min	Ong et al. 1991; Tada et al. 1972
		Blut	–	biphasisch	30 min; 81 min	Liira et al. 1988
			–	erster Ordnung	49 min	Brown et al. 1987; Dick et al. 1988
			–	*–*	270 min (mathematisches Modell)	Angerer 1990
		Urin	–	–	1,5 h (1–2,3 h) (nach inhalativer Exposition und dermaler Aufnahme aus der Dampfphase); 2,7 h (2,3–4,3 h) (nach dermaler Aufnahme aus der Dampfphase)	Brooke et al. 1998
Chlorbenzol	Chlorbenzol	Blut	–	biphasisch	53 min; 150 min	Knecht und Woitowitz 2000
Cyclohexan, Cyclohexanon, Cyclohexanol	Cyclohexanol	Urin	Expositionsende	–	1,5 h	Mráz et al. 1998
Dichlormethan (Methylenchlorid)	Dichlormethan	Blut	–	–	5–40 min	Riley et al. 1966 nach ACGIH 2005
			–	–	4,3 h und 8,1 h (n = 2; 36 h nach akuter Vergiftung)	Poli et al. 2005
		Urin	–	–	40 min	DiVincenzo et al. 1972
			Expositionsende	–	210–410 min	Sakai et al. 2002
			–	–	3,8 h und 7,5 h (n = 2; 36 h nach akuter Vergiftung)	Poli et al. 2005
Ethylbenzol	Ethylbenzol	Alveolarluft	–	mehrphasisch	t_1_: < 1 h	Tardif et al. 1997
		Blut	–	biphasisch	0,5 h; 1,81 h	Knecht et al. 2000
			–	mehrphasisch	t_1_: < 1 h	Tardif et al. 1997
		Urin	–	biphasisch	0,69 h; 19,2 h	Janasik et al. 2008
Halothan (2‑Brom-2‑chlor-1,1,1‑trifluorethan)	Halothan	Ausatemluft	–	linear, triphasisch	t_1_: 20–30 min; t_3_: 2 h	Henschler 1983
	Trifluoressigsäure	Blut	–	–	40–60 h	Henschler 1983
		Urin	–	–	48–66 h	Henschler 1983
*n*‑Heptan	1‑Heptanol	Urin	3,15 h	mehrphasisch	t_1_: 1,70 h; t_2_: 9,68 h	Rossbach et al. 2018
	2‑Heptanol	Urin	3,24 h	mehrphasisch	t_1_: 1,46 h; t_2_: 8,26 h	Rossbach et al. 2018
	3‑Heptanol	Urin	3,24 h	mehrphasisch	t_1_: 1,46 h; t_2_: 7,99 h	Rossbach et al. 2018
	4‑Heptanol	Urin	3,32 h	mehrphasisch	t_1_: 1,60 h; t_2_: 7,75 h	Rossbach et al. 2018
	2‑Heptanon	Urin	5,48 h	mehrphasisch	t_1_: 2,53 h; t_2_: n. a.	Rossbach et al. 2018
	3‑Heptanon	Urin	3,10 h	mehrphasisch	t_1_: 2,14 h; t_2_: 9,05 h	Rossbach et al. 2018
	Heptan-2,5-dion	Urin	3,92 h	mehrphasisch	t_1_: 2,87 h; t_2_: 8,85 h	Rossbach et al. 2018
			–	–	3,4 ± 1,5 h	Filser et al. 1996
Isopropylbenzol (Cumol)	Isopropylbenzol	Blut	–	biphasisch	0,49 h; 1,61 h	Knecht et al. 2000
Kohlenmonoxid	CO‑Hb	Blut	–	biphasisch	1,6 h; 30,9 h	Cronenberger et al. 2008
			–	–	320 min (128–409 min)	Peterson und Stewart 1975
Methanol	Methanol	Ausatemluft	–	monophasisch	1,5 h	Dutkiewicz 1978
			–	–	1,38 ± 0,86 h	Batterman et al. 1998
		Blut	–	erster Ordnung	2,25 h	Ferry et al. 1980 a, b
			–	–	1,44 ± 0,33 h	Batterman et al. 1998
		Urin	–	–	1,5–2,0 h	Šedivec et al. 1981
			–	–	1,55 ± 0,67 h	Batterman et al. 1998
Methyl-*tert*-butylether (2-Methoxy-2‑methylpropan)	Methyl-*tert*-butylether	Ausatemluft	–	–	1,3–2,9 min	Lindstrom und Pleil 1996
		Alveolarluft	–	triphasisch	0,25 ± 0,07 h; 0,64 ± 0,15 h; 1,74 ± 0,23 h nach oraler Gabe von 15 mg MTBE	Amberg et al. 2001
		Blut	–	–	35 min	Prah et al. 1994
			–	vierphasisch	1 min; 10 min; 1,5 h; 19 h	Nihlén et al. 1998
			–	–	1,8 ± 0,3 h nach Exposition gegen 4,5 ± 0,4 ppm MTBE für 4 h bzw. 2,6 ± 0,9 h nach Exposition gegen 38,7 ± 3,2 ppm MTBE für 4 h	Amberg et al. 1999
		Blut	–	triphasisch	0,7 ± 0,2 h; 1,2 ± 0,3 h; 3,7 ± 0,9 h nach oraler Gabe von 15 mg MTBE bzw. 0,8 ± 0,1 h; 1,8 ± 0,3 h; 8,1 ± 3,0 h nach oraler Gabe von 5 mg MTBE	Amberg et al. 2001
		Urin	–	linear, biphasisch	20 min; 3 h	Nihlén et al. 1998
			–	–	5,2 ± 1,0 h nach Exposition gegen 4,5 ± 0,4 ppm MTBE für 4 h bzw. 4,3 ± 1,4 h nach Exposition gegen 38,7 ± 3,2 ppm MTBE für 4 h	Amberg et al. 1999
			–	–	5,5 ± 2,0 h nach oraler Gabe von 15 mg MTBE bzw. 3,4 ± 0,9 h nach oraler Gabe von 5 mg MTBE	Amberg et al. 2001
	*tert‑*Butanol	Alveolarluft	–	linear	6,71 ± 2,17 h nach oraler Gabe von 15 mg MTBE	Amberg et al. 2001
		Blut	–	–	10 h	Nihlén et al. 1998
			–	–	6,5 ± 2,1 h nach Exposition gegen 4,5 ± 0,4 ppm MTBE für 4 h bzw. 5,3 ± 2,1 h nach Exposition gegen 38,7 ± 3,2 ppm MTBE für 4 h	Amberg et al. 1999
			–	linear	8,5 ± 2,4 h nach oraler Gabe von 15 mg MTBE bzw. 8,1 ± 1,6 h nach oraler Gabe von 5 mg MTBE	Amberg et al. 2001
		Urin	–	–	8,2 h	Nihlén et al. 1998
			–	–	12,0 ± 3 h nach Exposition gegen 4,5 ± 0,4 ppm MTBE für 4 h bzw. 10,4 ± 1,8 h nach Exposition gegen 38,7 ± 3,2 ppm MTBE für 4 h	Amberg et al. 1999
			–	–	8,1 ± 1,4 h nach oraler Gabe von 15 mg MTBE bzw. 7,7 ± 2,0 h nach oraler Gabe von 5 mg MTBE	Amberg et al. 2001
4-Methylpentan-2‑on (Methylisobutylketon)	4-Methylpentan-2‑on	Blut	–	biphasisch	12 min (0–30 min nach Exposition); 71 min (60–180 min nach Exposition)	Wigaeus Hjelm et al. 1990
		Urin	–	biphasisch	≈ 40 min; 6,9 h	Ogata et al. 1995 nach ACGIH 2010 a
2‑Propanol (Isopropanol)	2‑Propanol	Blut / Serum	–	erster Ordnung	3–6,4 h (akute Vergiftung)	Lacouture et al. 1983; Natowicz et al. 1985
		Blut	–	linear, erster Ordnung	2,5–3 h	Bohn et al. 1987; Daniel et al. 1981
	Aceton	Blut / Serum	–	erster Ordnung	22,4–24 h (akute Vergiftung)	Hawley und Falko 1982; Natowicz et al. 1985
Styrol	Styrol	Ausatemluft	–	biphasisch	13–52 min; 4–20 h	ACGIH 2015
		Blut	–	biphasisch	0,58 ± 0,08 h; 13,0 ± 0,8 h	Ramsey et al. 1980
		Urin	–	–	20 h	Prieto et al. 2002
Tetrachlorethen	Tetrachlorethen	Ausatemluft	–	biphasisch	< 3 h; 65 h	Stewart et al. 1970 nach ACGIH 2009
			–	–	3 d (mathematisches Modell, terminale Phase)	Guberan und Fernandez 1974
			–	triphasisch	3–10 min; 25–60 min; 210–220 min	Chien 1997
		Blut	–	triphasisch	15 min; 4 h; 4 d (mathematisches Modell)	Guberan und Fernandez 1974
			–	triphasisch	12–16 h; 30–40 h; 55–65 h	Monster et al. 1979
	Trichloressigsäure	Blut	–	–	50–100 h	Müller et al. 1974; Triebig et al. 1976
Tetrachlormethan (Tetrachlorkohlenstoff)	Tetrachlormethan	Alveolarluft	–	exponentiell	2,7 h nach Exposition gegen 10 ppm Tetrachlormethan für 3 h	Stewart et al. 1961
Tetrahydrofuran	Tetrahydrofuran	Alveolarluft	–	exponentiell	32 ± 12,7 min	Kageyama 1988 nach ACGIH 2008 a
		Urin	–	monophasisch	2,5 h	Kageyama 1988 nach ACGIH 2008 a
			–	monophasisch	118 min	JSOH 2014
			–	biphasisch	0,9–1,2 h; 4–5 h	Jones 2023
Toluol	Toluol	Ausatemluft	–	triphasisch	0,4 h; 3,9 h; 39 h	Pierce et al. 2004 nach ACGIH 2010 b
		Alveolarluft	–	exponentiell	17,5–20,8 h (30–120 h nach unfallartigem Ereignis)	Brugnone et al. 1983
			–	–	3,8 h (2,6–6 h)	Brugnone et al. 1986
		Blut	–	exponentiell	17,1–27,1 h (30–120 h nach unfallartigem Ereignis)	Brugnone et al. 1983
			–	–	4,5 h (3–6,2 h)	Brugnone et al. 1986
			–	triphasisch	3 min, 40 min; 738 min	Löf et al. 1993
			–	biphasisch	0,5 h; 1,94 h	Knecht et al. 2000
			Expositionsende	triphasisch	0,1–0,7 h; 1–12 h; 15–39 h	Pierce et al. 2004 nach ACGIH 2010 b
		Urin	3 h	exponentiell, biphasisch	≈ 0,5 h; 5 h	Ducos et al. 2008
			–	exponentiell, biphasisch	0,88 h; 12,9 h	Janasik et al. 2008
1,1,1‑Trichlorethan	1,1,1‑Trichlorethan	Ausatemluft	–	triphasisch	9 h; 20 h; 26 h (bis 100 h nach Expositionsende)	Monster et al. 1979
		Blut	–	triphasisch	9 h; 20 h; 26 h (bis 100 h nach Expositionsende)	Monster et al. 1979
			–	triphasisch	44 min; 5,7 h; 53 h	Nolan et al. 1984
			–	monoexponentiell (ab 30 h nach Expositionsende)	40 h (ab 30 h nach Expositionsende)	Bolt 1983
Trichlorethen	Trichlorethen	Ausatemluft	–	exponentiell	25 h (30–80 h nach Expositionsende)	Stewart et al. 1970 b nach Ikeda und Imanura 1973
			Expositionsende	exponentiell, mehrphasisch	–	Müller et al. 1974
		Blut	Expositionsende	exponentiell, mehrphasisch	–	Müller et al. 1974
			–	triphasisch	20 min; 3 h; 30 h	Fernández et al. 1975 nach ACGIH 2008 b
			–	–	21,7 h (17,3–24,3 h) (akute Vergiftung)	Kostrzewski et al. 1993
	Trichloressigsäure	Blut	–	–	50–100 h	Müller et al. 1974; Triebig et al. 1976
1,3,5‑Trimethylbenzol (Mesitylen)	1,3,5‑Trimethylbenzol	Urin	–	exponentiell, biphasisch	0,45 h; 6,7 h	Janasik et al. 2008
Xylol	Xylol	Ausatemluft	–	biphasisch	1 h; 20 h	Åstrand et al. 1978; Šedivec und Flek 1976
		Alveolarluft	–	triphasisch	0,8 h; 7,7 h; 17,3 h	Riihimäki et al. 1979
		Blut	–	mehrphasisch	t_1_: 0,5 h	Åstrand et al. 1978
			–	biphasisch	0,48 h; 1,82 h	Knecht et al. 2000
		Urin	–	biphasisch	0,84 h; 10,9 h	Janasik et al. 2008

Abkürzungen siehe Abkürzungsverzeichnis

**Tab.2:** Von der Kommission publizierte Headspace-Methoden für die Matrix Urin

Arbeitsstoff (Synonym)	Analyt	Multimethode (Anzahl Analyten)	Nachweisgrenze [μg/l]	Bestimmungsgrenze [μg/l]	Analysenmethode	Literatur
**Aromatische Kohlenwasserstoffe**						
Benzol	Benzol	ja (8)	0,007	0,021	dynamische HS‑GC‑MS	Van Pul et al. 2018
Ethylbenzol	Ethylbenzol		0,010	0,030		
Isopropylbenzol (Cumol)	Isopropylbenzol		0,012	0,036		
Styrol	Styrol		0,014	0,042		
Toluol	Toluol		0,029	0,087		
*m*‑Xylol	*m*‑Xylol		0,011	0,033		
*o*‑Xylol	*o‑*Xylol		0,015	0,045		
*p*‑Xylol	*p*‑Xylol		0,011	0,033		
**Halogenierte Kohlenwasserstoffe**						
Brommethan (Methylbromid)	Ameisensäure	–	200	n. a.	HS-GC-FID	Angerer und Schaller 1980
Halothan (2-Brom-2‑chlor-1,1,1‑trifluorethan)	Trifluoressigsäure	–	< 10	n. a.	HS-GC-ECD	Dallmeier und Müller 1982
1,1,2,2-Tetrachlorethan	Trichloressigsäure	ja (4)	10	30	HS-GC-MS	Will et al. 2017
Tetrachlorethen	Trichloressigsäure		10	30		
1,1,1-Trichlorethan	Trichloressigsäure		10	30		
Trichlorethen	Trichloressigsäure		10	30		
1-Brompropan	1‑Brompropan	ja (2)	0,01	0,03	dynamische HS‑GC‑MS	Roßbach et al. 2019
2-Brompropan	2‑Brompropan		0,01	0,04		
**Alkohole, Aldehyde, Ketone und Ether**						
Aceton	Ameisensäure	–	200	n. a.	HS‑GC‑FID	Angerer und Schaller 1980
Methanol	Ameisensäure	–	200	n. a.		
Aceton	Aceton	–	10 000	n. a.	HS‑GC‑FID	Machata und Eben 1980
Aceton	Aceton	ja (11)	100	n. a.	HS‑GC‑FID	Angerer et al. 1996
1‑Butanol	1‑Butanol		300	n. a.		
2‑Butanol	2‑Butanol		200	n. a.		
2‑Butanon (Methylethylketon)	2‑Butanon		80	n. a.		
Ethanol	Ethanol		800	n. a.		
2‑Hexanon	2‑Hexanon		30	n. a.		
Isobutanol (2-Methyl-1-propanol)	Isobutanol		200	n. a.		
Methanol	Methanol		600	n. a.		
Methylformiat	Methanol		600	n. a.		
4-Methylpentan-2‑on (Methylisobutylketon)	4-Methylpentan-2‑on		30	n. a.		
1‑Propanol	1‑Propanol		400	n. a.		
2‑Propanol (Isopropanol)	2‑Propanol		400	n. a.		
2‑Propanol (Isopropanol)	Aceton		100	n. a.		
Tetrahydrofuran	Tetrahydrofuran	–	100	300	HS‑GC‑FID	Blaszkewicz und Angerer 2012
Methyl-*tert*‑butylether (2-Methoxy-2-methylpropan)	Methyl-*tert*‑butylether	–	1,8	6	HS‑GC‑MS	Hoppe et al. 2018
Aceton	Aceton	ja (27)	10	30	HS‑GC‑MS	Göen et al. 2020
1‑Butanol	1‑Butanol		100	300		
2‑Butanol	2‑Butanol		50	150		
*tert*‑Butanol	*tert*‑Butanol		50	150		
2‑Butanon (Methylethylketon)	2‑Butanon		10	30		
Cyclohexanon	Cyclohexanon		50	150		
Cyclopentanon	Cyclopentanon		50	150		
3,3‑Dimethyl-2‑butanon (Methyl‑*tert*‑butylketon)	3,3‑Dimethyl-2‑butanon		10	30		
1,4‑Dioxan	1,4‑Dioxan		100	300		
Ethanol	Ethanol		100	300		
2‑Heptanon	2‑Heptanon		10	30		
3‑Heptanon	3‑Heptanon		10	30		
4‑Heptanon	4‑Heptanon		10	30		
2‑Hexanon	2‑Hexanon		10	30		
3‑Hexanon	3‑Hexanon		10	30		
Isobutanol (2-Methyl-1-propanol)	Isobutanol		50	150		
Methanol	Methanol		200	600		
3‑Methyl-2‑butanon (Methylisopropylketon)	3‑Methyl-2‑butanon		10	30		
Methyl-*tert*‑butylether (2-Methoxy-2-methylpropan)	Methyl-*tert*‑butylether		5	15		
Methyl-*tert*‑butylether (2-Methoxy-2-methylpropan)	*tert*‑Butanol		50	150		
4-Methylpentan-2‑on (Methylisobutylketon)	4-Methylpentan-2‑on		10	30		
2‑Pentanon	2‑Pentanon		20	60		
3‑Pentanon	3‑Pentanon		20	60		
1‑Propanol	1‑Propanol		30	90		
2‑Propanol (Isopropanol)	2‑Propanol		20	60		
2‑Propanol (Isopropanol)	Aceton		10	30		
Tetrahydrofuran	Tetrahydrofuran		10	30		

Abkürzungen siehe Abkürzungsverzeichnis

**Tab.3:** Von der Kommission publizierte Headspace-Methoden für die Matrix Blut

Arbeitsstoff (Synonym)	Analyt	Multimethode (Anzahl Analyten)	Nachweisgrenze [μg/l] (sofern nicht anders angegeben)	Bestimmungsgrenze [μg/l]	Analysenmethode	Literatur
**Aromatische Kohlenwasserstoffe**						
Styrol	Styrol	–	50	n. a.	HS-GC-FID	Schaller et al. 1980
Benzol	Benzol	ja (6)	20	n. a.	HS-GC-FID	Knecht und Angerer 1983
Ethylbenzol	Ethylbenzol		20	n. a.		
Toluol	Toluol		40	n. a.		
*m*‑Xylol	*m*‑Xylol		40	n. a.		
*o‑*Xylol	*o‑*Xylol		40	n. a.		
*p*‑Xylol	*p*‑Xylol		40	n. a.		
Isopropylbenzol (Cumol)	Isopropylbenzol	–	86	n. a.	HS-GC-FID	Goenechea und Machata 1983
Benzol	Benzol	ja (5)	3	n. a.	HS-GC-FID	Angerer et al. 1994
Ethylbenzol	Ethylbenzol		8	n. a.		
Toluol	Toluol		5	n. a.		
*m‑*Xylol	*m‑*Xylol		8	n. a.		
*o*‑Xylol	*o*‑Xylol		8	n. a.		
Benzol	Benzol	ja (14)	0,7	2,1	HS-GC-MS	Göen et al. 2018
Chlorbenzol	Chlorbenzol		0,9	2,7		
Ethylbenzol	Ethylbenzol		0,9	2,7		
Isopropylbenzol (Cumol)	Isopropylbenzol		1,0	3,0		
1‑Propylbenzol	1‑Propylbenzol		1,0	3,0		
Styrol	Styrol		1,0	3,0		
1,2,3,5-Tetramethylbenzol (Isodurol)	1,2,3,5-Tetramethylbenzol		3,0	9,0		
Toluol	Toluol		0,7	2,1		
1,2,3-Trimethylbenzol (Hemimellitol)	1,2,3-Trimethylbenzol		1,5	4,5		
1,2,4-Trimethylbenzol (Pseudocumol)	1,2,4-Trimethylbenzol		1,5	4,5		
1,3,5-Trimethylbenzol (Mesitylen)	1,3,5-Trimethylbenzol		1,5	4,5		
*m‑*Xylol	*m*‑Xylol		0,9	2,7		
*o*‑Xylol	*o*‑Xylol		0,9	2,7		
*p*‑Xylol	*p*‑Xylol		0,9	2,7		
**Halogenierte Kohlenwasserstoffe**						
Halothan (2-Brom-2-chlor-1,1,1‑trifluorethan)	Halothan	–	50	n. a.	HS-GC-ECD	Schaller et al. 1978
1,1,1,2‑Tetrachlorethan	Trichloressigsäure	–	200	n. a.	HS-GC-ECD	Angerer und Eben 1980
Tetrachlorethen	Trichloressigsäure	–	200	n. a.		
Trichlorethen	Trichloressigsäure	–	200	n. a.		
1,1‑Dichlorethan	1,1‑Dichlorethan	–	100	n. a.	HS‑GC‑FID	Zorn et al. 1982
1,2‑Dichlorethan	1,2‑Dichlorethan	–	82	n. a.	HS-GC-FID	Angerer et al. 1981
1,1,2‑Trichlor-1,2,2‑trifluorethan^a)^	1,1,2‑Trichlor-1,2,2‑trifluorethan^a)^	–	100	n. a.	HS-GC-ECD	Schaller et al. 1982 a
Trichlorethen	Trichlorethen	–	50	n. a.	HS-GC-ECD	Schaller et al. 1982 b
Trifluoressigsäure	Trifluoressigsäure	–	< 10	n. a.	HS-GC-ECD	Dallmeier und Müller 1982
Dichlormethan (Methylenchlorid)	Dichlormethan	ja (4)	50	n. a.	HS-GC-ECD	Angerer und Zorn 1982
Tetrachlorethen	Tetrachlorethen		1,2	n. a.		
Tetrachlormethan (Tetrachlorkohlenstoff)	Tetrachlormethan		0,5	n. a.		
Trichlorethen	Trichlorethen		1,5	n. a.		
1,1,2-Trichlorethan	1,1,2-Trichlorethan	–	200	n. a.	HS-GC-ECD	Eben et al. 1983
1,2-Dichlorethen	1,2-Dichlorethen	ja (8)	55	n. a.	HS-GC-ECD	Angerer et al. 1991
Dichlormethan (Methylenchlorid)	Dichlormethan		25	n. a.		
Halothan (2‑Brom-2‑chlor-1,1,1‑trifluorethan)	Halothan		0,2	n. a.		
Tetrachlorethen	Tetrachlorethen		0,5	n. a.		
Tetrachlormethan (Tetrachlorkohlenstoff)	Tetrachlormethan		0,3	n. a.		
1,1,1‑Trichlorethan	1,1,1‑Trichlorethan		1,0	n. a.		
Trichlorethen	Trichlorethen		1,1	n. a.		
Trichlormethan (Chloroform)	Trichlormethan		0,8	n. a.		
1,2‑Dichlorethan	1,2‑Dichlorethan	ja (7)	0,1	0,3	HS-GC-MS	Göen et al. 2021
Dichlormethan (Methylenchlorid)	Dichlormethan		1,0	3,0		
Tetrachlorethen	Tetrachlorethen		0,1	0,3		
Tetrachlormethan (Tetrachlorkohlenstoff)	Tetrachlormethan		0,1	0,3		
1,1,1‑Trichlorethan	1,1,1‑Trichlorethan		0,1	0,3		
Trichlorethen	Trichlorethen		0,1	0,3		
Trichlormethan (Chloroform)	Trichlormethan		0,8	2,4		
**Alkohole, Aldehyde, Ketone und Ether**						
2-Hexanol	2-Hexanol	–	500	n. a.	HS-GC-FID	Eben und Barchet 1981
2-Hexanon	2-Hexanol	–	500	n. a.		
2-Hexanon	2-Hexanon	–	500	n. a.	HS-GC-FID	Eben und Pilz 1978
Aceton	Aceton	–	10 000	n. a.	HS-GC-FID	Machata und Eben 1980
1*‑*Butanol	1*‑*Butanol	–	250	n. a.	HS-GC-FID	Angerer und Möller 1980
Cyclohexanon	Cyclohexanon	–	750	n. a.	HS-GC-FID	Angerer und Eben 1981
1,4-Dioxan	1,4-Dioxan	–	2000	n. a.	HS-GC-FID	Eben und Machata 1981
Aceton	Aceton	ja (11)	200	n. a.	HS-GC-FID	Angerer et al. 1996
1-Butanol	1-Butanol		800	n. a.		
2-Butanol	2-Butanol		400	n. a.		
2-Butanon (Methylethylketon)	2-Butanon		100	n. a.		
Ethanol	Ethanol		1300	n. a.		
2-Hexanon	2-Hexanon		70	n. a.		
Isobutanol (2-Methyl-1‑propanol)	Isobutanol		400	n. a.		
Methanol	Methanol		600	n. a.		
4-Methylpentan-2‑on (Methylisobutylketon)	4-Methylpentan-2‑on		50	n. a.		
1-Propanol	1-Propanol		800	n. a.		
2‑Propanol (Isopropanol)	2‑Propanol		600	n. a.		
2‑Propanol (Isopropanol)	Aceton		200	n. a.		
Methyl-*tert*‑butylether (2‑Methoxy-2‑methylpropan)	Methyl-*tert*‑butylether	–	1,2	4	HS-GC-MS	Hoppe et al. 2018
**Sonstige**						
*n*‑Hexan	2-Hexanol	–	500	n. a.	HS-GC-FID	Eben und Barchet 1981
Kohlenstoffdisulfid (Schwefelkohlenstoff)	Kohlenstoffdisulfid	–	50	n. a.	HS-GC-ECD	Eben und Barchet 1983
Kohlenmonoxid	Kohlenmonoxid nach katalytischer Umwandlung zu Methan	–	0,17 % CO‑Hb	n. a.	HS-GC‑FID	Angerer und Zorn 1985
Cyanid	Cyanwasserstoff	–	70 (gepackte Säule); 100 (Kapillarsäule)	n. a.	HS-GC mit thermoionischem Stickstoffdetektor	Eben und Lewalter 1988
Cyanidbildner						
Cyanwasserstoff						
Natrium-/Kaliumcyanid						
Methylquecksilber	Methylquecksilber	–	0,4	n. a.	HS-GC-MS	Hoppe und Heinrich-Ramm 2006

^a)^ Matrix: Serum

Abkürzungen siehe Abkürzungsverzeichnis

**Tab.4:** Von der Kommission publizierte Headspace-Methoden für die Matrix Ausatemluft

Arbeitsstoff	Analyt	Multimethode (Anzahl Analyten)	Nachweisgrenze [μg/l]	Bestimmungsgrenze [μg/l]	Analysenmethode	Literatur
**Alkohole, Aldehyde, Ketone und Ether**						
Furan	Furan	–	0,00002	0,00006	HS-SPME-GC-MS/MS	Ziener et al. 2024

Abkürzung siehe Abkürzungsverzeichnis

**Tab.5:** Weitere im internationalen Schrifttum publizierte Headspace-Methoden für die Matrix Urin

Analyt (Synonym)	Multimethode (Anzahl Analyten)	Nachweisgrenze [μg/l]	Bestimmungsgrenze [μg/l]	Analysenmethode	Literatur
**Aromatische Kohlenwasserstoffe**					
Acenaphthen	ja (13)	0,002	0,006	HS-SPME-GC-MS	Campo et al. 2009
Acenaphthylen	ja (13)	0,001	0,004	HS-SPME-GC-MS	Campo et al. 2009
Anthracen	ja (13)	0,001	0,002	HS-SPME-GC-MS	Campo et al. 2009
Benzo[*a*]anthracen	ja (13)	0,002	0,005	HS-SPME-GC-MS	Campo et al. 2009
Benzo[*b*]fluoranthen	ja (13)	0,005	0,016	HS-SPME-GC-MS	Campo et al. 2009
Benzo[*k*]fluoranthen	ja (13)	0,006	0,020	HS-SPME-GC-MS	Campo et al. 2009
Benzol	ja (6)	0,025	n. a.	HS-SPME-GC-MS	Fustinoni et al. 1999
	ja (6)	0,005	n. a.	HS-SPME-GC-MS	Andreoli et al. 1999
	ja (4)	0,013	n. a.	statische HS-GC-MS	Perbellini et al. 2002
	ja (3)	0,010	n. a.	statische HS-GC-MS	Perbellini et al. 2003
	ja (6)	0,025	n. a.	PT-HS-GC-PID	Brčić Karačonji und Skender 2007
	ja (6)	0,05	n. a.	HS-SPME-GC-MS	Brčić Karačonji und Skender 2007
	ja (6)	0,015	n. a.	HS-SPME-GC-MS	Fustinoni et al. 2010
	ja (15)	0,3	1	HS-SPME-GC-MS	Song et al. 2017
	ja (5)	0,02	0,07	HS-SPME-GC-FID	Tajik et al. 2017
	ja (5)	0,04	n. a.	HS-SPME-GC-FID	Yousefi et al. 2018
	ja (11)	n. a.	0,010	dynamische HS-GC-MS	Erb et al. 2019
	ja (5)	0,42	1,40	HS-NTD-GC-FID	Saedi et al. 2020
Benzo[*a*]pyren	ja (13)	0,005	0,015	HS-SPME-GC-MS	Campo et al. 2009
*n-*Butylbenzol	ja (15)	0,6	2	HS-SPME-GC-MS	Song et al. 2017
*sec*-Butylbenzol	ja (15)	0,6	2	HS-SPME-GC-MS	Song et al. 2017
*tert*-Butylbenzol	ja (15)	0,6	2	HS-SPME-GC-MS	Song et al. 2017
Chrysen	ja (13)	n. a.	0,005	HS-SPME-GC-MS	Campo et al. 2009
Ethylbenzol	ja (6)	0,012	n. a.	HS-SPME-GC-MS	Fustinoni et al. 1999
	ja (6)	0,01	n. a.	HS-SPME-GC-MS	Andreoli et al. 1999
	ja (4)	0,017	n. a.	statische HS-GC-MS	Perbellini et al. 2002
	ja (6)	0,035	n. a.	PT-HS-GC-PID	Brčić Karačonji und Skender 2007
	ja (6)	0,035	n. a.	HS-SPME-GC-MS	Brčić Karačonji und Skender 2007
	ja (6)	0,015	n. a.	HS-SPME-GC-MS	Fustinoni et al. 2010
	ja (15)	0,3	1	HS-SPME-GC-MS	Song et al. 2017
	ja (5)	0,06	0,2	HS-SPME-GC-FID	Tajik et al. 2017
	ja (5)	0,06	n. a.	HS-SPME-GC-FID	Yousefi et al. 2018
	ja (11)	n. a.	0,010	dynamische HS-GC-MS	Erb et al. 2019
	ja (5)	0,22	0,73	HS-NTD-GC-FID	Saedi et al. 2020
Fluoranthen	ja (13)	n. a.	0,00426	HS-SPME-GC-MS	Campo et al. 2009
Fluoren	ja (13)	n. a.	0,00462	HS-SPME-GC-MS	Campo et al. 2009
Isopropylbenzol (Cumol)	ja (15)	0,6	2	HS-SPME-GC-MS	Song et al. 2017
*m*-Kresol	ja (2)	7,0	n. a.	HS-SPME-GC-MS	Fustinoni et al. 2005
(*m *+ *p*)-Kresol	ja (15)	0,3	1	HS-SPME-GC-MS	Song et al. 2017
*o*-Kresol	ja (2)	6,0	n. a.	HS-SPME-GC-MS	Fustinoni et al. 2005
	ja (15)	0,3	1	HS-SPME-GC-MS	Song et al. 2017
Naphthalin	ja (13)	n. a.	0,023	HS-SPME-GC-MS	Campo et al. 2009
	ja (6)	0,025	n. a.	HS-SPME-GC-MS	Fustinoni et al. 2010
	ja (15)	0,3	1	HS-SPME-GC-MS	Song et al. 2017
Phenanthren	ja (13)	n. a.	0,005	HS-SPME-GC-MS	Campo et al. 2009
*n*-Propylbenzol	ja (15)	0,6	2	HS-SPME-GC-MS	Song et al. 2017
Pyren	ja (13)	n. a.	0,004	HS-SPME-GC-MS	Campo et al. 2009
Styrol	ja (11)	n. a.	0,050	dynamische HS-GC-MS	Erb et al. 2019
Toluol	ja (6)	0,034	n. a.	HS-SPME-GC-MS	Fustinoni et al. 1999
	ja (6)	0,005	n. a.	HS-SPME-GC-MS	Andreoli et al. 1999
	ja (4)	0,013	n. a.	statische HS-GC-MS	Perbellini et al. 2002
	ja (6)	0,015	n. a.	PT-HS-GC-PID	Brčić Karačonji und Skender 2007
	ja (6)	0,039	n. a.	HS-SPME-GC-MS	Brčić Karačonji und Skender 2007
	ja (6)	0,015	n. a.	HS-SPME-GC-MS	Fustinoni et al. 2010
	ja (18)	1000	n. a.	statische HS-GC-FID-MS	Tiscione et al. 2013
	ja (4)	1,63	5,44	HS-GC-FID	Muna und Pereira 2016
	ja (15)	0,3	1	HS-SPME-GC-MS	Song et al. 2017
	ja (2)	0,5	n. a.	HS-Cryotrapping-GC-MS	Jeong et al. 2017
	ja (5)	0,02	0,07	statische HS-GC-MS	Paredes et al. 2017
	ja (5)	0,03	0,1	HS-SPME-GC-FID	Tajik et al. 2017
	ja (5)	0,03	n. a.	HS-SPME-GC-FID	Yousefi et al. 2018
	ja (11)	n. a.	0,010	dynamische HS-GC-MS	Erb et al. 2019
	ja (5)	0,35	1,18	HS-NTD-GC-FID	Saedi et al. 2020
*m*-Xylol	ja (4)	0,013	n. a.	statische HS-GC-MS	Perbellini et al. 2002
	ja (11)	n. a.	0,010	dynamische HS-GC-MS	Erb et al. 2019
(*m *+ *p*)-Xylol	ja (6)	0,023	n. a.	HS-SPME-GC-MS	Fustinoni et al. 1999
	ja (6)	0,01	n. a.	HS-SPME-GC-MS	Andreoli et al. 1999
	ja (6)	0,026	n. a.	PT-HS-GC-PID	Brčić Karačonji und Skender 2007
	ja (6)	0,042	n. a.	HS-SPME-GC-MS	Brčić Karačonji und Skender 2007
	ja (6)	0,015	n. a.	HS-SPME-GC-MS	Fustinoni et al. 2010
	ja (15)	0,3	1	HS-SPME-GC-MS	Song et al. 2017
	ja (5)	0,05	n. a.	HS-SPME-GC-FID	Yousefi et al. 2018
	ja (5)	0,10	0,32	HS-NTD-GC-FID	Saedi et al. 2020
*o*-Xylol	ja (6)	0,015	n. a.	HS-SPME-GC-MS	Fustinoni et al. 1999
	ja (6)	0,01	n. a.	HS-SPME-GC-MS	Andreoli et al. 1999
	ja (6)	0,030	n. a.	PT-HS-GC-PID	Brčić Karačonji und Skender 2007
	ja (6)	0,042	n. a.	HS-SPME-GC-MS	Brčić Karačonji und Skender 2007
	ja (6)	0,015	n. a.	HS-SPME-GC-MS	Fustinoni et al. 2010
	ja (15)	0,3	1	HS-SPME-GC-MS	Song et al. 2017
	ja (5)	0,07	0,2	HS-SPME-GC-FID	Tajik et al. 2017
	ja (5)	0,05	n. a.	HS-SPME-GC-FID	Yousefi et al. 2018
	ja (11)	n. a.	0,010	dynamische HS-GC-MS	Erb et al. 2019
	ja (5)	0,55	1,84	HS-NTD-GC-FID	Saedi et al. 2020
*p*-Xylol	ja (5)	0,01	0,05	statische HS-GC-MS	Paredes et al. 2017
	ja (5)	0,05	0,2	HS-SPME-GC-FID	Tajik et al. 2017
	ja (11)	n. a.	0,015	dynamische HS-GC-MS	Erb et al. 2019
**Aliphatische Kohlenwasserstoffe**					
1,3-Butadien	ja (3)	0,001	n. a.	statische HS-GC-MS	Perbellini et al. 2003
**Halogenierte Kohlenwasserstoffe**					
1-Brompropan	ja (2)	2,0	n. a.	statische HS-GC-ECD	B’Hymer und Cheever 2005
2-Brompropan	ja (2)	7,0	n. a.	statische HS-GC-ECD	B’Hymer und Cheever 2005
Chlordifluormethan (Freon-22)	ja (18)	5000	n. a.	statische HS-GC-FID-MS	Tiscione et al. 2013
Chlorethan	ja (18)	1900	n. a.	statische HS-GC-FID-MS	Tiscione et al. 2013
Dibromchlormethan	ja (6)	0,001	n. a.	TLHS-DAI-GC-ECD	Polkowska et al. 1999
Dichlordifluormethan (Freon-12)	ja (18)	5000	n. a.	statische HS-GC-FID-MS	Tiscione et al. 2013
Dichlorfluormethan (Freon-21)	ja (18)	5000	n. a.	statische HS-GC-FID-MS	Tiscione et al. 2013
Dichlormethan (Methylenchlorid)	ja (6)	0,001	n. a.	TLHS-DAI-GC-ECD	Polkowska et al. 1999
	ja (3)	0,005	n. a.	HS-SPME-GC-MS	Poli et al. 2005
	ja (4)	25,75	85,83	HS-GC-FID	Muna und Pereira 2016
	ja (11)	n. a.	0,015	dynamische HS-GC-MS	Erb et al. 2019
1,2-Dichlortetrafluorethan (Freon-114)	ja (18)	5000	n. a.	statische HS-GC-FID-MS	Tiscione et al. 2013
1,1-Difluorethan	ja (18)	< 2600	n. a.	statische HS-GC-FID-MS	Tiscione et al. 2013
Dimethyldisulfid	ja (5)	0,48	1,43	statische HS-GC-MS	Paredes et al. 2017
Fluortrichlormethan (Freon-11)	ja (18)	5000	n. a.	statische HS-GC-FID-MS	Tiscione et al. 2013
Tetrachlorethen	ja (6)	0,001	n. a.	TLHS-DAI-GC-ECD	Polkowska et al. 1999
	ja (3)	0,005	n. a.	HS-SPME-GC-MS	Poli et al. 2005
	ja (11)	n. a.	0,010	dynamische HS-GC-MS	Erb et al. 2019
Tetrachlormethan (Tetrachlorkohlenstoff)	ja (6)	0,001	n. a.	TLHS-DAI-GC-ECD	Polkowska et al. 1999
1,1,1,2-Tetrafluorethan	ja (18)	20 000	n. a.	statische HS-GC-FID-MS	Tiscione et al. 2013
Tribrommethan (Bromoform)	ja (6)	0,001	n. a.	TLHS-DAI-GC-ECD	Polkowska et al. 1999
Trichloressigsäure	–	n. a.	9,0	PT-HS-GC-MS	Johns et al. 2005
	–	n. a.	110	HS-GC-TCD	Xie et al. 2018
	–	n. a.	172	HS-GC-FID	Xie et al. 2018
Trichlorethen	ja (3)	0,005	n. a.	HS-SPME-GC-MS	Poli et al. 2005
	ja (11)	n. a.	0,010	dynamische HS-GC-MS	Erb et al. 2019
Trichlormethan (Chloroform)	ja (6)	0,001	n. a.	TLHS-DAI-GC-ECD	Polkowska et al. 1999
	ja (11)	n. a.	0,010	dynamische HS-GC-MS	Erb et al. 2019
1,1,1-Trifluorethan (Freon-143a)	ja (18)	3400	n. a.	statische HS-GC-FID-MS	Tiscione et al. 2013
**Alkohole, Aldehyde, Ketone und Ether**					
Acetaldehyd	ja (7)	15 667	47 000	HS-GC-FID	Kovatsi et al. 2011
	ja (18)	18 750	n. a.	statische HS-GC-FID-MS	Tiscione et al. 2013
	ja (12)	0,002	n. a.	statische HS-GC-MS	Serrano et al. 2016
Aceton	ja (7)	24 333	73 000	HS-GC-FID	Kovatsi et al. 2011
	ja (18)	25 000	n. a.	statische HS-GC-FID-MS	Tiscione et al. 2013
*tert*-Amylmethylether	ja (3)	0,006	n. a.	HS-SPME-GC-MS	Scibetta et al. 2007
Benzaldehyd	ja (44)	0,013	0,042	HS-SPME-GC-IT/MS	Calejo et al. 2016
Butanal	ja (44)	0,835	2,78	HS-SPME-GC-IT/MS	Calejo et al. 2016
	ja (12)	0,003	n. a.	statische HS-GC-MS	Serrano et al. 2016
2,3-Butandion (Diacetyl)	ja (44)	0,263	0,878	HS-SPME-GC-IT/MS	Calejo et al. 2016
1-Butanol	ja (18)	25 000	n. a.	statische HS-GC-FID-MS	Tiscione et al. 2013
2-Butanon (Methylethylketon)	ja (44)	0,801	2,67	HS-SPME-GC-IT/MS	Calejo et al. 2016
	–	4,2	21,6	HS-SPME-GC-FID	Chou et al. 1999
	ja (18)	5000	n. a.	statische HS-GC-FID-MS	Tiscione et al. 2013
Butenal (Crotonaldehyd)	ja (44)	0,013	0,043	HS-SPME-GC-IT/MS	Calejo et al. 2016
	ja (12)	0,003	n. a.	statische HS-GC-MS	Serrano et al. 2016
Cyclohexanon	ja (44)	0,137	0,455	HS-SPME-GC-IT/MS	Calejo et al. 2016
*trans*,*trans*-2,4-Decadienal	ja (44)	0,046	0,152	HS-SPME-GC-IT/MS	Calejo et al. 2016
Decanal	ja (44)	0,011	0,036	HS-SPME-GC-IT/MS	Calejo et al. 2016
2-Decanon	ja (44)	0,245	0,815	HS-SPME-GC-IT/MS	Calejo et al. 2016
*trans*-2-Decenal	ja (44)	0,014	0,046	HS-SPME-GC-IT/MS	Calejo et al. 2016
2,6-Dimethyl-7-octen-2-ol (Dihydromyrcenol)	ja (5)	0,03	0,08	statische HS-GC-MS	Paredes et al. 2017
Ethanol	ja (7)	21 667	65 000	HS-GC-FID	Kovatsi et al. 2011
	ja (2)	210	n. a.	HS-Cryotrapping-GC-MS	Jeong et al. 2017
Ethyl‑*tert*‑butylether	ja (3)	0,006	n. a.	HS-SPME-GC-MS	Scibetta et al. 2007
	ja (6)	0,015	n. a.	HS-SPME-GC-MS	Fustinoni et al. 2010
Formaldehyd	ja (12)	0,001	n. a.	statische HS-GC-MS	Serrano et al. 2016
Glyoxal	ja (44)	0,068	0,226	HS-SPME-GC-IT/MS	Calejo et al. 2016
	ja (12)	0,015	n. a.	statische HS-GC-MS	Serrano et al. 2016
Heptanal	ja (44)	0,010	0,034	HS-SPME-GC-IT/MS	Calejo et al. 2016
	ja (12)	0,008	n. a.	statische HS-GC-MS	Serrano et al. 2016
	ja (2)	0,01	n. a.	HS-SPME-GC-FID	Ghaedrahmati et al. 2021
4-Heptanon	ja (44)	0,942	3,14	HS-SPME-GC-IT/MS	Calejo et al. 2016
*trans*-2-Heptenal	ja (44)	0,012	0,040	HS-SPME-GC-IT/MS	Calejo et al. 2016
*trans*,*trans*-2,4-Hexadienal	ja (44)	0,012	0,039	HS-SPME-GC-IT/MS	Calejo et al. 2016
Hexanal	ja (44)	0,065	0,217	HS-SPME-GC-IT/MS	Calejo et al. 2016
	ja (12)	0,006	n. a.	statische HS-GC-MS	Serrano et al. 2016
	ja (2)	0,001	n. a.	HS-SPME-GC-FID	Ghaedrahmati et al. 2021
2,5-Hexandion	–	25	75	HS-SPME-GC-FID	Oliveira et al. 2009
2-Hexanon	ja (44)	0,017	0,055	HS-SPME-GC-IT/MS	Calejo et al. 2016
*trans*-2-Hexenal	ja (44)	0,011	0,035	HS-SPME-GC-IT/MS	Calejo et al. 2016
4-Hydroxy-2-nonenal	ja (44)	15,0	50,0	HS-SPME-GC-IT/MS	Calejo et al. 2016
Isobutanol (2-Methyl-1-propanol)	ja (18)	50 000	n. a.	statische HS-GC-FID-MS	Tiscione et al. 2013
Malondialdehyd	ja (44)	0,025	0,083	HS-SPME-GC-IT/MS	Calejo et al. 2016
	ja (12)	0,010	n. a.	statische HS-GC-MS	Serrano et al. 2016
Methanol	ja (7)	29 000	87 000	HS-GC-FID	Kovatsi et al. 2011
	ja (18)	250 000	n. a.	statische HS-GC-FID-MS	Tiscione et al. 2013
1-Methoxy-2-propanol	–	100	n. a.	statische HS-GC-FID	Tomicic und Berode 2010
2-Methylbutanal	ja (44)	0,020	0,065	HS-SPME-GC-IT/MS	Calejo et al. 2016
3-Methylbutanal	ja (44)	0,019	0,063	HS-SPME-GC-IT/MS	Calejo et al. 2016
3-Methyl-1-butanol (Isopentanol)	ja (18)	25 000	n. a.	statische HS-GC-FID-MS	Tiscione et al. 2013
Methyl-*tert*‑butylether (2‑Methoxy-2‑methylpropan)	ja (3)	0,006	n. a.	HS-SPME-GC-MS	Scibetta et al. 2007
	ja (6)	0,010	n. a.	HS-SPME-GC-MS	Fustinoni et al. 2010
Methylglyoxal	ja (44)	0,025	0,083	HS-SPME-GC-IT/MS	Calejo et al. 2016
	ja (12)	0,010	n. a.	statische HS-GC-MS	Serrano et al. 2016
6-Methyl-5-heptanon	ja (44)	0,212	0,708	HS-SPME-GC-IT/MS	Calejo et al. 2016
4 Methylpentan-2-on (Methylisobutylketon)	ja (4)	68,86	229,54	HS-GC-FID	Muna und Pereira 2016
2-Methylpropanal (Isobutanal)	ja (44)	0,038	0,125	HS-SPME-GC-IT/MS	Calejo et al. 2016
2-Methylpropenal	ja (44)	0,199	0,663	HS-SPME-GC-IT/MS	Calejo et al. 2016
*trans*,*trans*-2,4-Nonadienal	ja (44)	0,010	0,034	HS-SPME-GC-IT/MS	Calejo et al. 2016
Nonanal	ja (44)	0,020	0,065	HS-SPME-GC-IT/MS	Calejo et al. 2016
2-Nonanon	ja (44)	0,039	0,129	HS-SPME-GC-IT/MS	Calejo et al. 2016
*trans*-2-Nonenal	ja (44)	0,020	0,067	HS-SPME-GC-IT/MS	Calejo et al. 2016
Octanal	ja (44)	0,152	0,507	HS-SPME-GC-IT/MS	Calejo et al. 2016
2-Octanon (Methylhexylketon)	ja (44)	0,107	0,355	HS-SPME-GC-IT/MS	Calejo et al. 2016
	ja (5)	0,06	0,17	statische HS-GC-MS	Paredes et al. 2017
*trans*-2-Octenal	ja (44)	0,022	0,072	HS-SPME-GC-IT/MS	Calejo et al. 2016
Pentanal	ja (44)	0,273	0,909	HS-SPME-GC-IT/MS	Calejo et al. 2016
	ja (12)	0,006	n. a.	statische HS-GC-MS	Serrano et al. 2016
2-Pentanon	ja (44)	0,013	0,043	HS-SPME-GC-IT/MS	Calejo et al. 2016
*trans*-2-Pentenal	ja (44)	0,040	0,133	HS-SPME-GC-IT/MS	Calejo et al. 2016
3-Penten-2-on	ja (44)	0,498	1,66	HS-SPME-GC-IT/MS	Calejo et al. 2016
Phenylacetaldehyd	ja (44)	0,009	0,029	HS-SPME-GC-IT/MS	Calejo et al. 2016
Propanal	ja (44)	0,016	0,052	HS-SPME-GC-IT/MS	Calejo et al. 2016
	ja (12)	0,004	n. a.	statische HS-GC-MS	Serrano et al. 2016
1-Propanol	ja (7)	26 000	78 000	HS-GC-FID	Kovatsi et al. 2011
2‑Propanol (Isopropanol)	ja (18)	100 000	n. a.	statische HS-GC-FID-MS	Tiscione et al. 2013
2-Propenal (Acrolein)	ja (44)	0,030	0,091	HS-SPME-GC-IT/MS	Calejo et al. 2016
	ja (12)	0,003	n. a.	statische HS-GC-MS	Serrano et al. 2016
Undecanal	ja (44)	0,011	0,035	HS-SPME-GC-IT/MS	Calejo et al. 2016
2-Undecanon	ja (44)	0,074	0,247	HS-SPME-GC-IT/MS	Calejo et al. 2016
**Inhalationsnarkotika**					
Brommethan als Metabolit des Halothans	ja (2)	2876–8789	n. a.	statische HS-GC-FID	Maiorino et al. 1980
Desfluran	ja (7)	13 667	41 000	HS-GC-FID	Kovatsi et al. 2011
Halothan (2-Brom-2-chlor-1,1,1‑trifluorethan)	ja (3)	0,02–0,03	n. a.	HS-SPME-GC-MS	Poli et al. 1999
	ja (3)	5	n. a.	statische HS- GC-MS	Poli et al. 1999
	ja (4)	0,05	0,15	statische HS-GC-MS	Accorsi et al. 2001
	–	≈ 4	≈ 50	HS-SPME-GC-MS	Musshoff et al. 2000
Hexafluorisopropanol als Metabolit des Sevoflurans	–	≈ 1	n. a.	HSSE-GC-MS	Accorsi et al. 2005
	–	n. a.	0,5	HS-GC-MS	Herzog-Niescery et al. 2020
Isofluran	ja (3)	0,15–0,02	n. a.	HS-SPME-GC-MS	Poli et al. 1999
	ja (3)	1	n. a.	statische HS-GC-MS	Poli et al. 1999
	ja (4)	0,02	0,08	statische HS-GC-MS	Accorsi et al. 2001
Lachgas (Distickstoffmonoxid)	ja (3)	0,075–0,1	n. a.	HS-SPME-GC-MS	Poli et al. 1999
	ja (3)	1	n. a.	statische HS-GC-MS	Poli et al. 1999
	ja (4)	0,3	1,0	statische HS-GC-MS	Accorsi et al. 2001
Sevofluran	ja (4)	0,03	0,10	statische HS-GC-MS	Accorsi et al. 2001
	–	≈ 1	n. a.	HSSE-GC-MS	Accorsi et al. 2005
	ja (7)	13 667	41 000	HS-GC-FID	Kovatsi et al. 2011
Trifluoressigsäure als Metabolit des Halothans, Isoflurans und Fluroxens	ja (2)	1140	n. a.	statische HS-GC-FID	Maiorino et al. 1980
**Sonstige**					
2,5-Dimethylfuran	ja (3)	0,005	n. a.	statische HS-GC-MS	Perbellini et al. 2003
2-Furfural	ja (44)	0,044	0,147	HS-SPME-GC-IT/MS	Calejo et al. 2016
Menthol	–	1,7	n. a.	HS-SPME-GC-MS	Huang et al. 2017
5-Methyl-2-furfural	ja (44)	0,025	0,083	HS-SPME-GC-IT/MS	Calejo et al. 2016
Tetrahydrofuran	ja (4)	155,12	517,07	HS-GC-FID	Muna und Pereira 2016

Abkürzungen siehe Abkürzungsverzeichnis

**Tab.6:** Weitere im internationalen Schrifttum publizierte Headspace-Methoden für die Matrices Blut, Serum und Plasma

Analyt (Synonym)	Multimethode (Anzahl Analyten)	Nachweisgrenze [μg/l]	Bestimmungsgrenze [μg/l]	Analysenmethode	Literatur
**Aromatische Kohlenwasserstoffe**					
Benzol	ja (6)	0,005	n. a.	HS-SPME-GC-MS	Andreoli et al. 1999
	ja (20)	n. a.	≈ 10	HS-SPME-GC-MS	Liu et al. 2000
	ja (4)	0,016	n. a.	statische HS-GC-MS	Perbellini et al. 2002
	ja (3)	0,010	n. a.	statische HS-GC-MS	Perbellini et al. 2003
	ja (31)	0,024	n. a.	HS-SPME-GC-MS	Blount et al. 2006
	ja (10)	0,4	1,2	HS-NTD-GC-MS	Alonso et al. 2012
	ja (70)	0,001	0,004	HS-SPME-GC-MS	Mochalski et al. 2013
	ja (24)	n. a.	7,21–10,6	HS-SPME-GC-MS	Waters et al. 2017
*n*‑Butylbenzol	ja (24)	n. a.	7,21–10,6	HS-SPME-GC-MS	Waters et al. 2017
*tert*‑Butylbenzol	ja (24)	n. a.	7,21–10,6	HS-SPME-GC-MS	Waters et al. 2017
Chlorbenzol	ja (31)	0,011	n. a.	HS-SPME-GC-MS	Blount et al. 2006
1,2‑Dichlorbenzol	ja (31)	0,100	n. a.	HS-SPME-GC-MS	Blount et al. 2006
	ja (10)	0,25	1,4	HS-NTD-GC-MS	Alonso et al. 2012
1,3-Dichlorbenzol	ja (31)	0,050	n. a.	HS-SPME-GC-MS	Blount et al. 2006
1,4-Dichlorbenzol	ja (31)	0,120	n. a.	HS-SPME-GC-MS	Blount et al. 2006
Ethylbenzol	ja (6)	0,01	n. a.	HS-SPME-GC-MS	Andreoli et al. 1999
	ja (20)	n. a.	≈ 10	HS-SPME-GC-MS	Liu et al. 2000
	ja (4)	0,022	n. a.	statische HS-GC-MS	Perbellini et al. 2002
	ja (31)	0,024	n. a.	HS-SPME-GC-MS	Blount et al. 2006
	ja (10)	0,2	n. a.	HS-NTD-GC-MS	Alonso et al. 2012
	ja (70)	0,042	0,127	HS-SPME-GC-MS	Mochalski et al. 2013
	ja (24)	n. a.	7,21–10,6	HS-SPME-GC-MS	Waters et al. 2017
2‑Ethyltoluol	ja (20)	n. a.	≈ 10	HS-SPME-GC-MS	Liu et al. 2000
	ja (24)	n. a.	7,21–10,6	HS-SPME-GC-MS	Waters et al. 2017
3‑Ethyltoluol	ja (20)	n. a.	≈ 10	HS-SPME-GC-MS	Liu et al. 2000
	ja (24)	n. a.	7,21–10,6	HS-SPME-GC-MS	Waters et al. 2017
Inden	ja (24)	n. a.	7,21–10,6	HS-SPME-GC-MS	Waters et al. 2017
Isopropylbenzol (Cumol)	ja (20)	n. a.	≈ 10	HS-SPME-GC-MS	Liu et al. 2000
	ja (24)	n. a.	7,21–10,6	HS-SPME-GC-MS	Waters et al. 2017
4-Isopropyltoluol (*p*‑Cymol)	ja (70)	0,013	0,040	HS-SPME-GC-MS	Mochalski et al. 2013
*α*‑Methylstyrol	ja (70)	0,012	0,036	HS-SPME-GC-MS	Mochalski et al. 2013
Naphthalin	ja (24)	n. a.	7,21–10,6	HS-SPME-GC-MS	Waters et al. 2017
*n*‑Propylbenzol	ja (20)	n. a.	≈ 10	HS-SPME-GC-MS	Liu et al. 2000
	ja (24)	n. a.	7,21–10,6	HS-SPME-GC-MS	Waters et al. 2017
Styrol	ja (31)	0,050	n. a.	HS-SPME-GC-MS	Blount et al. 2006
	ja (10)	0,1	1,4	HS-NTD-GC-MS	Alonso et al. 2012
	ja (70)	0,010	0,031	HS-SPME-GC-MS	Mochalski et al. 2013
	ja (24)	n. a.	7,21–10,6	HS-SPME-GC-MS	Waters et al. 2017
Toluol	ja (6)	0,005	n. a.	HS-SPME-GC-MS	Andreoli et al. 1999
	ja (20)	n. a.	≈ 10	HS-SPME-GC-MS	Liu et al. 2000
	ja (4)	0,043	n. a.	statische HS-GC-MS	Perbellini et al. 2002
	ja (31)	0,025	n. a.	HS-SPME-GC-MS	Blount et al. 2006
	ja (10)	0,2	1,4	HS-NTD-GC-MS	Alonso et al. 2012
	ja (70)	0,003	0,008	HS-SPME-GC-MS	Mochalski et al. 2013
	ja (18)	1000	n. a.	statische HS-GC-FID-MS	Tiscione et al. 2013
	ja (24)	n. a.	7,21–10,6	HS-SPME-GC-MS	Waters et al. 2017
1,2,3‑Trimethylbenzol (Hemimellitol)	ja (20)	n. a.	≈ 10	HS-SPME-GC-MS	Liu et al. 2000
	ja (24)	n. a.	7,21–10,6	HS-SPME-GC-MS	Waters et al. 2017
1,2,4‑Trimethylbenzol (Pseudocumol)	ja (20)	n. a.	≈ 10	HS-SPME-GC-MS	Liu et al. 2000
	ja (24)	n. a.	7,21–10,6	HS-SPME-GC-MS	Waters et al. 2017
1,3,5‑Trimethylbenzol (Mesitylen)	ja (20)	n. a.	≈ 10	HS-SPME-GC-MS	Liu et al. 2000
	ja (24)	n. a.	7,21–10,6	HS-SPME-GC-MS	Waters et al. 2017
*m*‑Xylol	ja (4)	0,052	n. a.	statische HS-GC-MS	Perbellini et al. 2002
(*m *+ *p*)‑Xylol	ja (6)	0,01	n. a.	HS-SPME-GC-MS	Andreoli et al. 1999
	ja (20)	n. a.	≈ 10	HS-SPME-GC-MS	Liu et al. 2000
	ja (31)	0,034	n. a.	HS-SPME-GC-MS	Blount et al. 2006
	ja (10)	0,3	1,3	HS-NTD-GC-MS	Alonso et al. 2012
	ja (70)	0,007	0,022	HS-SPME-GC-MS	Mochalski et al. 2013
	ja (24)	n. a.	7,21–10,6	HS-SPME-GC-MS	Waters et al. 2017
*o*‑Xylol	ja (6)	0,01	n. a.	HS-SPME-GC-MS	Andreoli et al. 1999
	ja (20)	n. a.	≈ 10	HS-SPME-GC-MS	Liu et al. 2000
	ja (31)	0,024	n. a.	HS-SPME-GC-MS	Blount et al. 2006
	ja (10)	0,2	1,3	HS-NTD-GC-MS	Alonso et al. 2012
	ja (70)	0,009	0,026	HS-SPME-GC-MS	Mochalski et al. 2013
	ja (24)	n. a.	7,21–10,6	HS-SPME-GC-MS	Waters et al. 2017
**Aliphatische Kohlenwasserstoffe**					
1,3‑Butadien	ja (3)	0,0005	n. a.	statische HS-GC-MS	Perbellini et al. 2003
	ja (70)	0,004	0,011	HS-SPME-GC-MS	Mochalski et al. 2013
*n*‑Butan	ja (70)	0,008	0,023	HS-SPME-GC-MS	Mochalski et al. 2013
*n*‑Decan	ja (20)	n. a.	≈ 10	HS-SPME-GC-MS	Liu et al. 2000
	ja (70)	0,043	0,128	HS-SPME-GC-MS	Mochalski et al. 2013
	ja (24)	n. a.	7,21–10,6	HS-SPME-GC-MS	Waters et al. 2017
2,3‑Dimethylbutan	ja (70)	0,005	0,016	HS-SPME-GC-MS	Mochalski et al. 2013
*n*‑Dodecan	ja (20)	n. a.	≈ 10	HS-SPME-GC-MS	Liu et al. 2000
	ja (24)	n. a.	7,21–10,6	HS-SPME-GC-MS	Waters et al. 2017
*n*‑Heptan	ja (20)	n. a.	≈ 10	HS-SPME-GC-MS	Liu et al. 2000
	ja (24)	n. a.	7,21–10,6	HS-SPME-GC-MS	Waters et al. 2017
*cis*,*trans*‑2,4‑Hexadien	ja (70)	0,002	0,005	HS-SPME-GC-MS	Mochalski et al. 2013
*n*‑Hexan	ja (20)	n. a.	≈ 10	HS-SPME-GC-MS	Liu et al. 2000
	ja (70)	0,002	0,005	HS-SPME-GC-MS	Mochalski et al. 2013
1‑Hexen	ja (70)	0,002	0,005	HS-SPME-GC-MS	Mochalski et al. 2013
Isopren	ja (70)	0,003	0,008	HS-SPME-GC-MS	Mochalski et al. 2013
2-Methylbutan (Isopentan)	ja (70)	0,005	0,015	HS-SPME-GC-MS	Mochalski et al. 2013
2-Methyl-1-buten	ja (70)	0,004	0,011	HS-SPME-GC-MS	Mochalski et al. 2013
2-Methylhexan	ja (70)	0,002	0,006	HS-SPME-GC-MS	Mochalski et al. 2013
4-Methyloctan	ja (70)	0,019	0,058	HS-SPME-GC-MS	Mochalski et al. 2013
2-Methylpentan	ja (70)	0,007	0,021	HS-SPME-GC-MS	Mochalski et al. 2013
4-Methyl-1-penten	ja (70)	0,003	0,008	HS-SPME-GC-MS	Mochalski et al. 2013
2-Methylpropan (Isobutan)	ja (70)	0,013	0,040	HS-SPME-GC-MS	Mochalski et al. 2013
2-Methyl-1-propen (Isobuten)	ja (70)	0,006	0,019	HS-SPME-GC-MS	Mochalski et al. 2013
*n*‑Nonan	ja (20)	n. a.	≈ 10	HS-SPME-GC-MS	Liu et al. 2000
	ja (24)	n. a.	7,21–10,6	HS-SPME-GC-MS	Waters et al. 2017
*n*‑Octan	ja (20)	n. a.	≈ 10	HS-SPME-GC-MS	Liu et al. 2000
	ja (70)	0,005	0,014	HS-SPME-GC-MS	Mochalski et al. 2013
	ja (24)	n. a.	7,21–10,6	HS-SPME-GC-MS	Waters et al. 2017
*cis*‑2-Penten	ja (70)	0,003	0,008	HS-SPME-GC-MS	Mochalski et al. 2013
*trans*‑2-Penten	ja (70)	0,003	0,008	HS-SPME-GC-MS	Mochalski et al. 2013
*cis*‑1,3‑Pentadien	ja (70)	0,001	0,004	HS-SPME-GC-MS	Mochalski et al. 2013
*trans*‑1,3‑Pentadien	ja (70)	0,002	0,006	HS-SPME-GC-MS	Mochalski et al. 2013
*n*-Pentan	ja (70)	0,007	0,022	HS-SPME-GC-MS	Mochalski et al. 2013
Propen (Propylen)	ja (70)	0,156	0,467	HS-SPME-GC-MS	Mochalski et al. 2013
*n*‑Tridecan	ja (20)	n. a.	≈ 10	HS-SPME-GC-MS	Liu et al. 2000
	ja (24)	n. a.	7,21–10,6	HS-SPME-GC-MS	Waters et al. 2017
*n*‑Undecan	ja (20)	n. a.	≈ 10	HS-SPME-GC-MS	Liu et al. 2000
	ja (70)	0,109	0,328	HS-SPME-GC-MS	Mochalski et al. 2013
	ja (24)	n. a.	7,21–10,6	HS-SPME-GC-MS	Waters et al. 2017
**Halogenierte Kohlenwasserstoffe**					
Bromchloriodmethan	ja (2)	0,002	n. a.	HS-SPME-GC-HRMS	Silva et al. 2006
Bromdichlormethan	ja (5)	0,0003	n. a.	HS-SPME-GC-HRMS	Bonin et al. 2005
	ja (5)	0,0004	n. a.	PT-HS-GC-HRMS	Bonin et al. 2005
	ja (31)	0,030	n. a.	HS-SPME-GC-MS	Blount et al. 2006
Chlordifluormethan (Freon-22)	ja (18)	5000	n. a.	statische HS-GC-FID-MS	Tiscione et al. 2013
Chlorethan	ja (18)	1900	n. a.	statische HS-GC-FID-MS	Tiscione et al. 2013
Dibromchlormethan	ja (5)	0,0004	n. a.	HS-SPME-GC-HRMS	Bonin et al. 2005
	ja (5)	0,0001	n. a.	PT-HS-GC-HRMS	Bonin et al. 2005
	ja (31)	0,005	n. a.	HS-SPME-GC-MS	Blount et al. 2006
Dibrommethan	ja (31)	0,030	n. a.	HS-SPME-GC-MS	Blount et al. 2006
Dichlordifluormethan (Freon-12)	ja (18)	5000	n. a.	statische HS-GC-FID-MS	Tiscione et al. 2013
1,1‑Dichlorethan	ja (31)	0,010	n. a.	HS-SPME-GC-MS	Blount et al. 2006
1,2‑Dichlorethan	ja (31)	0,009	n. a.	HS-SPME-GC-MS	Blount et al. 2006
1,1‑Dichlorethen	ja (31)	0,009	n. a.	HS-SPME-GC-MS	Blount et al. 2006
*cis*‑1,2‑Dichlorethen	ja (31)	0,010	n. a.	HS-SPME-GC-MS	Blount et al. 2006
*trans*‑1,2‑Dichlorethen	ja (31)	0,009	n. a.	HS-SPME-GC-MS	Blount et al. 2006
Dichlorfluormethan (Freon-21)	ja (18)	5000	n. a.	statische HS-GC-FID-MS	Tiscione et al. 2013
Dichloriodmethan	ja (2)	0,002	n. a.	HS-SPME-GC-HRMS	Silva et al. 2006
Dichlormethan (Methylenchlorid)	ja (31)	0,070	n. a.	HS-SPME-GC-MS	Blount et al. 2006
1,2‑Dichlorpropan	ja (31)	0,008	n. a.	HS-SPME-GC-MS	Blount et al. 2006
	ja (10)	0,2	1,8	HS-NTD-GC-MS	Alonso et al. 2012
1,2‑Dichlortetrafluorethan (Freon-114)	ja (18)	5000	n. a.	statische HS-GC-FID-MS	Tiscione et al. 2013
1,1‑Difluorethan	ja (18)	< 2600	n. a.	statische HS-GC-FID-MS	Tiscione et al. 2013
Fluortrichlormethan (Freon-11)	ja (18)	5000	n. a.	statische HS-GC-FID-MS	Tiscione et al. 2013
Hexachlorethan	ja (31)	0,011	n. a.	HS-SPME-GC-MS	Blount et al. 2006
1,1,2,2‑Tetrachlorethan	ja (31)	0,010	n. a.	HS-SPME-GC-MS	Blount et al. 2006
Tetrachlorethen	ja (31)	0,048	n. a.	HS-SPME-GC-MS	Blount et al. 2006
Tetrachlormethan (Tetrachlorkohlenstoff)	ja (31)	0,005	n. a.	HS-SPME-GC-MS	Blount et al. 2006
1,1,1,2‑Tetrafluorethan	ja (18)	20 000	n. a.	statische HS-GC-FID-MS	Tiscione et al. 2013
Tribrommethan (Bromoform)	ja (5)	0,0006	n. a.	HS-SPME-GC-HRMS	Bonin et al. 2005
	ja (5)	0,0002	n. a.	PT-HS-GC-HRMS	Bonin et al. 2005
	ja (31)	0,020	n. a.	HS-SPME-GC-MS	Blount et al. 2006
1,1,1‑Trichlorethan	–	n. a.	0,8	PT-HS-GC-MS	Johns et al. 2005
	ja (31)	0,048	n.a	HS-SPME-GC-MS	Blount et al. 2006
1,1,2‑Trichlorethan	ja (31)	0,010	n. a.	HS-SPME-GC-MS	Blount et al. 2006
Trichlorethen	ja (31)	0,012	n. a.	HS-SPME-GC-MS	Blount et al. 2006
Trichlormethan (Chloroform)	ja (5)	0,0024	n. a.	HS-SPME-GC-HRMS	Bonin et al. 2005
	ja (5)	0,0032	n. a.	PT-HS-GC-HRMS	Bonin et al. 2005
	ja (31)	0,020	n. a.	HS-SPME-GC-MS	Blount et al. 2006
1,1,1‑Trifluorethan (Freon-143a)	ja (18)	3400	n. a.	statische HS-GC-FID-MS	Tiscione et al. 2013
**Alkohole, Aldehyde, Ketone und Ether**					
Acetaldehyd	ja (7)	15 333	46 000	HS-GC-FID	Kovatsi et al. 2011
	ja (18)	18 750	n. a.	statische HS-GC-FID-MS	Tiscione et al. 2013
	ja (5)	100	500	statische HS-GC-MS	Cordell et al. 2013
	ja (20)	50,6 (Serum)	n. a.	HS-SPME-GC-HRMS	Silva et al. 2018
Aceton	ja (7)	7333	22 000	HS-GC-FID	Kovatsi et al. 2011
	ja (18)	25 000	n. a.	statische HS-GC-FID-MS	Tiscione et al. 2013
	ja (5)	100	500	statische HS-GC-MS	Cordell et al. 2013
*tert*‑Amylmethylether	ja (4)	0,0006	n. a.	HS-SPME-GC-HRMS	Silva et al. 2008
Benzaldehyd	ja (70)	0,265	0,796	HS-SPME-GC-MS	Mochalski et al. 2013
	ja (20)	0,461 (Serum)	n. a.	HS-SPME-GC-HRMS	Silva et al. 2018
Butanal	ja (20)	0,313 (Serum)	n. a.	HS-SPME-GC-HRMS	Silva et al. 2018
2,3‑Butandion (Dimethyldiketon)	ja (70)	0,344	1,03	HS-SPME-GC-MS	Mochalski et al. 2013
1‑Butanol	ja (18)	25 000	n. a.	statische HS-GC-FID-MS	Tiscione et al. 2013
*tert*‑Butanol	ja (2)	0,05 (Serum)	0,15 (Serum)	HS-SPME-GC-MS	Zhang et al. 2015
2-Butanon (Methylethylketon)	ja (70)	0,029	0,087	HS-SPME-GC-MS	Mochalski et al. 2013
	ja (18)	5000	n. a.	statische HS-GC-FID-MS	Tiscione et al. 2013
Crotonaldehyd	ja (20)	0,147 (Serum)	n. a.	HS-SPME-GC-HRMS	Silva et al. 2018
Decanal	ja (20)	3,90 (Serum)	n. a.	HS-SPME-GC-HRMS	Silva et al. 2018
Diisopropylether	ja (4)	0,0006	n. a.	HS-SPME-GC-HRMS	Silva et al. 2008
Ethanol	ja (7)	15 667	47 000	HS-GC-FID	Kovatsi et al. 2011
	ja (5)	100	500	statische HS-GC-MS	Cordell et al. 2013
Ethylacetat	ja (70)	0,009	0,026	HS-SPME-GC-MS	Mochalski et al. 2013
Ethyl‑*tert*‑butylether	ja (4)	0,0006	n. a.	HS-SPME-GC-HRMS	Silva et al. 2008
Ethylvinylether	ja (70)	0,003	0,009	HS-SPME-GC-MS	Mochalski et al. 2013
Fufural (2-Furaldehyd)	ja (20)	1,24 (Serum)	n. a.	HS-SPME-GC-HRMS	Silva et al. 2018
Heptanal	ja (20)	0,312 (Serum)	n. a.	HS-SPME-GC-HRMS	Silva et al. 2018
	ja (2)	0,01 (Plasma)	n. a.	HS-SPME-GC-FID	Ghaedrahmati et al. 2021
2‑Heptanon	ja (70)	0,023	0,069	HS-SPME-GC-MS	Mochalski et al. 2013
4‑Heptanon	ja (70)	0,006	0,017	HS-SPME-GC-MS	Mochalski et al. 2013
Hexanal	ja (20)	0,693 (Serum)	n. a.	HS-SPME-GC-HRMS	Silva et al. 2018
	ja (2)	0,001 (Plasma)	n. a.	HS-SPME-GC-FID	Ghaedrahmati et al. 2021
2‑Hexanon	ja (70)	0,015	0,045	HS-SPME-GC-MS	Mochalski et al. 2013
3‑Hexanon	ja (70)	0,015	0,045	HS-SPME-GC-MS	Mochalski et al. 2013
*trans*‑2‑Hexenal	ja (20)	0,290 (Serum)	n. a.	HS-SPME-GC-HRMS	Silva et al. 2018
Isobutanol (2-Methyl-1-propanol)	ja (18)	50 000	n. a.	statische HS-GC-FID-MS	Tiscione et al. 2013
Isopentanal (Isovaleraldehyd)	ja (20)	0,119 (Serum)	n. a.	HS-SPME-GC-HRMS	Silva et al. 2018
Methanol	ja (7)	15 000	45 000	HS-GC-FID	Kovatsi et al. 2011
	ja (5)	200	1000	statische HS-GC-MS	Cordell et al. 2013
	ja (18)	250 000	n. a.	statische HS-GC-FID-MS	Tiscione et al. 2013
Methylacetat	ja (70)	0,074	0,222	HS-SPME-GC-MS	Mochalski et al. 2013
2-Methylbenzaldehyd (*o*‑Tolualdehyd)	ja (20)	0,142 (Serum)	n. a.	HS-SPME-GC-HRMS	Silva et al. 2018
3-Methyl-1-butanol (Isopentanol)	ja (18)	25 000	n. a.	statische HS-GC-FID-MS	Tiscione et al. 2013
Methyl-*tert*‑butylether (2-Methoxy-2‑methylpropan)	ja (5)	0,0015	n. a.	HS-SPME-GC-HRMS	Bonin et al. 2005
	ja (5)	0,0045	n. a.	PT-HS-GC-HRMS	Bonin et al. 2005
	ja (31)	0,100	n. a.	HS-SPME-GC-MS	Blount et al. 2006
	ja (4)	0,0006	n. a.	HS-SPME-GC-HRMS	Silva et al. 2008
	ja (2)	0,03 (Serum)	0,09 (Serum)	HS-SPME-GC-MS	Zhang et al. 2015
2-Methyl-1-propanal (Isobutanal)	ja (20)	0,109 (Serum)	n. a.	HS-SPME-GC-HRMS	Silva et al. 2018
2-Methyl-2-propenal	ja (70)	0,063	0,189	HS-SPME-GC-MS	Mochalski et al. 2013
Methylpropionat	ja (70)	0,012	0,034	HS-SPME-GC-MS	Mochalski et al. 2013
Methylvinylketon (3-Buten-2-on)	ja (70)	2,80	8,41	HS-SPME-GC-MS	Mochalski et al. 2013
Nonanal	ja (20)	2,63 (Serum)	n. a.	HS-SPME-GC-HRMS	Silva et al. 2018
*trans*‑2‑Nonenal	ja (20)	2,68 (Serum)	n. a.	HS-SPME-GC-HRMS	Silva et al. 2018
Octanal	ja (20)	0,660 (Serum)	n. a.	HS-SPME-GC-HRMS	Silva et al. 2018
*trans*‑2‑Octenal	ja (20)	1,12 (Serum)	n. a.	HS-SPME-GC-HRMS	Silva et al. 2018
Pentanal	ja (20)	0,316 (Serum)	n. a.	HS-SPME-GC-HRMS	Silva et al. 2018
2‑Pentanon	ja (70)	0,022	0,065	HS-SPME-GC-MS	Mochalski et al. 2013
*trans*‑3‑Penten‑2‑on	ja (70)	0,210	0,631	HS-SPME-GC-MS	Mochalski et al. 2013
Propanal	ja (70)	0,076	0,227	HS-SPME-GC-MS	Mochalski et al. 2013
	ja (20)	1,16 (Serum)	n. a.	HS-SPME-GC-HRMS	Silva et al. 2018
2-Propenal (Acrolein)	ja (70)	15,1	45,4	HS-SPME-GC-MS	Mochalski et al. 2013
	ja (20)	2,16 (Serum)	n. a.	HS-SPME-GC-HRMS	Silva et al. 2018
1‑Propanol	ja (7)	8333	25 000	HS-GC-FID	Kovatsi et al. 2011
2‑Propanol (Isopropanol)	ja (18)	100 000	n. a.	statische HS-GC-FID-MS	Tiscione et al. 2013
**Inhalationsnarkotika**					
Brommethan als Metabolit des Halothans	ja (2)	3995–6392 (Plasma)	n. a.	statische HS-GC-FID	Maiorino et al. 1980
Desfluran	ja (7)	11 333	34 000	HS-GC-FID	Kovatsi et al. 2011
	–	n. a.	n. a.	HS-GC-MS/MS	Tamura et al. 2020
Halothan (2-Brom-2‑chlor-1,1,1‑trifluorethan) mit Enfluran als ISTD	–	≈ 4	≈ 50	HS-SPME-GC-MS	Musshoff et al. 2000
Sevofluran	ja (7)	17 333	52 000	HS-GC-FID	Kovatsi et al. 2011
	–	n. a.	n. a.	HS-GC-FID	Lin et al. 2015
	–	n. a.	n. a.	HS-GC-MS/MS	Tamura et al. 2020
Trifluoressigsäure als Metabolit des Halothans, Isoflurans und Fluroxens	ja (2)	285 (Plasma)	n. a.	statische HS-GC-FID	Maiorino et al. 1980
**Sonstige**					
Acetonitril	ja (70)	0,608	1,82	HS-SPME-GC-MS	Mochalski et al. 2013
Allylmethylsulfid	ja (70)	0,003	0,008	HS-SPME-GC-MS	Mochalski et al. 2013
3‑Caren	ja (70)	0,123	0,368	HS-SPME-GC-MS	Mochalski et al. 2013
1,8‑Cineol (Eucalyptol)	ja (70)	0,123	0,370	HS-SPME-GC-MS	Mochalski et al. 2013
Cyanwasserstoff	–	13,8	n. a.	statische HS-GC-NPD	Calafat und Stanfill 2002
2,5‑Dimethylfuran	ja (3)	0,005	n. a.	statische HS-GC-MS	Perbellini et al. 2003
	ja (31)	0,012	n. a.	HS-SPME-GC-MS	Blount et al. 2006
	ja (10)	0,1	1,4	HS-NTD-GC-MS	Alonso et al. 2012
	ja (70)	0,002	0,007	HS-SPME-GC-MS	Mochalski et al. 2013
	ja (20)	0,038 (Serum)	n. a.	HS-SPME-GC-HRMS	Silva et al. 2018
Dimethylselenid	ja (70)	0,003	0,010	HS-SPME-GC-MS	Mochalski et al. 2013
Dimethylsulfid	ja (70)	0,006	0,019	HS-SPME-GC-MS	Mochalski et al. 2013
Essigsäure	ja (5)	100	500	statische HS-GC-MS	Cordell et al. 2013
Ethylmethylsulfid	ja (70)	0,005	0,014	HS-SPME-GC-MS	Mochalski et al. 2013
Furan	ja (10)	0,2	1,0	HS-NTD-GC-MS	Alonso et al. 2012
	ja (70)	0,001	0,003	HS-SPME-GC-MS	Mochalski et al. 2013
Limonen (1-Methyl-4-(1-methylvinyl)cyclohexen)	ja (70)	0,011	0,033	HS-SPME-GC-MS	Mochalski et al. 2013
Menthon	ja (70)	0,093	0,278	HS-SPME-GC-MS	Mochalski et al. 2013
2‑Methylfuran	ja (70)	0,001	0,003	HS-SPME-GC-MS	Mochalski et al. 2013
3‑Methylfuran	ja (70)	0,001	0,004	HS-SPME-GC-MS	Mochalski et al. 2013
Methylpropylsulfid	ja (70)	0,004	0,011	HS-SPME-GC-MS	Mochalski et al. 2013
1‑Methylpyrrol	ja (70)	0,008	0,024	HS-SPME-GC-MS	Mochalski et al. 2013
3‑Methylthiophen	ja (70)	0,002	0,006	HS-SPME-GC-MS	Mochalski et al. 2013
*α‑*Pinen	ja (70)	0,008	0,025	HS-SPME-GC-MS	Mochalski et al. 2013
*β*‑Pinen	ja (70)	0,005	0,016	HS-SPME-GC-MS	Mochalski et al. 2013
Pyrazin	ja (70)	0,360	1,08	HS-SPME-GC-MS	Mochalski et al. 2013
Pyrrol	ja (70)	0,001	0,003	HS-SPME-GC-MS	Mochalski et al. 2013
*γ*‑Terpinen	ja (70)	0,136	0,409	HS-SPME-GC-MS	Mochalski et al. 2013
Thiophen (Thiofuran)	ja (70)	0,001	0,003	HS-SPME-GC-MS	Mochalski et al. 2013

Abkürzungen siehe Abkürzungsverzeichnis

**Tab.7:** Beurteilungswerte, deren Parameter mit Headspace-Methoden erfasst werden können

Substanz (Synonym)	Analyt	Matrix	Probenahmezeitpunkt	Beurteilungswerte	Wert	Fachgremium, Land	Literatur
Aceton	Aceton	Urin	Expositionsende bzw. Schichtende	BAR	2,5 mg/l	MAK-Kommission, Deutschland	DFG 2025
				BAT	50 mg/l		
				BGW	50 mg/l	AGS, Deutschland	AGS 2013
				BAT-Suva	50 mg/l	Suva, Schweiz	Koller et al. 2018; SUVA 2025 a, b
			innerhalb 2 Stunden vor Schichtende	OEL-B	40 mg/l	JSOH, Japan	JSOH 2023
			Schichtende	BEI^®^	25 mg/l	BEI-Komitee, USA	ACGIH 2025
Benzol	Benzol	Blut	vor der Schicht am Ende der Arbeitswoche	BAL	1,6 μg/l	FIOH, Finnland	Kiilunen 1999
			Expositionsende	BLV	28 μg/l	SCOEL, EU‑Kommission	SCOEL 2006
		Urin	Expositionsende bzw. Schichtende	BAR	0,3 μg/l^a)^	MAK-Kommission, Deutschland	DFG 2025
				EKA	0,5–12,5 μg/l		
				Äquivalenzwert zur Toleranzkonzentration für krebserzeugende Gefahrstoffe	5 μg/l	AGS, Deutschland	AGS 2014
				Äquivalenzwert zur Akzeptanzkonzentration für krebserzeugende Gefahrstoffe	0,8 μg/l^a)^		
				BGV	0,3 μg/l	RAC, EU‑Kommission	RAC 2018
				BLV	0,7 μg/l		
1-Butanol	1-Butanol	Urin	Expositionsende bzw. Schichtende	BAT	10 mg/g Kreatinin	MAK-Kommission, Deutschland	DFG 2025
				BAT-Suva	10 mg/g Kreatinin	Suva, Schweiz	Koller et al. 2018; SUVA 2025 a, b
				BGV	10 mg/g Kreatinin^b)^	AGS, Deutschland	AGS 2013
			vor nachfolgender Schicht	BAT	2 mg/g Kreatinin	MAK-Kommission, Deutschland	DFG 2025
				BGV	2 mg/g Kreatinin^b)^	AGS, Deutschland	AGS 2013
			vor nachfolgender Schicht bzw. 16 h nach Expositionsende	BAT-Suva	2 mg/g Kreatinin	Suva, Schweiz	Koller et al. 2018; SUVA 2025 a, b
2-Butanon (Methylethylketon)	2-Butanon	Urin	Expositionsende bzw. Schichtende	BAT	2 mg/l	MAK-Kommission, Deutschland	DFG 2025
				BGV	2 mg/l	AGS, Deutschland	AGS 2013
			Expositionsende bzw. Schichtende, vor nachfolgender Schicht bzw. 16 h nach Expositionsende	BAT-Suva	2 mg/l	Suva, Schweiz	Koller et al. 2018; SUVA 2025 a, b
			am Schichtende	BEI^®^	2 mg/l	BEI-Komitee, USA	ACGIH 2025
				BLV	5,0 mg/l	SCOEL, EU‑Kommission	SCOEL 1999
				BMGV	70 μmol/l (5 mg/l)	HSE, Vereinigtes Königreich	HSE 2020, 2025
			am Schichtende oder einige Stunden nach hoher Exposition	OEL-B	5 mg/l	JSOH, Japan	JSOH 2023
			am Schichtende am Ende der Arbeitswoche	BAL	4,3 mg/l	FIOH, Finnland	Kiilunen 1999
Cyclohexanon	Cyclohexanol	Urin	am Schichtende	BMGV	2 mmol/mol Kreatinin	HSE, Vereinigtes Königreich	HSE 2020, 2025
				BEI^®^	8 mg/l	BEI-Komitee, USA	ACGIH 2025
Dichlormethan (Methylenchlorid)	Dichlormethan	Blut	unmittelbar nach Exposition	EKA	100–1000 μg/l	MAK-Kommission, Deutschland	DFG 2025
				BAT	500 μg/l		
				BGW	500 μg/l	AGS, Deutschland	AGS 2013
			Expositionsende bzw. Schichtende	BAT-Suva	500 μg/l	Suva, Schweiz	Koller et al. 2018; SUVA 2025 a, b
			am Schichtende	BLV	1000 μg/l	SCOEL, EU‑Kommission	SCOEL 2009 a
		Urin	am Schichtende	BLV	300 μg/l	SCOEL, EU‑Kommission	SCOEL 2009 a
				BEI^®^	300 μg/l	BEI-Komitee, USA	ACGIH 2025
				OEL-B	200 μg/l	JSOH, Japan	JSOH 2023
			Expositionsende bzw. Schichtende	VLB	200 μg/l	ANSES, Frankreich	ANSES 2017
				VBR	1,6 μg/l		
	CO-Hb	Blut	Expositionsende bzw. Schichtende	BAT-Suva	5 %	Suva, Schweiz	Koller et al. 2018; SUVA 2025 a, b
			am Schichtende	BLV	4 %	SCOEL, EU‑Kommission	SCOEL 2009 a
			unmittelbar nach Exposition oder am Schichtende	VLB	3,5 %^a)^	ANSES, Frankreich	ANSES 2017
				VBR	1,5 %^a)^		
	CO	Ausatemluft	am Schichtende	BMGV	30 ppm	HSE, Vereinigtes Königreich	HSE 2020, 2025
Ethylbenzol	Ethylbenzol	Urin	am Schichtende	OEL-B	15 μg/l	JSOH, Japan	JSOH 2023
Halothan (2-Brom-2‑chlor-1,1,1‑trifluorethan)	Trifluoressigsäure	Blut	Expositionsende bzw. Schichtende, bei Langzeitexpositionen am Schichtende nach mehreren vorangegangenen Schichten	BGW	2,5 mg/l	AGS, Deutschland	AGS 2013
				BAT	2,5 mg/l	MAK-Kommission, Deutschland	DFG 2025
				BAT-Suva	2,5 mg/l	Suva, Schweiz	Koller et al. 2018; SUVA 2025 a, b
*n*-Heptan	Heptan-2,5-dion	Urin	Expositionsende bzw. Schichtende	BAT	250 μg/l	MAK-Kommission, Deutschland	DFG 2025
*n*-Hexan	Hexan-2,5-dion	Urin	am Schichtende	BEI^®^	0,5 mg/g Krea^c)^	BEI-Komitee, USA	ACGIH 2025
			am Schichtende am Ende der Arbeitswoche	OEL-B	3 mg/g Krea^b)^	JSOH, Japan	JSOH 2023
					0,3 mg/g Krea^d)^		
	Hexan-1,2-dion	Urin	am Schichtende	BAL	0,57 mg/l	FIOH, Finnland	Kiilunen 1999
2-Hexanon	Hexan-2,5-dion, ohne Hydrolyse	Urin	am Schichtende	BEI^®^	0,5 mg/l	BEI-Komitee, USA	ACGIH 2025
Kohlenmonoxid	CO-Hb	Blut	Expositionsende bzw. Schichtende	BAT	5 %	MAK-Kommission, Deutschland	DFG 2025
				BAL	4 %	FIOH, Finnland	Kiilunen 1999
			am Schichtende	BEI^®^	3,5 %	BEI-Komitee, USA	ACGIH 2025
	CO	Ausatemluft	am Schichtende	BEI^®^	20 ppm	BEI-Komitee, USA	ACGIH 2025
Methanol	Methanol	Urin	Expositionsende bzw. Schichtende	BGW	15 mg/l	AGS, Deutschland	AGS 2013
				BAT	15 mg/l	MAK-Kommission, Deutschland	DFG 2025
			Expositionsende bzw. Schichtende, bei Langzeitexpositionen am Schichtende nach mehreren vorangegangenen Schichten	BAT-Suva	30 mg/l	Suva, Schweiz	Koller et al. 2018; SUVA 2025 a, b
			am Schichtende	OEL-B	20 mg/l	JSOH, Japan	JSOH 2023
				BEI^®^	15 mg/l	BEI-Komitee, USA	ACGIH 2025
Methyl-*tert*‑butylether (2-Methoxy-2‑methylpropan)	Methyl-*tert*‑butylether	Blut	Expositionsende bzw. Schichtende	BAT	nicht festgelegt	MAK-Kommission, Deutschland	DFG 2025
			–	VLB	nicht festgelegt	ANSES, Frankreich	ANSES 2022
				VBR	nicht festgelegt		
		Urin	Expositionsende bzw. Schichtende	BAT	nicht festgelegt	MAK-Kommission, Deutschland	DFG 2025
	*tert*-Butanol	Blut	–	BAT	nicht festgelegt	MAK-Kommission, Deutschland	DFG 2025
		Urin	–	BAT	nicht festgelegt	MAK-Kommission, Deutschland	DFG 2025
Methylformiat	Methanol	Urin	Expositionsende bzw. Schichtende	BAT	nicht festgelegt	MAK-Kommission, Deutschland	DFG 2025
4-Methylpentan-2‑on (Methylisobutylketon)	4-Methylpentan-2‑on	Urin	Expositionsende bzw. Schichtende	BAT	0,7 mg/l	MAK-Kommission, Deutschland	DFG 2025
				BAT-Suva	0,7 mg/l	Suva, Schweiz	Koller et al. 2018; SUVA 2025 a, b
				BGW	0,7 mg/l	AGS, Deutschland	AGS 2013
			am Schichtende	BEI^®^	1 mg/l	BEI-Komitee, USA	ACGIH 2025
				OEL-B	1,7 mg/l	JSOH, Japan	JSOH 2023
				BMGV	20 μmol/l (2 mg/l)	HSE, Vereinigtes Königreich	HSE 2020, 2025
2‑Propanol (Isopropanol)	Aceton	Blut	Expositionsende bzw. Schichtende	BAT	25 mg/l	MAK-Kommission, Deutschland	DFG 2025
				BGW	25 mg/l	AGS, Deutschland	AGS 2013
				BAT-Suva	25 mg/l	Suva, Schweiz	Koller et al. 2018; SUVA 2025 a, b
		Urin	Expositionsende bzw. Schichtende	BAT	25 mg/l	MAK-Kommission, Deutschland	DFG 2025
				BGW	25 mg/l	AGS, Deutschland	AGS 2013
				BAT-Suva	25 mg/l	Suva, Schweiz	Koller et al. 2018; SUVA 2025 a, b
			am Schichtende am Ende der Arbeitswoche	BEI^®^	40 mg/l	BEI-Komitee, USA	ACGIH 2025
Styrol	Styrol	Urin	am Schichtende	BEI^®^	40 μg/l	BEI-Komitee, USA	ACGIH 2025
				VLB	40 μg/l	ANSES, Frankreich	ANSES 2014
			am Schichtende am Ende der Arbeitswoche	OEL-B	20 μg/l	JSOH, Japan	JSOH 2023
Tetrachlorethen	Tetrachlorethen	Ausatemluft	vor letzter Schicht	BEI^®^	3 ppm	BEI-Komitee, USA	ACGIH 2025
			vor letzter Schicht der Arbeitswoche	BLV	3 ppm	SCOEL, EU‑Kommission	SCOEL 2009 b
		Blut	16 h nach Expositionsende	BAT	200 μg/l	MAK-Kommission, Deutschland	DFG 2025
				BGW	200 μg/l	AGS, Deutschland	AGS 2013
				EKA	60–1000 μg/l	MAK-Kommission, Deutschland	DFG 2025
			vor letzter Schicht der Arbeitswoche	BLV	400 μg/l	SCOEL, EU‑Kommission	SCOEL 2009 b
			vor nachfolgender Schicht	BAT-Suva	400 μg/l	Suva, Schweiz	Koller et al. 2018; SUVA 2025 a, b
			vor der Schicht	BEI^®^	500 μg/l	BEI-Komitee, USA	ACGIH 2025
			vor der Schicht am Ende der Arbeitswoche	BAL	1,0 mg/l	FIOH, Finnland	Kiilunen 1999
			am Morgen nach dem Arbeitstag	HTP	1,2 μmol/l (199 μg/l)	Ministerium für soziale Angelegenheiten und Gesundheit, Finnland	STM 2025
			vor letzter Schicht der Arbeitswoche	VLB	500 μg/l	ANSES, Frankreich	ANSES 2018
				VBR	0,12 μg/l		
		Urin	am Schichtende am Ende der Arbeitswoche	VLB	50 μg/l	ANSES, Frankreich	ANSES 2018
				VBR	0,40 μg/l		
	Trichloressigsäure	Urin	am Schichtende am Ende der Arbeitswoche	VGÜ-Grenzwert	40 mg/l	Bundesministerium für Arbeit und Wirtschaft, Österreich	BAW 2024
Tetrachlormethan (Tetrachlorkohlenstoff)	Tetrachlormethan	Blut	am Schichtende, bei Langzeitexposition nach mehreren vorangegangenen Schichten	BGW	3,5 μg/l	AGS, Deutschland	AGS 2013
				BAT	3,5 μg/l	MAK-Kommission, Deutschland	DFG 2025
Tetrahydrofuran	Tetrahydrofuran	Urin	Expositionsende bzw. Schichtende	BAT	2 mg/l	MAK-Kommission, Deutschland	DFG 2025
				BGW	2 mg/l	AGS, Deutschland	AGS 2013
				BAT-Suva	2 mg/l	Suva, Schweiz	Koller et al. 2018; SUVA 2025 a, b
			am Schichtende	BEI^®^	2 mg/l	BEI-Komitee, USA	ACGIH 2025
				OEL-B	2 mg/l	JSOH, Japan	JSOH 2023
Toluol	Toluol	Blut	unmittelbar nach Exposition	BAT	600 μg/l	MAK-Kommission, Deutschland	DFG 2025
				BGW	600 μg/l	AGS, Deutschland	AGS 2013
			Expositionsende bzw. Schichtende	BAT-Suva	600 μg/l	Suva, Schweiz	Koller et al. 2018; SUVA 2025 a, b
			am Ende des Arbeitstages	VGÜ-Grenzwert	250 μg/l	Bundesministerium für Arbeit und Wirtschaft, Österreich	BAW 2024
			vor letzter Schicht der Arbeitswoche	BEI^®^	20 μg/l	BEI-Komitee, USA	ACGIH 2025
				VLB	20 μg/l	ANSES, Frankreich	ANSES 2011
				VBR	1 μg/l		
			vor der Schicht am Ende der Arbeitswoche	BAL	92 μg/l	FIOH, Finnland	Kiilunen 1999
			am Morgen nach dem Arbeitstag	HTP	500 nmol/l (46 μg/l)	Ministerium für soziale Angelegenheiten und Gesundheit, Finnland	STM 2025
			innerhalb von 2 Stunden vor Schichtende am Ende der Arbeitswoche	OEL-B	600 μg/l	JSOH, Japan	JSOH 2023
		Urin	Expositionsende bzw. Schichtende	BAT	75 μg/l	MAK-Kommission, Deutschland	DFG 2025
				BGW	75 μg/l	AGS, Deutschland	AGS 2013
				BAT-Suva	75 μg/l	Suva, Schweiz	Koller et al. 2018; SUVA 2025 a, b
			am Schichtende	BEI^®^	30 μg/l	BEI-Komitee, USA	ACGIH 2025
				VLB	30 μg/l	ANSES, Frankreich	ANSES 2011
				VBR	0,4 μg/l		
			innerhalb von 2 Stunden vor Schichtende am Ende der Arbeitswoche	OEL-B	60 μg/l	JSOH, Japan	JSOH 2023
1,1,1-Trichlorethan	1,1,1-Trichlorethan	Ausatemluft	vor letzter Schicht der Arbeitswoche	BEI^®^	20 ppm	BEI-Komitee, USA	ACGIH 2025
		Blut	vor nachfolgender Schicht, nach mehreren vorangegangenen Schichten	BAT	275 μg/l	MAK-Kommission, Deutschland	DFG 2025
			nach mehreren vorangegangenen Schichten vor nachfolgender Schicht	BGW	275 μg/l	AGS, Deutschland	AGS 2013
			bei Langzeitexpositionen am Schichtende nach mehreren vorangegangenen Schichten	BAT-Suva	275 μg/l	Suva, Schweiz	Koller et al. 2018; SUVA 2025 a, b
			vor letzter Schicht der Arbeitswoche	BAL	266 μg/l	FIOH, Finnland	Kiilunen 1999
		Urin	am Schichtende	BEI^®^	700 μg/l	BEI-Komitee, USA	ACGIH 2025
Trichlorethen	Trichlorethen	Ausatemluft	–	BEI^®e)^	–	BEI-Komitee, USA	ACGIH 2025
		Blut	–	BEI^®e)^	–	BEI-Komitee, USA	ACGIH 2025
		Urin	am Schichtende	VLB	10 μg/l	ANSES, Frankreich	ANSES 2020
				VBR	1,5 μg/l		
	Trichloressigsäure	Urin	am Schichtende, bei Langzeitexposition nach mehreren vorangegangenen Schichten	BAR	0,07 mg/l	MAK-Kommission, Deutschland	DFG 2025
				EKA	1,2–50 mg/l		
			Expositionsende bzw. Schichtende, bei Langzeitexpositionen am Schichtende nach mehreren vorangegangenen Schichten	BAT-Suva	40 mg/l	Suva, Schweiz	Koller et al. 2018; SUVA 2025 a, b
				Äquivalenzwert zur Toleranzkonzentration für krebserzeugende Gefahrstoffe	22 mg/l	AGS, Deutschland	AGS 2014
				Äquivalenzwert zur Akzeptanzkonzentration für krebserzeugende Gefahrstoffe	12 mg/l		
Trichlorethen	Trichloressigsäure	Urin	am Schichtende der letzten Schicht der Arbeitswoche	BLV	20 mg/l	SCOEL, EU‑Kommission	SCOEL 2009 c
				BEI^®^	15 mg/l	BEI-Komitee, USA	ACGIH 2025
				VLB	15 mg/g Krea (21 mg/l)	ANSES, Frankreich	ANSES 2020
				VBR	9 μg/g Krea (8 μg/l)		
			am Schichtende am Ende der Arbeitswoche	OEL-B	10 mg/l	JSOH, Japan	JSOH 2023
				BAL	59 mg/l	FIOH, Finnland	Kiilunen 1999
			Expositionsende bzw. Schichtende	HTP	120 μmol/l (16 mg/l)	Ministerium für soziale Angelegenheiten und Gesundheit, Finnland	STM 2025
	Trichlorethanol	Urin	am Schichtende der letzten Schicht der Arbeitswoche	BEI^®^	0,5 mg/l	BEI-Komitee, USA	ACGIH 2025
1,1,2-Trichlor-1,2,2-trifluorethan (Freon-113)	1,1,2-Trichlor-1,2,2‑trifluorethan	Blut	am Schichtende der letzten Schicht der Arbeitswoche	BAL	9,3 μg/l	FIOH, Finnland	Kiilunen 1999
Xylol, alle Isomere	Xylol, alle Isomere	Blut	Expositionsende bzw. Schichtende	BAT^f)^	1,5 mg/l	MAK-Kommission, Deutschland	DFG 2025
			am Ende des Arbeitstages	VGÜ-Grenzwert	1,0 mg/l	Bundesministerium für Arbeit und Wirtschaft, Österreich	BAW 2024

^a)^ für Nichtraucher abgeleitet

^b)^ mit Hydrolyse

^c)^ ohne Hydrolyse, nicht mit Headspace bestimmt

^d)^ ohne Hydrolyse

^e)^ semiquantitativ

^f)^ bis 2014

Abkürzungen siehe Abkürzungsverzeichnis

**Tab.8:** Hintergrundbelastungen der beruflich nicht belasteten Allgemeinbevölkerung

Analyt (Synonym)	Matrix	Studienkollektiv	Anzahl der Personen	Referenzwert [μg/l]			Literatur
				Median	95. Perzentil	Bereich	
Acetonitril	Blut	gesunde Erwachsene	28	30,6^a)^	n. a.	< 0,61–95,8	Mochalski et al. 2013
Allylmethylsulfid	Blut	gesunde Erwachsene	28	0,24^a)^	n. a.	< 0,003–1,91	Mochalski et al. 2013
*tert*‑Amylmethylether	Blut	gesunde Erwachsene	3	< 0,0006	n. a.	< 0,0006	Silva et al. 2008
Benzaldehyd	Blut	gesunde Erwachsene	28	< 0,27^a)^	n. a.	< 0,27	Mochalski et al. 2013
Benzol	Blut	Nichtraucher	15	0,087	n. a.	0,046–0,472	Perbellini et al. 2002
		Raucher	10	0,246	n. a.	0,051–1,187	
		gesunde Erwachsene	28	0,020^a)^	n. a.	< 0,001–0,077	Mochalski et al. 2013
		Nichtraucher	46	0,051	n. a.	0,034–0,113	Perbellini et al. 2003
		Raucher	15	0,154	n. a.	0,046–0,487	
		Erwachsene	26	< 0,4	n. a.	< 0,4–2,61	Alonso et al. 2012
		Nichtraucher	24	0,180	n. a.	0,105–0,430	Andreoli et al. 1999
	Urin	Nichtraucher	16	0,123	n. a.	n. a.	Fustinoni et al. 1999
		Raucher	16	0,441	n. a.	n. a.	
		Nichtraucher	24	0,089	n. a.	0,045–0,353	Andreoli et al. 1999
		Nichtraucher	10	0,175	n. a.	< 0,050–0,291	Brčić Karačonji und Skender 2007
		Raucher	10	0,502	n. a.	0,245–0,635	
		Nichtraucher	15	0,066	n. a.	0,024–0,248	Perbellini et al. 2002
		Raucher	10	0,125	n. a.	0,042–0,409	
		Nichtraucher	10	21,4	n. a.	2,8–70,1	Song et al. 2017
		Nichtraucher	65	0,094	0,180	0,056–0,180^b)^	Fustinoni et al. 2010
		Raucher	43	0,436	2,70	0,085–2,70^b)^	
		gesunde Männer	90	0,146	2,23	0,043–2,23^b)^	Campo et al. 2016
		Nichtraucher	46	0,067	n. a.	0,026–0,531	Perbellini et al. 2003
		Raucher	15	0,238	n. a.	0,045–1,099	
1,3-Butadien	Urin	Nichtraucher	46	0,0011	n. a.	< 0,001–0,0024	Perbellini et al. 2003
		Raucher	15	0,0031	n. a.	0,0012–0,0089	
	Blut	gesunde Erwachsene	28	0,009^a)^	n. a.	< 0,003–0,015	Mochalski et al. 2013
		Nichtraucher	46	0,0019	n. a.	< 0,0005–0,0035	Perbellini et al. 2003
		Raucher	15	0,0060	n. a.	0,0012–0,0502	
*n‑*Butan	Blut	gesunde Erwachsene	28	0,020^a)^	n. a.	< 0,008–0,027	Mochalski et al. 2013
2,3-Butandion (Dimethylglyoxal)	Blut	gesunde Erwachsene	28	< 0,34^a)^	n. a.	< 0,34	Mochalski et al. 2013
2-Butanon (Methylethylketon)	Blut	gesunde Erwachsene	28	2,52^a)^	n. a.	0,61–5,19	Mochalski et al. 2013
*n*-Butylbenzol	Urin	Nichtraucher	10	4,8	n. a.	3,1–9,1	Song et al. 2017
*sec*-Butylbenzol	Urin	Nichtraucher	10	5,1	n. a.	4,4–5,7	Song et al. 2017
3-Caren	Blut	gesunde Erwachsene	28	0,46^a)^	n. a.	< 0,12–0,60	Mochalski et al. 2013
*n*-Decan	Blut	gesunde Erwachsene	28	0,44^a)^	n. a.	< 0,043–1,88	Mochalski et al. 2013
Dichlormethan (Methylenchlorid)	Urin	gesunde Erwachsene	120	0,64	n. a.	0,27–2,22	Poli et al. 2005
Diisopropylether	Blut	gesunde Erwachsene	3	0,0057	n. a.	< 0,0006–0,044	Silva et al. 2008
2,3-Dimethylbutan	Blut	gesunde Erwachsene	28	< 0,005^a)^	n. a.	< 0,005	Mochalski et al. 2013
2,5-Dimethylfuran	Urin	Nichtraucher	46	0,039	n. a.	< 0,005–0,290	Perbellini et al. 2003
		Raucher	15	0,161	n. a.	0,019–0,525	
	Blut	gesunde Erwachsene	28	0,039^a)^	n. a.	< 0,002–0,063	Mochalski et al. 2013
		Nichtraucher	46	< 0,005	n. a.	< 0,005–0,040	Perbellini et al. 2003
		Raucher	15	0,076	n. a.	< 0,005–0,373	
		Erwachsene	28	< 0,1	n. a.	< 0,1	Alonso et al. 2012
Dimethylselenid	Blut	gesunde Erwachsene	28	0,028^a)^	n. a.	< 0,003–0,055	Mochalski et al. 2013
Dimethylsulfid	Blut	gesunde Erwachsene	28	0,52^a)^	n. a.	0,12–2,04	Mochalski et al. 2013
Ethylacetat	Blut	gesunde Erwachsene	28	0,24^a)^	n. a.	< 0,009–0,44	Mochalski et al. 2013
Ethylbenzol	Blut	Nichtraucher	15	0,145	n. a.	< 0,022–0,496	Perbellini et al. 2002
		Raucher	10	0,148	n. a.	0,063–0,596	
		gesunde Erwachsene	28	0,208^a)^	n. a.	n. a.	Mochalski et al. 2013
		Erwachsene	28	< 0,2	n. a.	< 0,2–0,69	Alonso et al. 2012
		Nichtraucher	24	0,213	n. a.	0,145–0,880	Andreoli et al. 1999
	Urin	Nichtraucher	16	0,030	n. a.	n. a.	Fustinoni et al. 1999
		Raucher	16	0,057	n. a.	n. a.	
		gesunde Männer	90	0,072	0,165	0,033–0,165^b)^	Campo et al. 2016
		Nichtraucher	24	0,073	n. a.	0,037–0,141	Andreoli et al. 1999
		Nichtraucher	10	0,121	n. a.	< 0,035–0,175	Brčić Karačonji und Skender 2007
		Raucher	10	0,165	n. a.	0,070–0,353	
Ethylbenzol	Urin	Nichtraucher	15	0,0085	n. a.	< 0,017–0,047	Perbellini et al. 2002
		Raucher	10	0,0085	n. a.	< 0,017–0,037	
		Nichtraucher	65	0,073	0,130	0,016–0,130^b)^	Fustinoni et al. 2010
		Raucher	43	0,074	0,123	0,025–0,123^b)^	
Ethyl‑*tert‑*butylether	Blut	gesunde Erwachsene	3	< 0,0006	n. a.	< 0,0006–0,00066	Silva et al. 2008
	Urin	Nichtraucher	65	< 0,015	0,024	< 0,015–0,024^b)^	Fustinoni et al. 2010
		Raucher	43	< 0,015	0,025	< 0,015–0,025^b)^	
		gesunde Männer	90	< 0,015	0,030	< 0,015–0,030^b)^	Campo et al. 2016
Ethylmethylsulfid	Blut	gesunde Erwachsene	28	0,030^a)^	n. a.	< 0,005–0,062	Mochalski et al. 2013
Ethylvinylether	Blut	gesunde Erwachsene	28	0,009^a)^	n. a.	< 0,003–0,017	Mochalski et al. 2013
Eucalyptol	Blut	gesunde Erwachsene	28	1,00^a)^	n. a.	< 0,12–1,54	Mochalski et al. 2013
Furan	Blut	gesunde Erwachsene	28	0,007^a)^	n. a.	< 0,0008–0,025	Mochalski et al. 2013
2-Heptanon	Blut	gesunde Erwachsene	28	0,31^a)^	n. a.	0,069–0,65	Mochalski et al. 2013
4-Heptanon	Blut	gesunde Erwachsene	28	0,095^a)^	n. a.	0,023–0,25	Mochalski et al. 2013
*cis*,*trans*-2,4-Hexadien	Blut	gesunde Erwachsene	28	< 0,002^a)^	n. a.	< 0,002	Mochalski et al. 2013
*n*-Hexan	Blut	gesunde Erwachsene	28	0,015^a)^	n. a.	< 0,002–0,049	Mochalski et al. 2013
2-Hexanon	Blut	gesunde Erwachsene	28	0,036^a)^	n. a.	< 0,015–0,050	Mochalski et al. 2013
3-Hexanon	Blut	gesunde Erwachsene	28	< 0,015^a)^	n. a.	< 0,015–0,048	Mochalski et al. 2013
1-Hexen	Blut	gesunde Erwachsene	28	0,007^a)^	n. a.	< 0,002–0,018	Mochalski et al. 2013
Isopren	Blut	gesunde Erwachsene	28	1,00^a)^	n. a.	0,24–2,32	Mochalski et al. 2013
4-Isopropyltoluol (*p*‑Cymol)	Blut	gesunde Erwachsene	28	0,15^a)^	n. a.	0,04–0,73	Mochalski et al. 2013
(*m* + *p*)-Kresol	Urin	Nichtraucher	10	23,0	n. a.	3,8–92,2	Song et al. 2017
*o*-Kresol	Urin	Nichtraucher	10	2,6	n. a.	2,1–4,8	Song et al. 2017
Limonen	Blut	gesunde Erwachsene	28	1,27^a)^	n. a.	0,13–5,80	Mochalski et al. 2013
Menthon	Blut	gesunde Erwachsene	28	0,76^a)^	n. a.	< 0,093–1,20	Mochalski et al. 2013
Methylacetat	Blut	gesunde Erwachsene	28	2,26^a)^	n. a.	0,25–11,6	Mochalski et al. 2013
2-Methylbutan (Isopentan)	Blut	gesunde Erwachsene	28	0,053^a)^	n. a.	< 0,005–0,152	Mochalski et al. 2013
2-Methyl-1-buten	Blut	gesunde Erwachsene	28	< 0,004^a)^	n. a.	< 0,004	Mochalski et al. 2013
Methyl-*tert*‑butylether (2‑Methoxy-2‑methylpropan)	Blut	gesunde Erwachsene	3	0,0029	n. a.	0,0022–0,0035	Silva et al. 2008
	Urin	Nichtraucher	65	0,046	0,152	0,020–0,152^b)^	Fustinoni et al. 2010
		Raucher	43	0,051	0,097	0,023–0,097^b)^	
		gesunde Männer	90	0,070	0,219	< 0,010–0,219^b)^	Campo et al. 2016
2-Methylfuran	Blut	gesunde Erwachsene	28	0,012^a)^	n. a.	< 0,0008–0,021	Mochalski et al. 2013
3-Methylfuran	Blut	gesunde Erwachsene	28	0,005^a)^	n. a.	< 0,001–0,008	Mochalski et al. 2013
2-Methylhexan	Blut	gesunde Erwachsene	28	0,013^a)^	n. a.	< 0,002–0,057	Mochalski et al. 2013
4-Methyloctan	Blut	gesunde Erwachsene	28	0,12^a)^	n. a.	< 0,019–0,31	Mochalski et al. 2013
2-Methylpentan	Blut	gesunde Erwachsene	28	0,030^a)^	n. a.	< 0,007–0,046	Mochalski et al. 2013
4-Methyl-1-penten	Blut	gesunde Erwachsene	28	< 0,003^a)^	n. a.	< 0,003	Mochalski et al. 2013
2-Methylpropan (Isobutan)	Blut	gesunde Erwachsene	28	0,07^a)^	n. a.	< 0,013–0,09	Mochalski et al. 2013
2-Methyl-1-propen (Isobuten)	Blut	gesunde Erwachsene	28	0,19^a)^	n. a.	n. a.	Mochalski et al. 2013
2-Methyl-2-propenal	Blut	gesunde Erwachsene	28	< 0,063^a)^	n. a.	< 0,063	Mochalski et al. 2013
Methylpropionat	Blut	gesunde Erwachsene	28	0,25^a)^	n. a.	< 0,012–1,32	Mochalski et al. 2013
Methylpropylsulfid	Blut	gesunde Erwachsene	28	0,40^a)^	n. a.	< 0,004–6,89	Mochalski et al. 2013
1-Methylpyrrol	Blut	gesunde Erwachsene	28	0,039^a)^	n. a.	< 0,008–0,049	Mochalski et al. 2013
*α*-Methylstyrol	Blut	gesunde Erwachsene	28	0,024^a)^	n. a.	< 0,012–0,024	Mochalski et al. 2013
3-Methylthiophen	Blut	gesunde Erwachsene	28	< 0,002^a)^	n. a.	< 0,002–0,004	Mochalski et al. 2013
Methylvinylketon (Butenon)	Blut	gesunde Erwachsene	28	10,9^a)^	n. a.	< 2,8–12,7	Mochalski et al. 2013
Naphthalin	Urin	Nichtraucher	10	9,5	n. a.	2,3–22,9	Song et al. 2017
		Nichtraucher	7	0,048	0,057	0,038–0,057^b)^	Fustinoni et al. 2010
		Raucher	11	0,044	0,266	0,038–0,266^b)^	
*n*-Octan	Blut	gesunde Erwachsene	28	0,15^a)^	n. a.	< 0,005–0,39	Mochalski et al. 2013
*n*-Pentan	Blut	gesunde Erwachsene	28	0,027^a)^	n. a.	< 0,007–0,058	Mochalski et al. 2013
*cis*-1,3-Pentadien	Blut	gesunde Erwachsene	28	< 0,001^a)^	n. a.	< 0,001	Mochalski et al. 2013
*trans*-1,3-Pentadien	Blut	gesunde Erwachsene	28	0,006^a)^	n. a.	< 0,002–0,007	Mochalski et al. 2013
*cis*-Pent-2-en	Blut	gesunde Erwachsene	28	< 0,003^a)^	n. a.	< 0,003	Mochalski et al. 2013
*trans*-Pent-2-en	Blut	gesunde Erwachsene	28	0,009^a)^	n. a.	< 0,003–0,009	Mochalski et al. 2013
2-Pentanon	Blut	gesunde Erwachsene	28	2,99^a)^	n. a.	0,81–9,08	Mochalski et al. 2013
*trans*-3-Penten-2-on	Blut	gesunde Erwachsene	28	0,84^a)^	n. a.	< 0,21–1,71	Mochalski et al. 2013
*α*-Pinen	Blut	gesunde Erwachsene	28	< 0,008^a)^	n. a.	< 0,008	Mochalski et al. 2013
*β*-Pinen	Blut	gesunde Erwachsene	28	0,15^a)^	n. a.	< 0,005–0,20	Mochalski et al. 2013
Propanal	Blut	gesunde Erwachsene	28	0,93^a)^	n. a.	< 0,076–1,68	Mochalski et al. 2013
Propen (Propylen)	Blut	gesunde Erwachsene	28	0,59^a)^	n. a.	0,16–2,59	Mochalski et al. 2013
2‑Propenal (Acrolein)	Blut	gesunde Erwachsene	28	137^a)^	n. a.	< 15,1–376	Mochalski et al. 2013
Propylbenzol	Urin	Nichtraucher	10	4,0	n. a.	2,0–5,8	Song et al. 2017
Pyrazin	Blut	gesunde Erwachsene	28	1,60^a)^	n. a.	< 0,36–2,56	Mochalski et al. 2013
Pyrrol	Blut	gesunde Erwachsene	28	0,070^a)^	n. a.	< 0,001–0,127	Mochalski et al. 2013
Styrol	Blut	gesunde Erwachsene	28	0,037^a)^	n. a.	< 0,010–0,076	Mochalski et al. 2013
		Erwachsene	28	< 0,100	n. a.	< 0,100–0,600	Alonso et al. 2012
Tetrachlorethen	Urin	gesunde Erwachsene	120	0,05	n. a.	0,01–0,70	Poli et al. 2005
Thiophen	Blut	gesunde Erwachsene	28	0,004^a)^	n. a.	< 0,001–0,012	Mochalski et al. 2013
Toluol	Blut	Nichtraucher	15	0,428	n. a.	0,120–6,040	Perbellini et al. 2002
		Raucher	10	0,780	n. a.	0,348–5,148	
		gesunde Erwachsene	28	0,055^a)^	n. a.	< 0,003–0,29	Mochalski et al. 2013
		Erwachsene	28	1,15	n. a.	< 0,2–3,10	Alonso et al. 2012
		Nichtraucher	24	0,285	n. a.	0,105–0,925	Andreoli et al. 1999
	Urin	Nichtraucher	16	0,215	n. a.	n. a.	Fustinoni et al. 1999
		Raucher	16	0,336	n. a.	n. a.	
		Nichtraucher	24	0,280	n. a.	0,155–0,480	Andreoli et al. 1999
		Nichtraucher	10	0,166	n. a.	0,141–0,216	Brčić Karačonji und Skender 2007
		Raucher	10	0,633	n. a.	0,184–0,886	
		Nichtraucher	15	0,416	n. a.	0,143–1,227	Perbellini et al. 2002
		Raucher	10	0,259	n. a.	0,131–0,856	
		Nichtraucher	10	3,6	n. a.	2,3–4,9	Song et al. 2017
		Nichtraucher	65	0,375	0,506	0,092–0,506^b)^	Fustinoni et al. 2010
		Raucher	43	0,437	0,698	0,126–0,698^b)^	
		gesunde Männer	90	0,251	0,738	0,172–0,738^b)^	Campo et al. 2016
Trichlorethen	Urin	gesunde Erwachsene	120	0,22	n. a.	0,02–3,64	Poli et al. 2005
*n*-Undecan	Blut	gesunde Erwachsene	28	0,34^a)^	n. a.	< 0,11–0,41	Mochalski et al. 2013
*m*-Xylol	Blut	Nichtraucher	15	0,535	n. a.	0,092–1,451	Perbellini et al. 2002
		Raucher	10	0,411	n. a.	0,203–1,713	
	Urin	Nichtraucher	15	0,099	n. a.	0,072–0,184	Perbellini et al. 2002
		Raucher	10	0,079	n. a.	0,063–0,171	
(*m* + *p*)-Xylol	Blut	gesunde Erwachsene	28	0,10^a)^	n. a.	< 0,007–1,19	Mochalski et al. 2013
		Erwachsene	28	< 0,300	n. a.	< 0,300–1,750	Alonso et al. 2012
	Urin	Nichtraucher	16	0,108	n. a.	n. a.	Fustinoni et al. 1999
		Raucher	16	0,163	n. a.	n. a.	
		Nichtraucher	10	0,329	n. a.	0,104–0,465	Brčić Karačonji und Skender 2007
		Raucher	10	0,436	n. a.	0,198–0,901	
		Nichtraucher	65	0,124	0,165	0,050–0,165^b)^	Fustinoni et al. 2010
		Raucher	43	0,128	0,215	0,055–0,215^b)^	
		gesunde Männer	90	0,110	0,237	0,063–0,237^b)^	Campo et al. 2016
(*m* + *o* + *p*)-Xylol	Blut	Nichtraucher	24	0,722	n. a.	0,280–1,342	Andreoli et al. 1999
	Urin	Nichtraucher	24	0,220	n. a.	0,120–0,459	Andreoli et al. 1999
*o*-Xylol	Blut	gesunde Erwachsene	28	0,23^a)^	n. a.	< 0,009–0,55	Mochalski et al. 2013
		Erwachsene	28	< 0,2	n. a.	< 0,2	Alonso et al. 2012
	Urin	Nichtraucher	16	0,043	n. a.	n. a.	Fustinoni et al. 1999
		Raucher	16	0,061	n. a.	n. a.	
		Nichtraucher	10	0,042	n. a.	< 0,042–0,104	Brčić Karačonji und Skender 2007
		Raucher	10	0,096	n. a.	0,060–0,213	
		Nichtraucher	65	0,044	0,060	0,017–0,060^b)^	Fustinoni et al. 2010
		Raucher	43	0,042	0,079	0,019–0,079^b)^	
		gesunde Männer	90	0,037	0,082	0,020–0,082^b)^	Campo et al. 2016

^a)^ Mittelwert

^b)^ 5.–95. Perzentil

**Tab.9:** US-amerikanische Referenzwerte der beruflich nicht belasteten Allgemeinbevölkerung, deren Parameter mit Headspace-Methoden erfasst werden können

Substanz (Synonym)	Analyt	Matrix	Studienkollektiv	Referenzwert^a)^ [μg/l]	Survey-Zeitraum	Literatur
Benzol	Benzol	Blut	Allgemeinbevölkerung >18 a, Raucher	0,642	2015/2016	NCEH 2021 a
			Allgemeinbevölkerung >18 a, Nichtraucher	0,067	2015/2016	NCEH 2021 a
Chlorbenzol	Chlorbenzol	Blut	Allgemeinbevölkerung >18 a, Raucher	< NWG (0,011)	2015/2016	NCEH 2021 a
			Allgemeinbevölkerung >18 a, Nichtraucher	< NWG (0,011)	2015/2016	NCEH 2021 a
1,1-Dichlorethan	1,1-Dichlorethan	Blut	Allgemeinbevölkerung >20 a	< NWG (0,010)	2011/2012	NCEH 2021 b
1,2-Dichlorethan	1,2-Dichlorethan	Blut	Allgemeinbevölkerung >18 a, Raucher	< NWG (0,010)	2015/2016	NCEH 2021 a
			Allgemeinbevölkerung >18 a, Nichtraucher	< NWG (0,010)	2015/2016	NCEH 2021 a
Dichlormethan (Methylenchlorid)	Dichlormethan	Blut	Allgemeinbevölkerung >18 a, Raucher	< NWG (0,250)	2015/2016	NCEH 2021 a
			Allgemeinbevölkerung >18 a, Nichtraucher	< NWG (0,250)	2015/2016	NCEH 2021 a
1,4-Dioxan	1,4-Dioxan	Blut	Allgemeinbevölkerung >18 a, Raucher	< NWG (0,500)	2015/2016	NCEH 2021 a
			Allgemeinbevölkerung >18 a, Nichtraucher	< NWG (0,500)	2015/2016	NCEH 2021 a
Ethylbenzol	Ethylbenzol	Blut	Allgemeinbevölkerung >18 a, Raucher	0,202	2015/2016	NCEH 2021 a
			Allgemeinbevölkerung >18 a, Nichtraucher	0,056	2015/2016	NCEH 2021 a
*n*-Hexan	*n*-Hexan	Blut	Allgemeinbevölkerung >18 a, Raucher	< NWG (0,122)	2015/2016	NCEH 2021 a
			Allgemeinbevölkerung >18 a, Nichtraucher	< NWG (0,122)	2015/2016	NCEH 2021 a
Isopropylbenzol (Cumol)	Isopropylbenzol	Blut	Allgemeinbevölkerung >18 a, Raucher	< NWG (0,040)	2015/2016	NCEH 2021 a
			Allgemeinbevölkerung >18 a, Nichtraucher	< NWG (0,040)	2015/2016	NCEH 2021 a
Methyl-*tert*‑butylether (2‑Methoxy-2‑methylpropan)	Methyl-*tert*‑butylether	Blut	Allgemeinbevölkerung >18 a, Raucher	10,0	2015/2016	NCEH 2021 a
			Allgemeinbevölkerung >18 a, Nichtraucher	< NWG (0,010)	2015/2016	NCEH 2021 a
Methylquecksilber	Methylquecksilber	Blut	Allgemeinbevölkerung >20 a	4,42	2015/2016	NCEH 2025 a
Styrol	Styrol	Blut	Allgemeinbevölkerung >20 a	0,146	2009/2010	NCEH 2025 b
1,1,1,2-Tetrachlorethan	1,1,1,2-Tetrachlorethan	Blut	Allgemeinbevölkerung >18 a, Raucher	< NWG (0,040)	2015/2016	NCEH 2021 a
			Allgemeinbevölkerung >18 a, Nichtraucher	< NWG (0,040)	2015/2016	NCEH 2021 a
1,1,2,2-Tetrachlorethan	1,1,2,2-Tetrachlorethan	Blut	Allgemeinbevölkerung >20 a	< NWG (0,010)	2011/2012	NCEH 2021 b
Tetrachlorethen	Tetrachlorethen	Blut	Allgemeinbevölkerung >18 a, Raucher	0,056	2015/2016	NCEH 2021 a
			Allgemeinbevölkerung >18 a, Nichtraucher	0,084	2015/2016	NCEH 2021 a
Tetrachlormethan (Tetrachlorkohlenstoff)	Tetrachlormethan	Blut	Allgemeinbevölkerung >18 a, Raucher	< NWG (0,005)	2015/2016	NCEH 2021 a
			Allgemeinbevölkerung >18 a, Nichtraucher	< NWG (0,005)	2015/2016	NCEH 2021 a
Tetrahydrofuran	Tetrahydrofuran	Blut	Allgemeinbevölkerung >18 a, Raucher	< NWG (0,125)	2015/2016	NCEH 2021 a
			Allgemeinbevölkerung >18 a, Nichtraucher	< NWG (0,125)	2015/2016	NCEH 2021 a
Toluol	Toluol	Blut	Allgemeinbevölkerung >18 a, Raucher	1,50	2015/2016	NCEH 2021 a
			Allgemeinbevölkerung >18 a, Nichtraucher	0,312	2015/2016	NCEH 2021 a
1,1,1-Trichlorethan	1,1,1-Trichlorethan	Blut	Allgemeinbevölkerung >18 a, Raucher	< NWG (0,010)	2015/2016	NCEH 2021 a
			Allgemeinbevölkerung >18 a, Nichtraucher	< NWG (0,010)	2015/2016	NCEH 2021 a
1,1,2-Trichlorethan	1,1,2-Trichlorethan	Blut	Allgemeinbevölkerung >20 a	< NWG (0,010)	2011/2012	NCEH 2021 b
Trichlorethen	Trichlorethen	Blut	Allgemeinbevölkerung >18 a, Raucher	< NWG (0,012)	2015/2016	NCEH 2021 a
			Allgemeinbevölkerung >18 a, Nichtraucher	< NWG (0,012)	2015/2016	NCEH 2021 a
Trichlormethan	Trichlormethan	Blut	Allgemeinbevölkerung >18 a, Raucher	0,053	2015/2016	NCEH 2021 a
			Allgemeinbevölkerung >18 a, Nichtraucher	0,047	2015/2016	NCEH 2021 a
(*m* + *p*)-Xylol	(*m* + *p*)-Xylol	Blut	Allgemeinbevölkerung >18 a, Raucher	0,582	2015/2016	NCEH 2021 a
			Allgemeinbevölkerung >18 a, Nichtraucher	0,213	2015/2016	NCEH 2021 a
*o*‑Xylol	*o*‑Xylol	Blut	Allgemeinbevölkerung >18 a, Raucher	0,106	2015/2016	NCEH 2021 a
			Allgemeinbevölkerung >18 a, Nichtraucher	0,059	2015/2016	NCEH 2021 a

^a)^ 95. Perzentil

Abkürzungen siehe Abkürzungsverzeichnis

## Figures and Tables

**Tab.10:** Versuchsbedingungen für die Untersuchung der Lagerungsstabilität verschiedener Lösungsmittel

Material	Gepooltes EDTA-Blut				Gepoolter Urin			
**Probenvolumen**	1 ml	1 ml	1 ml	1 ml	1 ml	1 ml	1 ml	1 ml
**NaCl**	1 g	1 g	**–**	**–**	1 g	1 g	**–**	**–**
**Lagerungsgefäß **	HS-Bördelrandgläschen		Blutröhrchen (Glas)	Schraubreagenzglas	HS-Bördelrandgläschen		Urinbecher	Schraubreagenzglas
**HgCl_2_‑Lösung**	100 μl	**–**	**–**	**–**	100 μl	**–**	**–**	**–**
**Temperatur**	22 °C	22 °C	4 °C	–20 °C	22 °C	22 °C	4 °C	–20 °C
**Dauer**	14 d	14 d	14 d	14 d	14 d	14 d	14 d	14 d
**Messung in**	Lagerungsgefäß		HS-Bördelrandgläschen		Lagerungsgefäß		HS-Bördelrandgläschen	

**Table:** 

Äquilibrierung:	20 min bei 70 °C
Temperatur der Transferleitung zum GC:	140 °C
Loop-Temperatur:	130 °C

**Table:** 

Kapillarsäule:	Fused Silica (Restek Rxi‑624Sil MS, 60 m × 0,25 mm × 1,4 μm)
Temperaturprogramm:	Ausgangstemperatur 35 °C für 2 min, Anstieg mit 10 °C/min auf 190 °C, dann Anstieg mit 25 °C/min auf 300 °C, 4 min bei Endtemperatur
Injektortemperatur:	250 °C
Trägergas:	Helium 5.0; Flussrate: 1,7 ml/min
Injektion:	Split: 6 ∶ 1

**Table:** 

Ionisationsart:	EI+
Interface-Temperatur:	280 °C
Quellen-Temperatur:	230 °C
Detektionsmodus:	Single Ion Monitoring (SIM)

**Tab.11:** Retentionszeiten und Massenfragmente der gemessenen Analyten

Analyt	Retentionszeit [min]	Q1 (*m/z*)	Q2 (*m/z*)
Aceton	6,34	58	43
2-Propanol	6,49	45	43
Acetonitril	6,67	41	39
1-Propanol	7,86	59	42
2‑Butanon (Methylethylketon)	8,56	72	57
Tetrahydrofuran	9,01	72	71
Isobutanol	9,41	74	43
1-Butanol	10,2	56	41
Cyclohexanon	13,0	98	55
Cyclohexanol	15,2	82	57

**Abb. 6:**
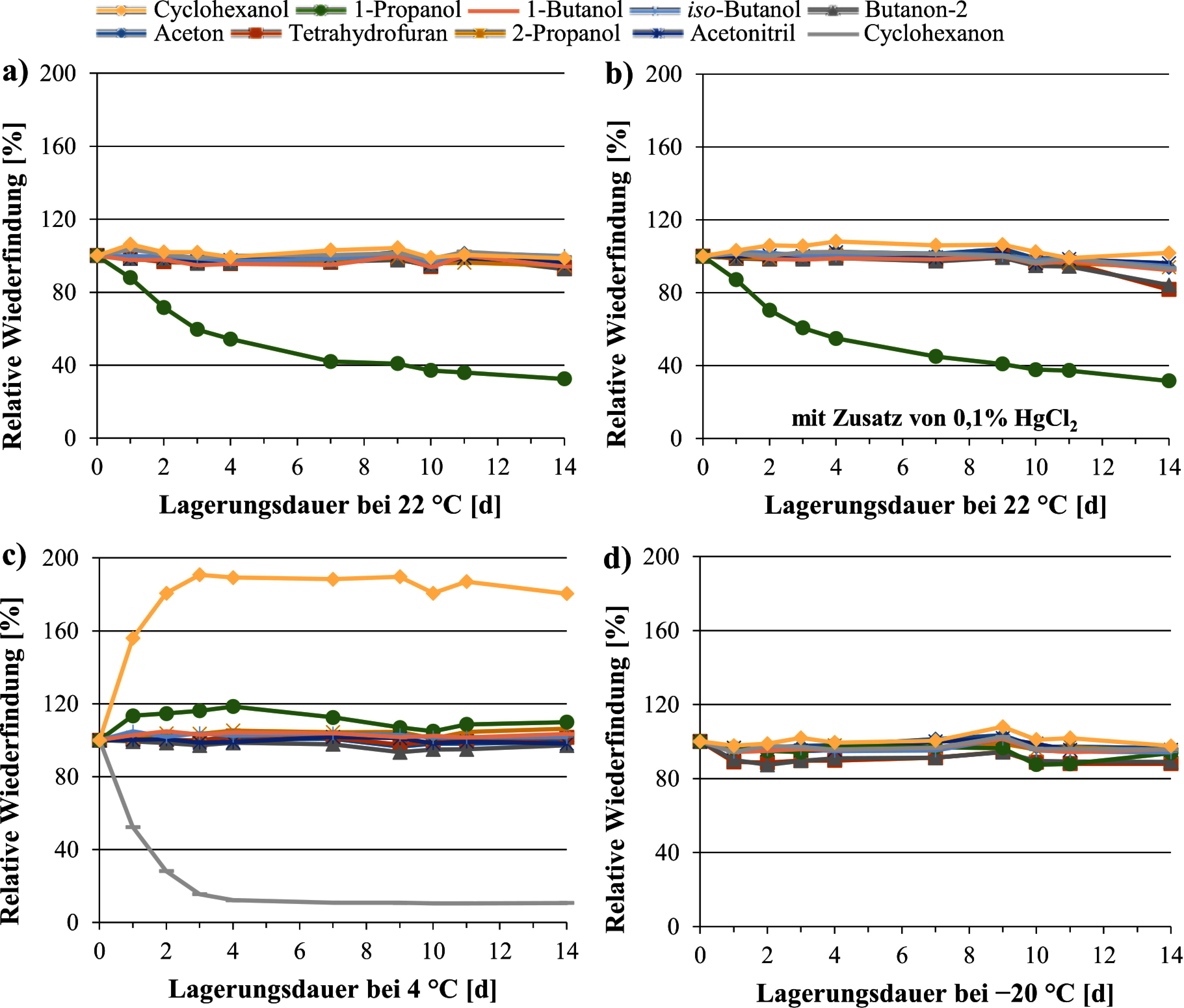
Relative Wiederfindungen der Analyten nach Lagerung der dotierten Blutproben: a) Lagerung in Headspace-Bördelrandgläschen bei 22 °C unter Zusatz von 1 g NaCl, b) Lagerung in Headspace-Bördelrandgläschen bei 22 °C unter Zusatz von 1 g NaCl und 100 μl 0,1%iger HgCl_2_-Lösung, c) Lagerung in einem EDTA-Blutentnahmeröhrchen aus Glas bei 4 °C sowie d) Lagerung in Schraubreagenzgläsern bei −20 °C

**Abb. 7:**
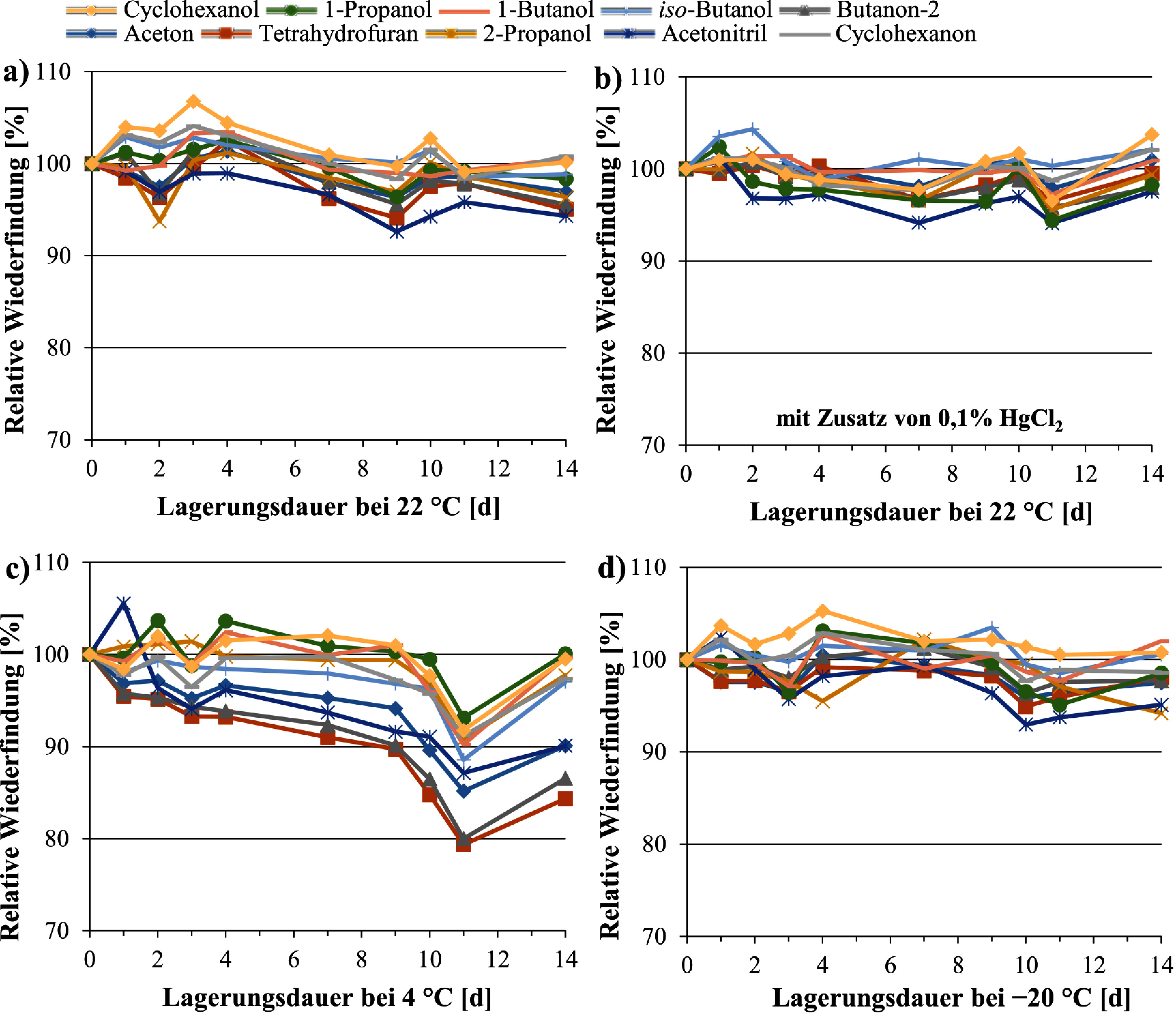
Relative Wiederfindungen der Analyten nach Lagerung der dotierten Urinproben: a) Lagerung in Headspace-Bördelrandgläschen bei 22 °C unter Zusatz von 1 g NaCl, b) Lagerung in Headspace-Bördelrandgläschen bei 22 °C unter Zusatz von 1 g NaCl und 100 μl 0,1%iger HgCl_2_-Lösung, c) Lagerung in einem Urinbecher bei 4 °C sowie d) Lagerung in Schraubreagenzgläsern bei −20 °C

**Table:** 

ACGIH	American Conference of Governmental Industrial Hygienists
AGS	Ausschuss für Gefahrstoffe
ANSES	Französische Agentur für Nahrungssicherheit, Umwelt und Arbeitsschutz („*Agence nationale de sécurité sanitaire de l’alimentation, de l’environnement et du travail“*)
BAL	Biomonitoring Aktionslevel („*Biomonitoring Action Level“*)
BAR	Biologischer Arbeitsstoff‐Referenzwert
BAT	Biologischer Arbeitsstoff‐Toleranzwert
BAT‑Suva	Biologischer Arbeitsstoff‐Toleranzwert festgelegt von der Suva in der Schweiz
BEI^®^	Biologischer Expositionsindex („*Biological Exposure Index“*)
BGV	Biologischer Leitwert („*Biological Guidance Value“*)
BGW	Biologischer Grenzwert
BLV	Biologischer Grenzwert („*Biological Limit Value“*)
BLW	Biologischer Leitwert
BMGV	Richtwert für die biologische Überwachung („*Biological Monitoring Guidance Value“*)
BTEX	Benzol, Toluol, Ethylbenzol und Xylol-Isomere
2-CP	2-Chlorphenol
CDC	Centers for Disease Control and Prevention
2,4‑DCP	2,4-Dichlorphenol
DDD	Dichlordiphenyldichlorethan
DDE	Dichlordiphenyldichlorethen
DDT	Dichlordiphenyltrichlorethan
ECD	Elektrochemische Detektion
EKA	Expositionsäquivalente für krebserzeugende Arbeitsstoffe
FID	Flammenionisationsdetektor
FIOH	Finnisches Institut für Arbeitsmedizin („*Finnish Institute of Occupational Health“*)
GC-MS	Gaschromatographie mit massenspektrometrischer Detektion
G-EQUAS	German Quality Assessment Scheme
Hb	Hämoglobin
HBM	Human-Biomonitoring
HCB	Hexachlorbenzol
*β*-HCH	*β*-Hexachlorcyclohexan
HNMF	*N*‑Hydroxymethyl-N‑methylformamid
HS-Cryotrapping-GC-MS	Headspace-Cryotrapping-Gaschromatographie mit massenspektrometrischer Detektion
HSE	Health and Safety Executive
HS-GC	Headspace-Gaschromatographie
HS-GC-ECD	Headspace-Gaschromatographie mit elektrochemischer Detektion
HS-GC-FID	Headspace-Gaschromatographie mit Flammenionisationsdetektion
HS-GC-FID-MS	Headspace-Gaschromatographie mit Flammenionisationsdetektion und massenspektrometrischer Detektion
HS-GC-MS	Headspace-Gaschromatographie mit massenspektrometrischer Detektion
HS-GC-MS/MS	Headspace-Gaschromatographie mit tandemmassenspektrometrischer Detektion
HS-GC-NPD	Headspace-Gaschromatographie mit Stickstoff-Phosphor-Detektion
HS-GC-TCD	Headspace-Gaschromatographie mit thermischem Leitfähigkeitsdetektor
HS-NTD-GC-FID	Headspace-Needle-Trap-Device-Gaschromatographie mit Flammenionisationsdetektion
HS-NTD-GC-MS	Headspace-Needle-Trap-Device-Gaschromatographie mit massenspektrometrischer Detektion
HSSE	Headspace Sorptive Extraction
HSSE-GC-MS	Headspace-Sorptive-Extraction-Gaschromatographie mit massenspektrometrischer Detektion
HS-SPME	Headspace-Solid Phase Micro Extraction
HS-SPME-GC-FID	Headspace-Solid Phase Micro Extraction-Gaschromatographie mit Flammenionisationsdetektion
HS-SPME-GC-HRMS	Headspace-Solid Phase Micro Extraction-Gaschromatographie mit High Resolution massenspektrometrischer Detektion
HS-SPME-GC-IT/MS	Headspace-Solid Phase Micro Extraction-Gaschromatographie mit Ionenfalle und massenspektrometrischer Detektion
HS-SPME-GC-MS	Headspace-Solid Phase Micro Extraction-Gaschromatographie mit massenspektrometrischer Detektion
HTP	Als schädlich bekannte Konzentrationen („*Haitallisiksi tunnetut pitoisuudet“*)
ISTD	Interner Standard
ITEX	In-tube Extraktionstechnik
JSOH	Japanische Gesellschaft für Arbeitsmedizin („*Japan Society for Occupational Health“*)
MS	Massenspektrometrisch
MTBE	Methyl-*tert*-butylether
n. a.	Nicht angegeben
NHANES	National Health and Nutrition Examination Survey
NWG	Nachweisgrenze
OEL-B	Grenzwert für die Exposition am Arbeitsplatz auf Grundlage des biologischen Monitorings („*Occupational Exposure Limit based on Biological Monitoring“*)
PCB	Polychlorierte Biphenyle
PCP	Pentachlorphenol
PDMS	Polydimethylsiloxan
POP	Persistenter organischer Schadstoff („*Persistent Organic Pollutant“*)
PT-HS-GC-HRMS	Purge & Trap-Headspace-Gaschromatographie mithochauflösender massenspektrometrischer Detektion
PT-HS-GC-MS	Purge & Trap-Headspace-Gaschromatographie mit massenspektrometrischer Detektion
PT-HS-GC-PID	Purge & Trap-Headspace-Gaschromatographie mit Photoionisationsdetektion
RAC	Ausschuss für Risikobeurteilung der ECHA („*Committee for Risk Assessment“*)
SBSE	Stir-Bar Sorptive Extraction
SCOEL	Wissenschaftlicher Ausschuss für Grenzwerte berufsbedingter Exposition („*Scientific Committee on Occupational Exposure Limits“*)
SDME	Single-Drop Micro Extraction
SPDE	Solid Phase Dynamic Extraction
SPME	Solid Phase Micro Extraction
2,3,4,6‑TeCP	2,3,4,6-Tetrachlorphenol
2,4,6‑TCP	2,4,6-Trichlorphenol
THF	Tetrahydrofuran
TLHS-DAI-GC-ECD	Thin-Layer-Headspace-Direct-Aqueous-Injection-Gaschromatographie mit elektrochemischer Detektion
VBR	Biologische Referenzwerte („*valeurs biologiques de référence“*)
VGÜ-Grenzwert	Verordnung über die Gesundheitsüberwachung am Arbeitsplatz
VLB	Biologische Grenzwerte *(„valeurs limites biologiques“*)
VOC	Flüchtige organische Verbindung („*volatile organic compound“*)

## Anhang

### Bestimmung der Lagerstabilität verschiedener Lösungsmittel in Blut und Urin

Proben für die Headspace-Analytik sollten grundsätzlich so bald wie möglich nach Probenahme analysiert werden. In der täglichen Praxis sind Verzögerungen aufgrund der Probenlogistik (z. B. Probenahme vor dem Wochenende, verspätete Probenzustellung) oder der Analytik (hohes Probenaufkommen, Geräteausfall, Personalengpässe etc.) aber nicht auszuschließen.

Um den Einfluss der Transport- und Lagerungsbedingungen auf die Analytenwiederfindung zu untersuchen, wurden Lagerungsversuche durchgeführt. Dazu wurden Blut- und Urinproben mit zehn verschiedenen Lösungsmittel (Aceton, Tetrahydrofuran, 2‑Butanon (Methylethylketon), 2‑Propanol, Acetonitril, 1‑Propanol, Isobutanol, 1‑Butanol, Cyclohexanon und Cyclohexanol) dotiert und unterschiedlich gelagert. Die untersuchten Lösungsmittel orientierten sich dabei an dem Lösungsmittelspektrum, das vom durchführenden Labor auch in der Routine bestimmt wurde.

Als Untersuchungsmatrix wurde gepooltes EDTA-Blut sowie gepoolter Urin von beruflich nicht gegen diese Lösungsmittel exponierten Personen verwendet. Das Material wurde mit den zehn Analyten in einer Konzentration von jeweils 10 mg/l dotiert und in Headspace-Bördelrandgläschen, Schraubreagenzgläser oder die jeweiligen Probenahmegefäße aliquotiert. Zu einigen Proben wurde Quecksilber(II)chlorid zur Hemmung von bakteriellem Aufwuchs zugesetzt. Die Proben wurden über einen Zeitraum von 14 Tagen bei unterschiedlichen Temperaturen gelagert und in diesem Zeitraum zehnmal mittels Headspace‑GC‑MS untersucht. Die in den EDTA-Blutentnahmeröhrchen, Urinbechern bzw. Schraubreagenzgläsern gelagerten Proben wurden vor der Messung in Headspace-Bördelrandgläschen pipettiert. Die unterschiedlichen Versuchsbedingungen sind in Tabelle 10 zusammengestellt.

### Messbedingungen

Die analytische Bestimmung erfolgte an einem HS-GC-MS-Gerät der Firma Agilent bestehend aus einem Headspace-Probennehmer (Agilent G1888), einem Gaschromatographen (Agilent GC 7890A) und einem massenselektiven Detektor (Agilent 5975C).

#### Massenspektrometrie

Die Retentionszeiten und die Massenfragmente (Q1, Q2) der Analyten sind in Tabelle 11 aufgelistet:

### Messergebnisse

Die Hintergrundgehalte in den eingesetzten Blut- und Urinproben wurden gemessen und von den Analytgehalten der dotierten Proben abgezogen, so dass die dargestellten Ergebnisse blindwertkorrigiert sind. Die Quantifizierung der Proben erfolgte mit externer Kalibrierung. Die relativen Wiederfindungen der Analyten in den Blutproben sind in Abbildung 6, die in den Urinproben in Abbildung 7 dargestellt.

### Diskussion der Ergebnisse

#### Lösungsmittel in Blut

Bei den bei Raumtemperatur in verschlossenen Headspace-Gefäßen gelagerten Proben zeigte sich über den gewählten Zeitraum kein signifikanter Verlust der Lösungsmittel. Einzige Ausnahme bildete 1‑Propanol, dessen Konzentration kontinuierlich bis auf etwa 30 % der Ausgangskonzentration absank. Als mögliche Ursache kommen enzymatische Restaktivitäten im Blut in Frage, die eine Oxidation zu 1-Propanal und nachfolgend zu Propionsäure bewirkt haben könnten. Hierfür könnte die durch den NaCl-Zusatz ausgelöste Hämolyse mit Freisetzung der Erythrozytenbestandteile (z. B. Häm) infrage kommen. Die zur Unterbindung von enzymatischen und/oder bakteriellen Einflüssen zu den bei 22 °C gelagerten Proben zugesetzte HgCl_2_‑Lösung zeigte keinen Effekt.

Ganz andere Auswirkungen wurden bei der Lagerung der dotierten Blutproben in den Blutentnahmeröhrchen beobachtet. Während die meisten Lösungsmittel keine signifikante Konzentrationsabnahme in den bei 4 °C gelagerten Proben zeigten, war bei Cyclohexanon eine deutliche Abnahme der Konzentration zu sehen, da es zu Cyclohexanol reduziert wurde. Als Ursache dieser Reduktion, die bei den bei −20 °C gelagerten Proben nicht zu beobachten war, werden enzymatische Restaktivitäten vermutet. Bei den bei 22 °C gelagerten Proben trat die Reduktion des Cyclohexanons – wahrscheinlich wegen des zugesetzten Natriumchlorids – nicht auf.

#### Lösungsmittel in Urin

Ein signifikanter Analytenverlust war in den dotierten Poolurinproben unter den gewählten Lagerungsbedingungen nicht erkennbar. Sowohl in den direkt in Headspace‑Gefäßen abgefüllten Proben, als auch in den bei −20 °C gelagerten Proben lag die relative Wiederfindung zwischen 93 und 107 %. Die Analytwiederfindungen in den mit und ohne HgCl_2_‑Zusatz gelagerten Proben unterschieden sich nicht.

Im Vergleich dazu ergaben sich bei der bei 4 °C im Urinbecher gelagerten Urinprobe Analytenverluste von bis zu 20 %. Bei diesen Messungen kann nicht ausgeschlossen werden, dass bei der wiederholten Entnahme von Probenaliquoten Verluste durch Ausgasen der Lösungsmittel aufgetreten sind. Aber auch bei diesen Lagerungsbedingungen können die Analytwiederfindungen über den Zeitraum von sieben Tagen als gut bezeichnet werden.

### Zusammenfassung

Unter den hier getesteten Lagerungsbedingungen waren die untersuchten Lösungsmittel in den dotierten Blutproben bei −20 °C für 14 Tage stabil. Bei Raumtemparatur baute sich auch unter Zugabe von HgCl_2_ 1‑Propanol ab, ohne Zusatz von NaCl baute sich Cyclohexanon ab.

In den Urinproben sind die Analytwiederfindungen sowohl bei Lagerung der Proben in Headspace‑Gefäßen bei Raumtemperatur als auch bei Lagerung der Proben in Schraubreagenzgläsern bei −20 °C im Gefrierschrank als gut zu bezeichnen. Bei der im Kühlschrank im Urinbecher gelagerten Probe zeigten sich für eine Vielzahl der Analyten leicht schwankende Wiederfindungsraten, es empfiehlt sich daher eine Bestimmung der Analyten innerhalb von sieben Tagen.

### Allgemeine Hinweise

Zur Bewertung der unterschiedlichen Blutentnahmesysteme wurde der Einsatz von Vacutainern^®^ als Alternative zu Monovetten intensiv geprüft. Bei der Untersuchung mehrerer Vacutainer^®^-Typen mit verschiedenen Stopfenarten im Rahmen der BTX-Analytik, wurden Belastungen von bis zu 5 μg Benzol pro Liter Blut detektiert (Bader et al. 1994). Es konnte der Nachweis erbracht werden, dass sich die Leerwerte durch Anwendung speziell präparierter Vacutainer^®^-Stopfen (lila Stopfen auf Vacutainer^®^Art. BD 367655), auf das niedrige Niveau der Monovetten reduzieren ließen.

Bei der Lagerung von Blutproben für die Ananlytik von leichtflüchtigen halogenierten Kohlenwasserstoffen (LHKW) wurde insbesondere für Tetrachlorkohlenstoff beobachtet, dass die Stabilität erhöht werden konnte, wenn die Proben dunkel gelagert wurden.

Bei der Bewertung von auffälligen 2‑Propanolbefunden in Blut ist eine mögliche Kontamination der Punktionsstelle mit 2‑Propanol-haltigen Desinfektionsmitteln in Betracht zu ziehen.

Grundsätzlich müssen bei der Lagerung gebördelter Headspace-Gläschen bei 4 °C insbesondere aber bei −20 °C die materialbedingten unterschiedlichen Ausdehnungskoeffizienten berücksichtigt werden. Es wurde beobachtet, dass sich die Aluminium-Verschlusskappen der Headspace-Gefäße bereits am nächsten Tag frei drehen ließen. Ein Umstand, der zu möglichen Verlusten aber auch zu Kontaminationen führen kann.

Auch Ogawa und Sasahara (2012) stellten fest, dass eine kurzzeitige gekühlte Lagerung von Blutproben (bis zu drei Tage) keine signifikanten Analytenverluste zur Folge hat. In einer anderen Studie, in der Dichlormethan in Urin untersucht wurde, konnten ebenso keine signifikanten Unterschiede zwischen der Lagerung bei Raumtemperatur und im Kühlschrank gefunden werden (Hoffer et al. 2005). Wichtig ist jedoch in jedem Fall die schnelle Überführung der Proben in gasdichte Probengefäße (Hoffer et al. 2005; Ogawa und Sasahara 2012).

Um das Wachstum von Pilzen in bei Raumtemperatur gelagerten Urinproben zu verhindern, kann Natriumchlorid zugesetzt werden. So wurde bei internen Untersuchungen ein Verlust von Methanol in nicht mit Natriumchlorid stabilisierten Urinproben, im Gegensatz zu den mit Natriumchlorid versetzten Proben, ermittelt.
